# Cu-Mediated trifluoromethylation of benzyl, allyl and propargyl methanesulfonates with TMSCF_3_

**DOI:** 10.3762/bjoc.9.322

**Published:** 2013-12-12

**Authors:** Xueliang Jiang, Feng-Ling Qing

**Affiliations:** 1 Key Laboratory of Organofluorine Chemistry, Shanghai Institute of Organic Chemistry, Chinese Academy of Sciences, 345 Lingling Lu, Shanghai 200032, China; 2 College of Chemistry, Chemical Engineering and Biotechnology, Donghua University, 2999 North Renmin Lu, Shanghai 201620, China

**Keywords:** copper, methanesulfonates, organo-fluorine, trifluoromethylation

## Abstract

A Cu-mediated trifluoromethylation of benzyl, allyl and propargyl methanesulfonates with TMSCF_3_ was developed for the first time. This method offers a convenient and economical approach to various trifluoroethyl-containing compounds.

## Introduction

**Scheme 1:**
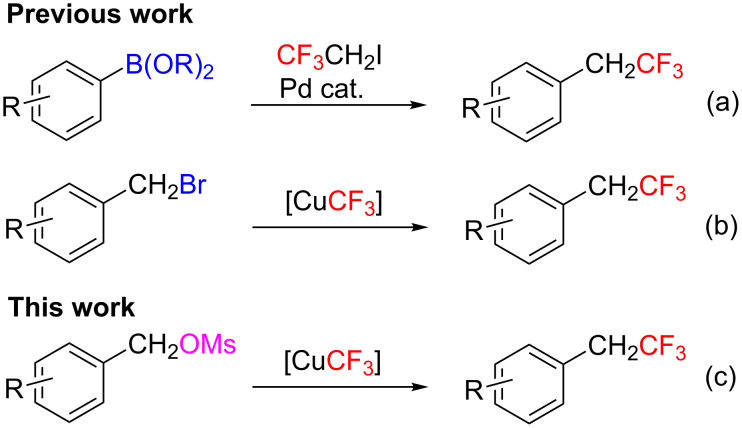
Transition metal-mediated methods for the preparation of (trifluoroethyl)arenes.

Fluorinated organic molecules are extremely important in agrochemicals, pharmaceuticals and materials [1–6]. In recent years, (trifluoroethyl)arenes have drawn increasing attention in medicinal chemistry and related fields [7–9]. Different methods have been developed for the synthesis of (trifluoroethyl)arenes, such as Cl–F exchange of the trichloroethyl derivatives [10], reduction of the (trifluoroethyl)aryl derivatives [11] and addition of 2,2-difluorostyrene derivatives [12]. Compared to these methods, the direct transition metal-mediated trifluoroethylation of arylboronic acids [13–14] (Scheme 1a) and trifluoromethylation of benzyl halides [15–21] (Scheme 1b) are more convenient. Especially trifluoromethylations of benzyl bromides with a [CuCF_3_] species, which are generated from different precursors, are generally employed to afford various (trifluoroethyl)arenes. Although these methods are proven efficient, it is still highly desirable to develop new protocols from economic consideration. In continuation of our research on transition metal-mediated trifluoromethylation [22–27], we report here the first example of Cu-mediated trifluoromethylation of benzyl methanesulfonates (Scheme 1c).

## Results and Discussion

We initiated our investigation by reacting benzyl methanesulfonate **1a** with TMSCF_3_ (2.0 equiv) in the presence of KF (2.0 equiv) and CuI (0.2 equiv) in DMF (2.0 mL) at 60 °C under Ar atmosphere. However, only 17% yield of the desired product **2a** was observed in this case (Table 1, entry 1). The yield was improved to 31% when the reaction was carried out in the presence of 1,10-phenanthroline (phen) (Table 1, entry 2). Increasing the substrate concentration (from 0.1 M to 0.4 M) could further improve the product yield to 49% (Table 1, entries 3 and 4). Other copper salts such as CuBr, CuCl, CuTc and CuOAc, were next screened, but none of them was better than CuI (Table 1, entries 5–8). Interestingly, when the benzyl methanesulfonate reacted with [CuCF_3_] generated in situ from TMSCF_3_ and a stoichiometric amount of CuI (1.1 equiv) without phen, the desired product **2a** was formed in 68% yield (Table 1, entry 9). Decreasing or increasing the amount of CuI resulted in a lower yield (Table 1, entries 10 and 11). The solvent was next screened and, to our delight, the highest yield of the product was achieved when using DMF/HMPA (1:1) as the mixed solvent (Table 1, entry 14).

**Table 1:** Optimization of the reaction conditions.^a^

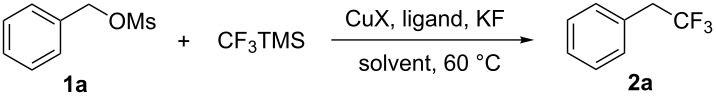				

entry	CuX (equiv)	ligand	solvent	yield of **2a** (%)^b^

1^c^	CuI (0.2)	–	DMF	17
2^c^	CuI (0.2)	phen	DMF	31
3^d^	CuI (0.2)	phen	DMF	32
4	CuI (0.2)	phen	DMF	49
5	CuBr (0.2)	phen	DMF	40
6	CuCl (0.2)	phen	DMF	trace
7^e^	CuTc (0.2)	phen	DMF	trace
8	CuOAc	phen	DMF	trace
9	CuI (1.1)	–	DMF	68
10	CuI (1.5)	–	DMF	66
11	CuI (1.0)	–	DMF	62
12	CuI (1.1)	–	DMSO	38
13	CuI (1.1)	–	HMPA	9
14	CuI (1.1)	–	DMF/HMPA (1:1)	76

^a^Reaction conditions: **1a** (0.2 mmol), ligand (0.2 mmol), TMSCF_3_ (0.4 mmol), KF (0.4 mmol), DMF (0.5 mL), 60 °C, under Ar atmosphere. ^b^Yield was determined by ^19^F NMR using benzotrifluoride as an internal standard. ^c^2.0 mL of DMF. ^d^1.0 mL of DMF. ^e^CuTc is copper(I) thiophene-2-carboxylate.

**Scheme 2:**
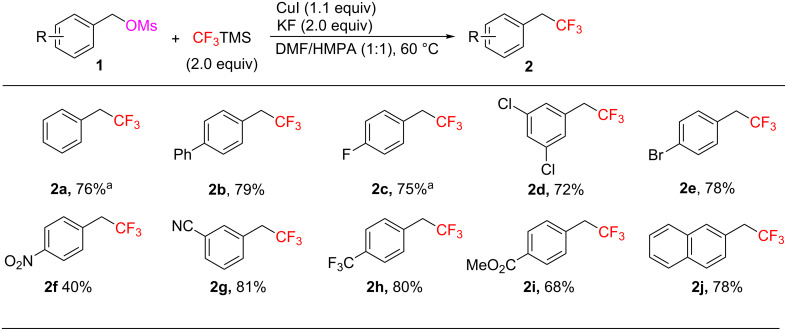
Cu-mediated trifluoromethylation of benzyl methanesulfonates. Reaction conditions: **1** (2.0 mmol), CuI (2.2 mmol), TMSCF_3_ (4.0 mmol), KF (4.0 mmol), DMF/HMPA (1:1, 5.0 mL), 60 °C, under Ar atmosphere; Isolated yield. ^a^Isolated yield after distillation on 10.0 mmol scale.

With the optimal conditions in hand, we next examined the substrate scope of the Cu-mediated trifluoromethylation of benzyl methanesulfonates with TMSCF_3_ (Scheme 2). This method tolerates various functional groups. A wide range of benzyl methanesulfonates bearing electron-withdrawing groups, such as nitro (**1f**), cyano (**1g**), trifluoromethyl (**1h**) and ester (**1i**), as well as electron-donating groups such as phenyl (**1b**), smoothly underwent the transformation, affording the desired products in moderate to good yield. Importantly, both chloro (**1d**) and bromo (**1e**) substituents are also compatible with this method. It is particularly noteworthy that the reaction can be scaled up efficiently. **2a** and **2c** were successfully prepared on 10 mmol scale, indicating the good reliability of the process.

**Scheme 3:**
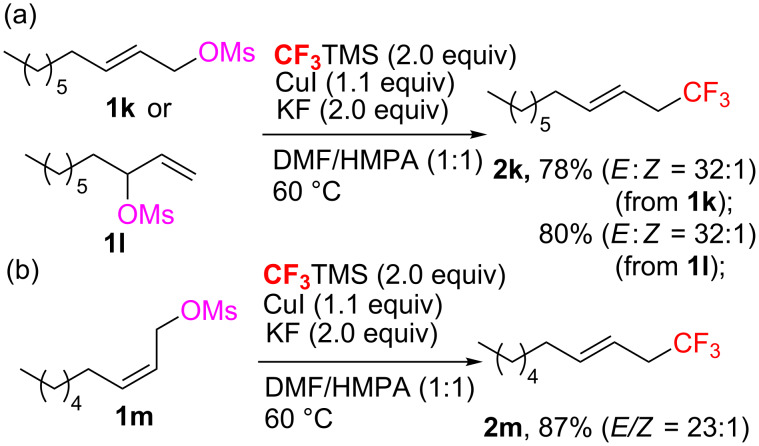
Cu-Mediated trifluoromethylation of allyl methanesulfonates.

The present reaction could also be expanded to the trifluoromethylation of allylic methanesulfonates (Scheme 3). Treatment of the substrate **1k** under the standard reaction conditions afforded the linear trifluoromethylated product **2k** in 78% yield with a trace amount of *Z* isomer. Interesting, the reactions with the allylic methanesulfonates **1l** and **1m** gave the same regioselectivity and stereoselectivity with good yields. These observations indicate that a π-allyl/Cu^ш^ complex might be involved in the C_sp3_–CF_3_ bond formation, but the detailed mechanism remains to be elucidated.

**Scheme 4:**
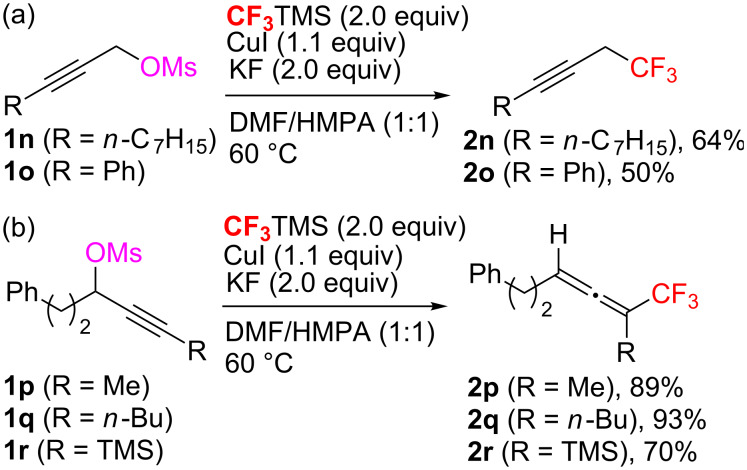
Cu-Mediated trifluoromethylation of propargyl methanesulfonates.

We were next interested in the trifluoromethylation of propargyl methanesulfonate derivates. Both aliphatic and aryl-substituted linear propargyl methanesulfonates under standard reaction conditions afforded the corresponding trifluoromethylated propargylic products in moderate yields (Scheme 4a). However, the reaction of the branched substrates under identical conditions gave the trifluoromethylated allenylic products in good to excellent yields, without any trifluoromethylated propargylic products (Scheme 4b). Thus, this reaction provides an efficient protocol for the synthesis of allenylic-CF_3_ derivatives, which are useful building blocks for pharmaceuticals [28–29].

## Conclusion

In summary, we have developed an efficient copper-mediated trifluoromethylation of benzyl methanesulfonates at the benzylic position under mild conditions. The reaction can be easily scaled up and allows for the efficient synthesis of a series of (trifluoroethyl)arenes with excellent functional group compatibility. Furthermore, the method could also be extended to the trifluoromethylation of allyl and progargyl methanesulfonates, affording the corresponding allylic-, progargylic- and allenylic-CF_3_ derivatives.

## Experimental

**General procedure for the Cu-mediated trifluoromethylation of benzyl methanesulfonates:** CuI (2.2 mmol) and KF (4.0 mmol) were added into a Schlenk tube equipped with a magnetic stirring bar under Ar atmosphere. DMF (5.0 mL) and Me_3_SiCF_3_ (2.0 equiv) were added. After stirring for 20 minutes, the mixture was heated to 60 °C and then benzyl methanesulfonate (2.0 mmol) was added under N_2_ atmosphere. The reaction mixture was kept at 60 °C for 4 hours and then cooled to room temperature. The resulting mixture was diluted with diethyl ether, washed with water and brine, dried over sodium sulfate, and concentrated. The crude products were purified by column chromatography on silica gel to give the products.

## Supporting Information

File 1 Experimental details, characterization data of all products and copies of NMR spectra.
