# Supplementary material for: Cu-Mediated trifluoromethylation of benzyl, allyl and propargyl methanesulfonates with TMSCF3
Source: Beilstein J Org Chem. 2013 Dec 12;9:2862–5. doi: 10.3762/bjoc.9.322 (PMC3869287; doi:10.3762/bjoc.9.322)

Supporting Information

for

Cu-Mediated trifluoromethylation of benzyl, allyl and propargyl methanesulfonates with $TMSCF_3$

Xueliang Jiang[1] and Feng-Ling Qing*[1,2]

Address: [1]Key Laboratory of Organofluorine Chemistry, Shanghai Institute of Organic Chemistry, Chinese Academy of Sciences, 345 Lingling Lu, Shanghai 200032, China and [2]College of Chemistry, Chemical Engineering and Biotechnology, Donghua University, 2999 North Renmin Lu, Shanghai 201620, China

Email: Feng-Ling Qing - flq@mail.sioc.ac.cn

* Corresponding author

Experimental details, characterization data of all products and copies of NMR spectra.

Table of Contents

General information-- pages S2

Preparation of substrates--- pages S2

General procedures for the copper-mediated trifluoromethylation of benzyl methanesulfonates. --pages S6

References---pages S11

NMR Spectra of products--pages S12

General information:

^{1}H NMR (TMS as the internal standard) and ^{19}F NMR spectra ($CFCl_3$ as the outside standard and low field is positive) were recorded on a Bruker AM300 or Bruker AM400 spectrometer. ^{13}C NMR was recorded on a Bruker AM400 spectrometer. Chemical shifts (δ) are reported in ppm, and coupling constants (*J*) are in Hertz (Hz). The following abbreviations were used to explain the multiplicities: s = singlet, d = doublet, t = triplet, q =quartet, m = multiplet, br = broad. Substrates were purchased from commercial sources (Aldrich, Alfa and Chemical Reagent Companies of China) and used as received. Reactions were performed under an atmosphere of nitrogen using glassware that was flame-dried under vacuum. Benzyl alcohols and allylic alcohols were obtained commercially. Propargylic alcohols 1-phenylhex-4-yn-3-ol [1], 1-phenylnon-4-yn-3-ol [2] and 5-phenyl-1-(trimethylsilyl)pent-1-yn-3-ol [3] were prepared according to literature procedures. Compounds **2a** [4], **2b** [5], **2d** [5], **2e** [6], **2f** [7], **2h** [8], **2i-2j** [7] and **2o** [9] have been published previously.

Preparation of substrates:

General procedure for the preparation of benzyl, allylic and propargylic methanesulfonates:

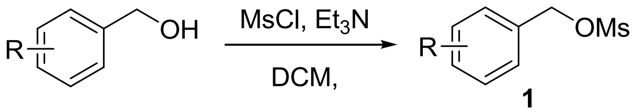

1

To a stirred solution of benzyl alcohol (5.0 mmol) and triethylamine (10 mmol) in DCM (20 mL) under N_2 atmosphere at −20 °C was added methanesulfonyl chloride (7.5 mmol) dropwise. The reaction was stirred for 1 hour (monitored by TLC). The mixture was then allowed to warm to room temperature and washed with water, 2 M HCl and brine, dried over sodium sulfate. The solvent was removed to give the crude product, which was used without purification.

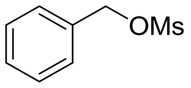

Benzyl methanesulfonate (1a): Colorless oil; yield 91%. ^{1}H NMR (300 MHz, $CDCl_3$) δ ppm 7.41 (m, 5H), 5.24 (s, 2H), 2.90 (s, 3H).

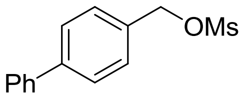

Biphenyl-4-ylmethyl methanesulfonate (1b): White solid; yield 83%. ^{1}H NMR (300 MHz, $CDCl_3$) δ ppm 7.38-7.65 (m, 9H), 5.29 (s, 2H), 2.95 (s, 3H).

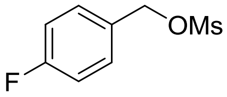

4-Fluorobenzyl methanesulfonate (1c): Colorless oil; yield 91%. ^{1}H NMR (300 MHz, $CDCl_3$) δ ppm 7.39-7.41 (m, 2H), 7.10-7.13 (m, 2H), 5.13 (s, 2H), 2.93 (s, 3H). ^{19}F NMR (282 MHz, $CDCl_3$) δ ppm -111.5 (m, 1F).

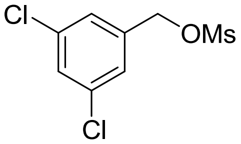

3,5-Dichlorobenzyl methanesulfonate (1d): White solid; yield 100%. ^{1}H NMR (300 MHz, $CDCl_3$) δ ppm 7.39 (s, 1H), 7.31 (s, 2H), 5.17 (s, 2H), 3.04 (s, 3H).

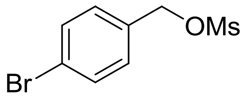

4-Bomobenzyl methanesulfonate (1e): White solid; yield 98%. ^{1}H NMR (300 MHz, $CDCl_3$) δ ppm 7.55 (d, J = 8.4 Hz, 2H), 7.30 (d, J = 8.4 Hz, 2H), 5.19 (s, 2H), 2.95 (s, 3H).

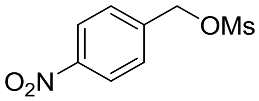

4-Nitrobenzyl methanesulfonate (1f): White solid; yield 97%. ^{1}H NMR (300 MHz, $CDCl_3$) δ ppm 8.29 (d, J = 8.4 Hz, 2H), 7.74 (m, J = 8.4 Hz, 2H), 5.46 (s, 2H), 3.34 (s, 3H).

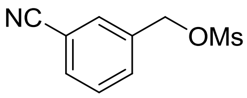

3-Cyanobenzyl methanesulfonate (1g): White solid; yield 95%. ^{1}H NMR (300 MHz, $CDCl_3$) δ ppm 7.52-7.72 (m, 4H), 5.26 (s, 2H), 3.05 (s, 3H).

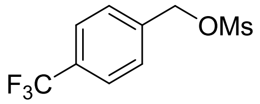

4-(Trifluoromethyl)benzyl methanesulfonate (1h): Colorless oil; yield 100%. ^{1}H NMR (300 MHz, $CDCl_3$) δ ppm 7.68 (d, *J* = 7.2 Hz, 2H), 7.54 (m, *J* = 7.2 Hz, 2H), 5.29 (s, 2H), 3.01 (s, 3H). ^{19}F NMR (282 MHz, $CDCl_3$) δ ppm –62.8 (s, 3F).

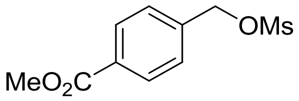

Methyl 4-((methylsulfonyloxy)methyl)benzoate (1i): White solid; yield 98%. ^{1}H NMR (300 MHz, $CDCl_3$) δ ppm 8.08 (d, *J* = 8.4 Hz, 2H), 7.49 (d, *J* = 8.4 Hz, 2H), 5.29 (s, 2H), 2.94 (s, 3H), 2.98 (s, 3H).

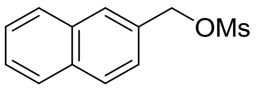

Naphthalen-2-ylmethyl methanesulfonate (1j): White solid; yield 93%. ^{1}H NMR (300 MHz, $CDCl_3$) δ ppm 7.85-7.91 (m, 4H), 7.50-7.55 (m, 3H), 5.41 (s, 2H), 2.91 (s, 3H).

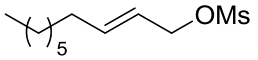

(*E*)-dec-2-enyl methanesulfonate (1k): Colorless oil; yield 100%. ^{1}H NMR (300 MHz, $CDCl_3$) δ ppm 5.88-5.97 (m, 1H), 5.56-5.66 (m, 1H), 4.69 (d, *J* = 6.6 Hz, 2H), 3.01 (s, 3H), 2.06-2.12 (m, 2H), 1.27-1.41 (m, 10H), 0.88 (t, *J* = 6.9 Hz, 3H).

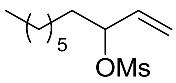

Dec-1-en-3-yl methanesulfonate (1l): Colorless oil; yield 100%. ^{1}H NMR (300 MHz, $CDCl_3$) δ ppm 5.80-5.92 (m, 1H), 5.32-5.44 (m, 2H), 4.97-5.04 (m, 1H), 2.98 (s, 1H), 1.28-1.82 (m, 12H), 0.88 (t, *J* = 6.6 Hz, 3H).

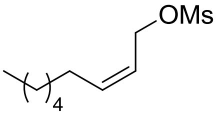

(*Z*)-non-2-enyl methanesulfonate (1m): Colorless oil; yield 100%. ^{1}H NMR (300 MHz, $CDCl_3$) δ ppm 5.76-5.85 (m, 1H), 5.55-5.63 (m, 1H), 4.80 (d, *J* = 6.9 Hz, 2H), 3.01 (s, 3H), 2.10-2.17 (m,

2H), 1.28-1.39 (m, 8H), 0.88 (t, *J* = 6.6 Hz, 3H).

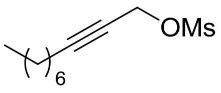

Dec-2-ynyl methanesulfonate (1n): Colorless oil; yield 100%. ^{1}H NMR (300 MHz, $CDCl_3$) δ ppm 4.86 (m, 2H), 3.11 (s, 3H), 2.25 (t, *J* = 6.9 Hz, 2H), 1.28-1.58 (m, 10H), 0.89 (t, *J* = 6.9 Hz, 3H).

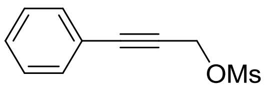

3-Phenylprop-2-ynyl methanesulfonate (1o): Yellow oil; yield 97%. ^{1}H NMR (300 MHz, $CDCl_3$) δ ppm 7.35-7.48 (m, 5H), 5.09 (s, 2H), 3.17 (s, 3H).

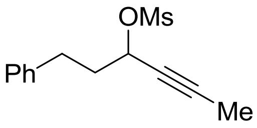

1-Phenylhex-4-yn-3-yl methanesulfonate (1p): Colorless oil; yield 83%. ^{1}H NMR (300 MHz, $CDCl_3$) δ ppm 7.19-7.32 (m, 5H), 5.12-5.17 (m, 1H), 3.11 (s, 3H), 2.78-2.84 (m, 2H), 2.12-2.22 (m, 2H), 1.91 (d, *J* = 1.8 Hz, 3H).

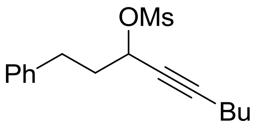

1-Phenylnon-4-yn-3-yl methanesulfonate (1q): Colorless oil; yield 93%. ^{1}H NMR (300 MHz, $CDCl_3$) δ ppm 7.19-7.32 (m, 5H), 5.16 (t, *J* = 6.6 Hz, 1H), 3.11 (s, 1H), 2.81-2.82 (m, 2H), 2.18-2.29 (m, 4H), 1.42-1.52 (m, 4H), 0.92 (t, *J* = 6.9 Hz, 3H).

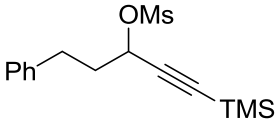

5-Phenyl-1-(trimethylsilyl)pent-1-yn-3-yl methanesulfonate (1r): Colorless oil; yield 100%. ^{1}H NMR (300 MHz, $CDCl_3$) δ ppm 7.19-7.32 (m, 5H), 5.13 (t, *J* = 6.6 Hz, 1H), 3.14 (s, 3H), 2.78-2.85 (m, 2H), 2.14-2.25 (m, 2H), 0.20 (s, 9H).

General procedure for the Cu-mediated trifluoromethylation of benzyl methanesulfonates:

Into a Schlenk tube equipped with a magnetic stir bar were added CuI (2.2 mmol) and KF (4.0 mmol) under Ar atmosphere. DMF (5.0 mL) and Me_3SiCF_3 (2.0 equiv) were added. After stirring for 20 minutes, the mixture was heated to 60 °C and then the benzyl methanesulfonate (2.0 mmol) was added under N_2 atmosphere. The reaction mixture was kept at 60 °C for 4 hours and then allowed to cool down to room temperature. The resulting mixture was diluted with diethyl ether, washed with water and brine, dried over sodium sulfate, and concentrated. The crude products were purified by column chromatography on silica gel to give the products.

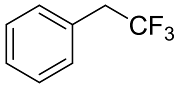

(2,2,2-Trifluoroethyl)benzene (2a): Colorless liquid; yield 76% after distillation on 10 mmol scale. ^{1}H NMR (300 MHz, $CDCl_3$) δ ppm 7.27-7.32 (m, 5H), 3.31 (q, *J* = 10.8 Hz, 2H). ^{19}F NMR (282 MHz, $CDCl_3$) δ ppm -66.0 (t, *J* = 11.6 Hz, 3F). ^{13}C NMR (100 MHz, $CDCl_3$) δ ppm 130.2, 128.7, 128.1, 125.9 (q, *J* = 275.5 Hz), 40.2 (q, *J* = 29.6 Hz). HRMS (EI) Calculated for $C_8H_7F_3$:160.0500; Found: 160.0502.

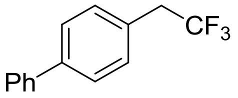

4-(2,2,2-Trifluoroethyl)biphenyl (2b): White solid; yield 79%; mp 81-82 ^{o}C. ^{1}H NMR (300 MHz, $CDCl_3$) δ ppm 7.36-7.60 (m, 9H), 3.41 (q, *J* = 10.5 Hz, 2H). ^{19}F NMR (282 MHz, $CDCl_3$) δ ppm -65.8 (t, *J* = 11.3 Hz, 3F). ^{13}C NMR (100 MHz, $CDCl_3$) δ ppm 141.1, 140.5, 130.6, 129.2, 128.9, 127.6, 127.4, 127.1, 125.9 (q, *J* = 275.6 Hz), 39.9 (q, *J* = 29.6 Hz). Anal. Calcd. for $C_{14}H_{11}F_3$: C, 71.18; H, 4.69. Found: C, 71.17; H, 4.69.

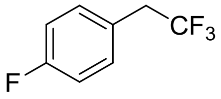

1-Fluoro-4-(2,2,2-trifluoroethyl)benzene (2c): Colorless liquid; yield 75% after distillation on 10 mmol scale. ^{1}H NMR (300 MHz, $CDCl_3$) δ ppm 7.22-7.27 (m, 2H), 7.00-7.05 (m, 2H), 3.32 (q, *J* = 10.8 Hz, 2H). ^{19}F NMR (282 MHz, $CDCl_3$) δ ppm -66.4 (t, *J* = 11.3 Hz, 3F), -114.2 (s, 1F). ^{13}C

NMR (100 MHz, $CDCl_3$) δ ppm 162.9 (d, *J* = 245.9 Hz), 132.0 (d, *J* = 8.4 Hz),126.1, 125.8 (q, *J* = 275.5 Hz), 115.8 (d, *J* = 22.0 Hz), 39.6 (q, *J* = 29.6 Hz). HRMS (EI) Calculated for $C_8H_6F_4$: 178.0406; Found: 178.0403.

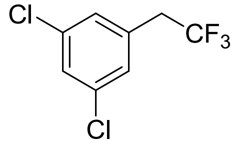

1,3-Dichloro-5-(2,2,2-trifluoroethyl)benzene (2d): White solid; yield 72%; mp 46-47 ^{o}C. ^{1}H NMR (300 MHz, $CDCl_3$) δ ppm 7.36 (s, 1H), 7.20 (s, 2H), 3.33 (q, *J* = 10.5 Hz, 2H). ^{19}F NMR (282 MHz, $CDCl_3$) δ ppm -65.7 (t, *J* = 10.0 Hz, 3F). ^{13}C NMR (100 MHz, $CDCl_3$) δ ppm 135.1, 133.3, 128.8, 128.7, 125.3 (q, *J* = 276.1 Hz), 39.7 (q, *J* = 30.0 Hz). Anal. Calcd. for $C_8H_5Cl_2F_3$: C, 49.95; H, 2.20. Found: C, 41.89; H, 2.21.

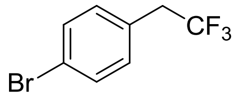

1-Bromo-4-(2,2,2-trifluoroethyl)benzene (2e): White solid; yield 78%; mp 55-57 ^{o}C. ^{1}H NMR (300 MHz, $CDCl_3$) δ ppm 7.48 (d, *J* = 6.6 Hz, 2H), 7.15 (d, *J* = 7.8 Hz, 2H), 3.30 (q, *J* = 10.5 Hz, 2H). ^{19}F NMR (282 MHz, $CDCl_3$) δ ppm -66.0 (t, *J* = 10.2 Hz, 3F). ^{13}C NMR (100 MHz, $CDCl_3$) δ ppm 132.0, 131.9, 129.2, 125.6 (q, *J* = 274.8 Hz), 39.8 (q, *J* = 29.6 Hz). HRMS (EI) Calculated for $C_8H_6BrF_3$: 237.9605; Found: 237.9607.

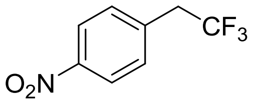

1-Nitro-4-(2,2,2-trifluoroethyl)benzene (2f): White solid; yield 40%; mp 65-66 ^{o}C. ^{1}H NMR (300 MHz, $CDCl_3$) δ ppm 8.24 (d, *J* = 8.4 Hz, 2H), 7.50 (d, *J* = 8.4 Hz, 2H), 3.50 (q, *J* = 10.4 Hz, 2H). ^{19}F NMR (282 MHz, $CDCl_3$) δ ppm -65.5 (t, *J* = 10.9 Hz, 3F). ^{13}C NMR (100 MHz, $CDCl_3$) δ ppm 148.1, 137.4, 131.3, 125.2 (q, *J* = 276.6 Hz), 124.0, 40.1 (q, *J* = 30.6 Hz). HRMS (EI) Calculated for $C_8H_6F_3NO_2$: 205.0351; Found: 205.0347.

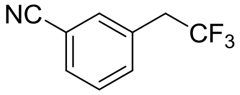

3-(2,2,2-Trifluoroethyl)benzonitrile (2g): White solid; yield 81%; mp 65-67 ^{o}C. ^{1}H NMR (300

MHz, $CDCl_3$) δ ppm 7.47-7.66 (m, 4H), 3.43 (q, *J* = 10.8 Hz, 2H). ^{19}F NMR (282 MHz, $CDCl_3$) δ ppm -65.9 (t, *J* = 10.2 Hz, 3F). ^{13}C NMR (100 MHz, $CDCl_3$) δ ppm 134.6, 133.6, 131.8, 125.2 (q, *J* = 275.5 Hz), 118.2, 113.0, 39.7 (q, *J* = 30.4 Hz). HRMS (EI) Calculated for $C_9H_6F_3N$: 185.0452; Found: 185.0452.

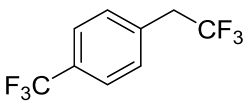

1-(2,2,2-Trifluoroethyl)-4-(trifluoromethyl)benzene (2h): Colorless liquid; yield 80%. ^{1}H NMR (300 MHz, $CDCl_3$) δ ppm 7.63 (d, *J* = 8.1 Hz, 2H), 7.43 (d, *J* = 7.8 Hz, 2H), 3.44 (q, *J* = 10.5 Hz, 2H). ^{19}F NMR (282 MHz, $CDCl_3$) δ ppm -62.7 (s, 3F), -65.7 (t, *J* = 11.3 Hz, 3F). ^{13}C NMR (100 MHz, $CDCl_3$) δ ppm 134.3, 130.7, 129.0, 125.8 (q, *J* = 4.2 Hz), 125.6 (q, *J* = 275.3 Hz), 124.2 (q, *J* = 270.8 Hz), 40.2 (q, *J* = 30.1 Hz). HRMS (EI) Calculated for $C_9H_6F_6$: 228.0374; Found: 228.0375.

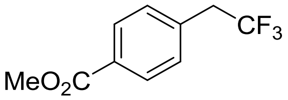

Methyl 4-(2,2,2-trifluoroethyl)benzoate (2i): White solid; yield 68%; mp 54-55 °C. ^{1}H NMR (300 MHz, $CDCl_3$) δ ppm 8.03 (d, *J* = 8.7 Hz, 2H), 7.38 (d, *J* = 8.1 Hz, 2H), 3.92 (s, 2H), 3.43 (q, *J* = 10.5 Hz, 2H). ^{19}F NMR (282 MHz, $CDCl_3$) δ ppm -65.6 (t, *J* = 10.7 Hz, 3F). ^{13}C NMR (100 MHz, $CDCl_3$) δ ppm 166.7, 135.2, 130.3, 130.2, 130.0, 125.6 (q, *J* = 275.7 Hz), 52.2, 40.2 (q, *J* = 29.3 Hz).

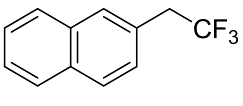

2-(2,2,2-Trifluoroethyl)naphthalene (2j): White solid; yield 78%; mp 54-55 °C. ^{1}H NMR (300 MHz, $CDCl_3$) δ ppm 7.39-7.85 (m, 7H), 3.53 (q, *J* = 11.1 Hz, 2H). ^{19}F NMR (282 MHz, $CDCl_3$) δ ppm -65.6 (t, *J* = 11.3 Hz, 3F). ^{13}C NMR (100 MHz, $CDCl_3$) δ ppm 133.4, 133.0, 129.6, 128.5, 127.9, 127.8, 127.7, 126.5, 126.4, 126.1 (q, *J* = 275.6 Hz), 40.4 (q, *J* = 29.8 Hz). Anal. Calcd. for $C_{12}H_9F_3$: C, 68.57; H, 4.32. Found: C, 68.57; H, 4.36.

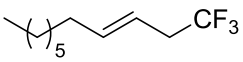

(*E*)-1,1,1-Trifluoroundec-3-ene (2k): Colorless liquid; yield 78% from **1k**, 80% from **1l**. ^{1}H NMR (300 MHz, $CDCl_3$) δ ppm 5.65-5.73 (m, 1H), 5.33-5.40 (m, 1H), 2.70-2.79 (m, 2H), 2.02-2.07 (m, 2H), 1.28-1.39 (m, 10H), 0.88 (t, *J* = 6.8 Hz, 3H). ^{19}F NMR (282 MHz, $CDCl_3$) δ ppm -67.0 (t, *J* = 9.4 Hz, 3F). ^{13}C NMR (100 MHz, $CDCl_3$) δ ppm 138.7, 126.3 (q, *J* = 274.9 Hz), 117.7 (q, *J* = 4.3 Hz), 37.6 (q, *J* = 29.0 Hz), 32.7, 32.1, 29.4, 29.3, 29.2, 22.9, 14.2. HRMS (EI) Calculated for $C_{11}H_{19}F_3$: 208.1439; Found: 208.1438.

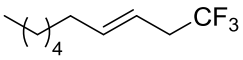

(*E*)-1,1,1-Trifluorodec-3-ene (2m): Colorless liquid; yield 87%. ^{1}H NMR (300 MHz, $CDCl_3$) δ ppm 5.66-5.73 (m, 1H), 5.33-5.40 (m, 1H), 2.71-2.81 (m, 2H), 2.02-2.07 (m, 2H), 1.28-1.39 (m, 8H), 0.88 (t, *J* = 4.8 Hz, 3H). ^{19}F NMR (282 MHz, $CDCl_3$) δ ppm -66.8 (t, *J* = 8.2 Hz, 3F). ^{13}C NMR (100 MHz, $CDCl_3$) δ ppm 138.7, 126.3 (q, *J* = 275.0 Hz), 117.7 (q, *J* = 3.6 Hz), 37.6 (q, *J* = 28.6 Hz), 32.7, 31.9, 29.1, 28.9, 22.8, 14.2. IR (neat) 2960, 2930, 1367, 1253, 1138 cm^{-1}. HRMS (EI) Calculated for $C_{10}H_{17}F_3$: 194.1282; Found: 194.1279.

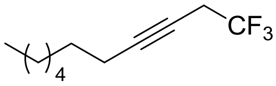

1,1,1-Trifluoroundec-3-yne (2n): Colorless liquid; yield 64%. ^{1}H NMR (300 MHz, $CDCl_3$) δ ppm 2.98 (q, *J* = 7.5 Hz, 2H), 2.16 (t, *J* = 5.4 Hz, 2H), 1.28-1.54 (m, 10H), 0.88 (t, *J* = 5.1 Hz, 3H). ^{19}F NMR (282 MHz, $CDCl_3$) δ ppm -67.3 (t, *J* = 9.8 Hz, 3F). ^{13}C NMR (100 MHz, $CDCl_3$) δ ppm 124.7 (q, *J* = 274.2 Hz), 85.2, 68.3 (q, *J* = 4.8 Hz), 31.9, 28.94, 28.89, 28.6, 26.3 (q, *J* = 33.7 Hz), 22.8, 18.7, 14.2. IR (neat) 2933, 2859, 2245, 1366, 1258, 1113 cm^{-1}. Anal. Calcd. for $C_{11}H_{17}F_3$: C, 64.06; H, 8.31. Found: C, 64.09; H, 8.32.

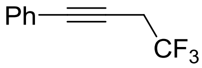

(4,4,4-Trifluorobut-1-ynyl)benzene (2o): Yellow liquid; yield 50%. ^{1}H NMR (300 MHz, $CDCl_3$) δ ppm 7.46-7.31 (m, 5H), 3.27 (q, *J* = 9.6 Hz, 2H). ^{19}F NMR (282 MHz, $CDCl_3$) δ ppm -66.0 (t, *J* = 11.6 Hz, 3F). ^{13}C NMR (100 MHz, $CDCl_3$) δ ppm 131.0, 128.9, 128.5, 124.4 (q, *J* = 275.1 Hz), 122.3, 84.5, 77.5 (q, J = 4.1 Hz), 26.9 (q, *J* = 35.1 Hz). HRMS (EI) Calculated for $C_{10}H_7F_3$: 184.0500; Found: 184.0498.

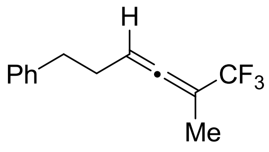

(6,6,6-Trifluoro-5-methylhexa-3,4-dienyl)benzene (2p): Colorless liquid; yield 89%. ^{1}H NMR (300 MHz, $CDCl_3$) δ ppm 7.17-7.31 (m, 5H), 5.56 (m, 1H), 2.74 (t, J = 7.5 Hz, 2H), 2.37-2.44 (m, 2H), 1.73 (d, J = 2.7 Hz, 3H). ^{19}F NMR (282 MHz, $CDCl_3$) δ ppm -65.8 (d, J = 4.2 Hz, 3F). ^{13}C NMR (100 MHz, $CDCl_3$) δ ppm 202.7, 141.1, 128.6, 128.5, 126.2, 124.1 (q, J = 271.9 Hz), 96.5, 94.3 (q, J = 34.3 Hz), 34.9, 29.7, 13.0. IR (neat) 3065, 3029, 2932, 1981, 1455, 1307, 1118 cm^{-1}. Anal. Calcd. for $C_{13}H_{13}F_3$: C, 69.02; H,5.79. Found: C, 69.00; H, 5.72.

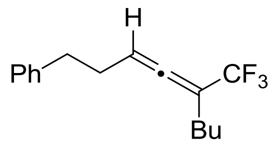

(5-(Trifluoromethyl)nona-3,4-dienyl)benzene (2q): Colorless liquid; yield 93%. ^{1}H NMR (300 MHz, $CDCl_3$) δ ppm 7.17-7.31 (m, 5H), 5.60-5.65 (m, 1H), 2.74 (t, J = 7.8 Hz, 2H), 2.37-2.46 (m, 2H), 2.05 (m, 2H), 1.31-1.34 (m, 4H), 0.89 (t, J = 7.2 Hz, 3H). ^{19}F NMR (282 MHz, $CDCl_3$) δ ppm -64.3 (d, J = 3.1 Hz, 3F). ^{13}C NMR (100 MHz, $CDCl_3$) δ ppm 202.4, 141.1, 128.6, 128.5, 126.2, 124.2 (q, J = 273.2 Hz), 99.5 (q, J = 34.3 Hz), 97.9, 35.1, 29.9, 29.5, 26.0, 22.2, 13.9. IR (neat) 3066, 2959, 2864, 1976, 1455, 1298, 1118 cm^{-1}. HRMS (EI) Calculated for $C_{16}H_{19}F_3$: 268.1439; Found: 268.1440.

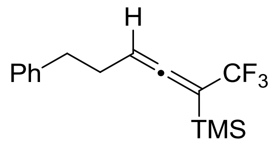

Trimethyl(1,1,1-trifluoro-6-phenylhexa-2,3-dien-2-yl)silane (2r): Colorless liquid; yield 70%. ^{1}H NMR (300 MHz, $CDCl_3$) δ ppm 7.18-7.32 (m, 5H), 5.34-5.38 (m, 1H), 2.73 (t, J = 7.8 Hz, 2H), 2.36-2.43 (m, 2H), 0.16 (s, 9H). ^{19}F NMR (282 MHz, $CDCl_3$) δ ppm -55.8 (d, J = 4.2 Hz, 3F). ^{13}C NMR (100 MHz, $CDCl_3$) δ ppm 208.7, 141.2, 128.63, 128.58, 126.3, 125.2 (q, J = 271.6 Hz), 94.4 (q, J = 34.7 Hz), 90.9, 35.5, 29.2, -1.2. IR (neat) 3066, 2960, 1956, 1269, 1113 cm^{-1}. Anal. Calcd. for $C_{15}H_{19}F_3Si$: C, 63.35; H, 6.73. Found: C, 62.98; H, 6.82.

References

1. Akai, S.; Hanada, R.; Fujiwara, N.; Kita, Y.; Egi, M. *Org. Lett.* **2010**, *12*, 4900-4903.

2. Shibuya, G. M.; Kanady, J. S.; Vanderwal, C. D. *J. Am. Chem. Soc.* **2008**, *130*, 12514-12518.

3. Li, D.; Tanaka, T.; Ohmiya, H.; Sawamura, M. *Org. Lett.* **2010**, *12*, 3344-3347.

4. Liang, A.; Li, X.; Liu, D.; Li, J.; Zou, D.; Wu, Y.; Wu, Y. *Chem. Commun.* **2012**, *48*, 8273-8275.

5. Zhao, Y.; Hu, J. *Angew. Chem. Int. Ed.* **2012**, *51*, 1033-1036.

6. Zhu, L.; Li, Y.; Zhao, Y.; Hu, J. *Tetrahedron Lett.* **2010**, *51*, 6150-6152.

7. Kawai, H.; Furukawa, T.; Nomura, Y.; Tokunaga, E.; Shibata, N. *Org. Lett.* **2011**, *13*, 3596-3599.

8. Jeong, I. H.; Min, Y. K.; Kim, Y. S.; Kim, B. T.; Cho, K. Y. *Bull. Korean Chem. Soc.* **1994**, *15*, 519.

9. Zhao, T. S. N.; Szabó, K. J. *Org. Lett.* **2012**, *14*, 3966-3969.

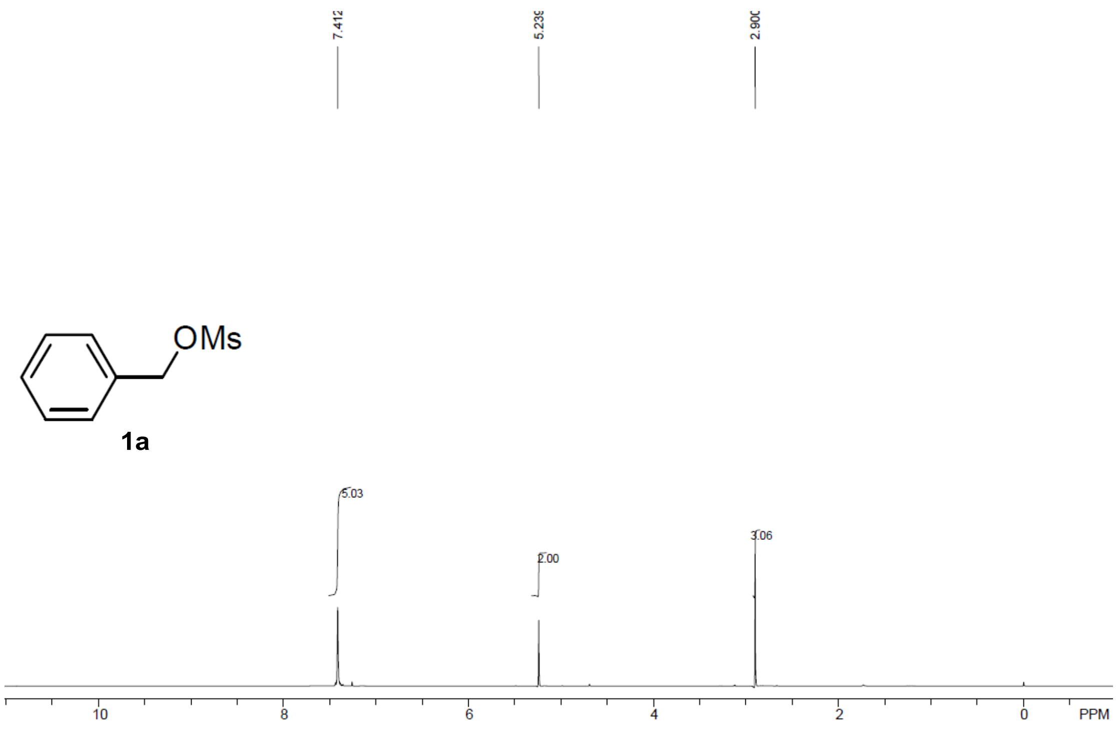
7.412
5.239
2.900
OMs
5.03
2.00
3.06
10
8
6
4
2
0
PPM

1a

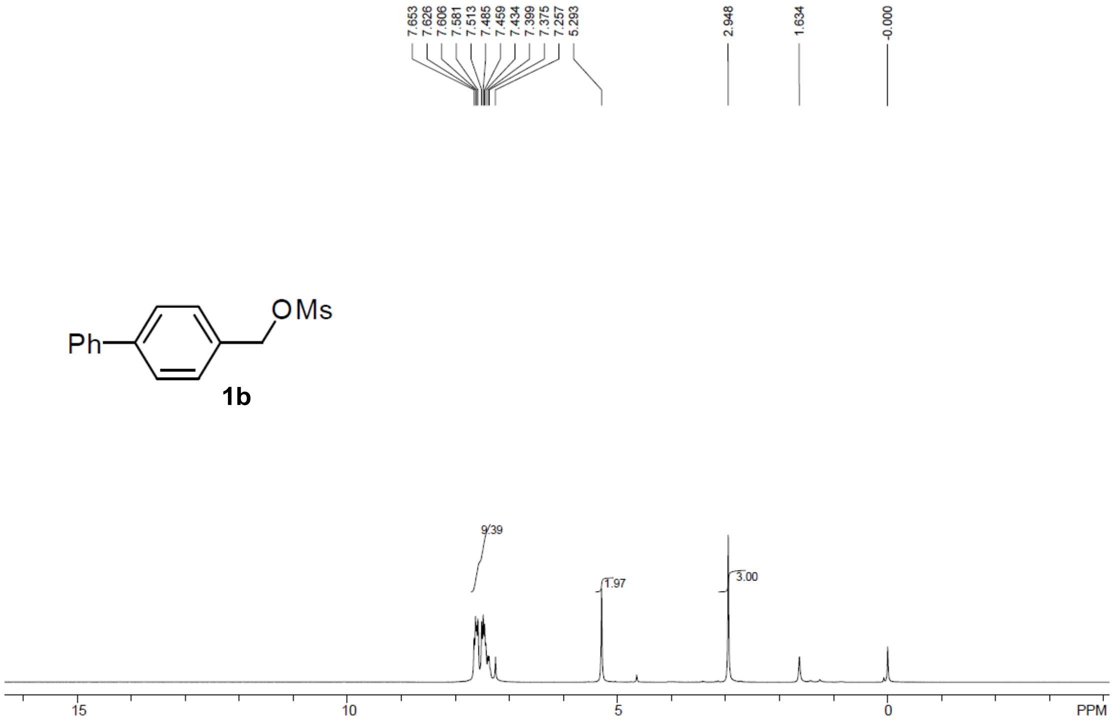
7.653
7.626
7.606
7.581
7.513
7.485
7.459
7.434
7.399
7.375
7.257
5.293
2.948
1.634
-0.000
Ph
OMs
9.39
1.97
3.00
15
10
5
0
PPM

1b

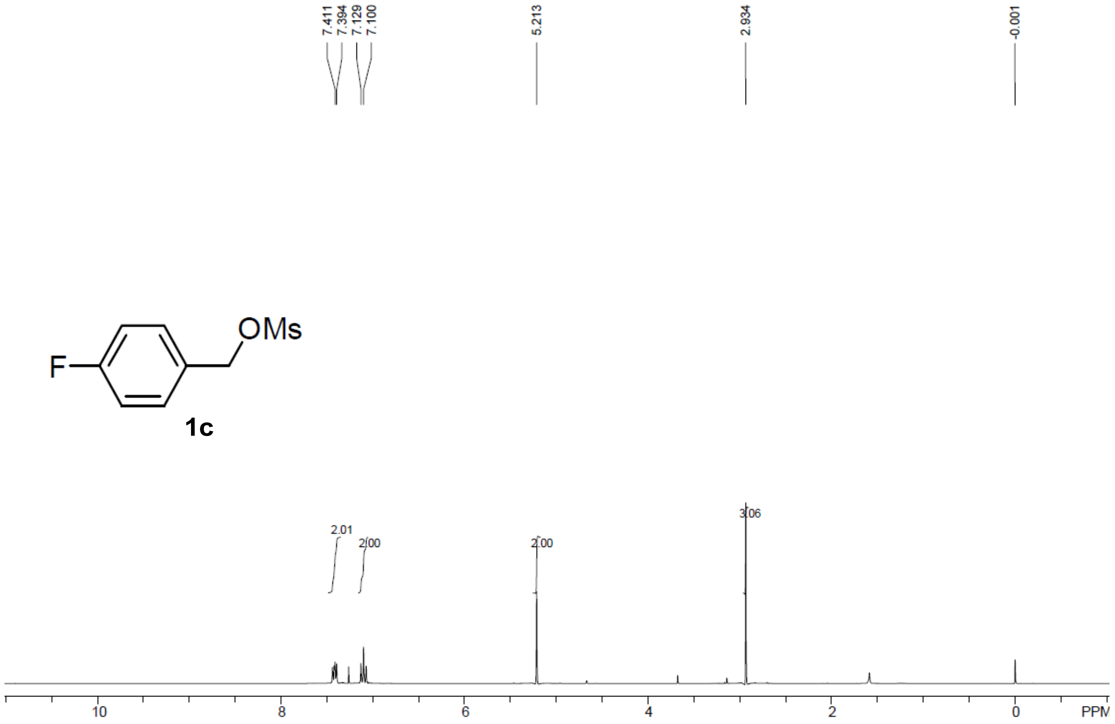
7.411
7.394
7.129
7.100
5.213
2.934
-0.001
OMs
F
1c
2.01
2.00
2.00
3.06
10
8
6
4
2
0
PPM

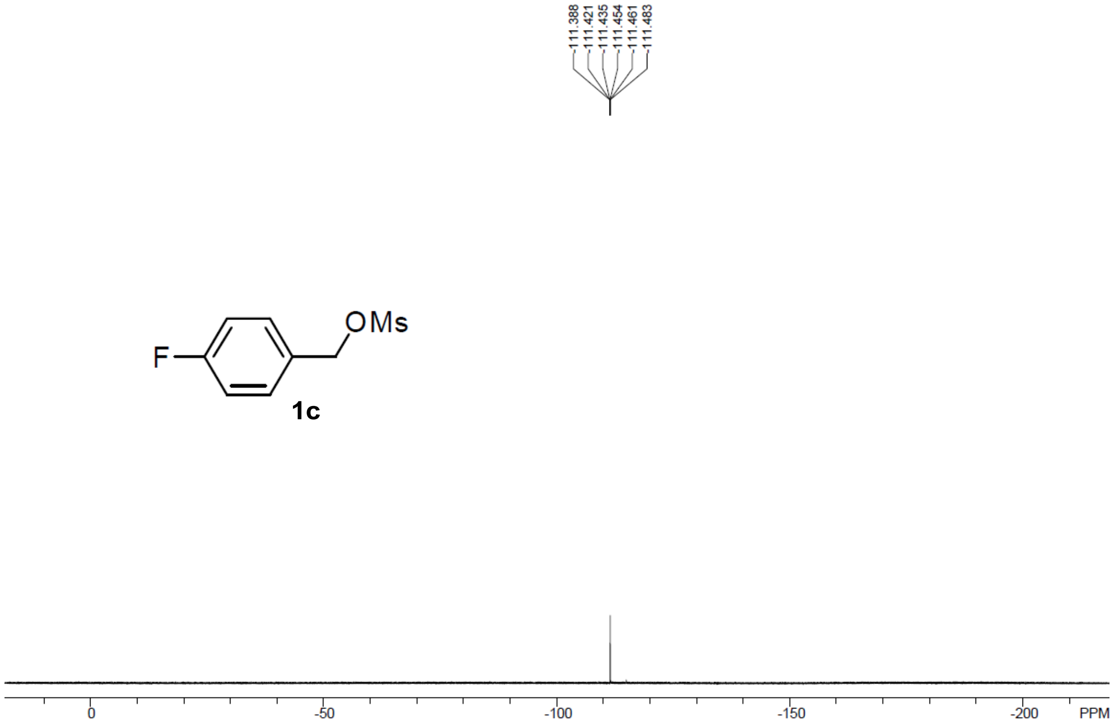
-111.388
-111.421
-111.435
-111.454
-111.461
-111.483
OMs
F
1c
0
-50
-100
-150
-200
PPM

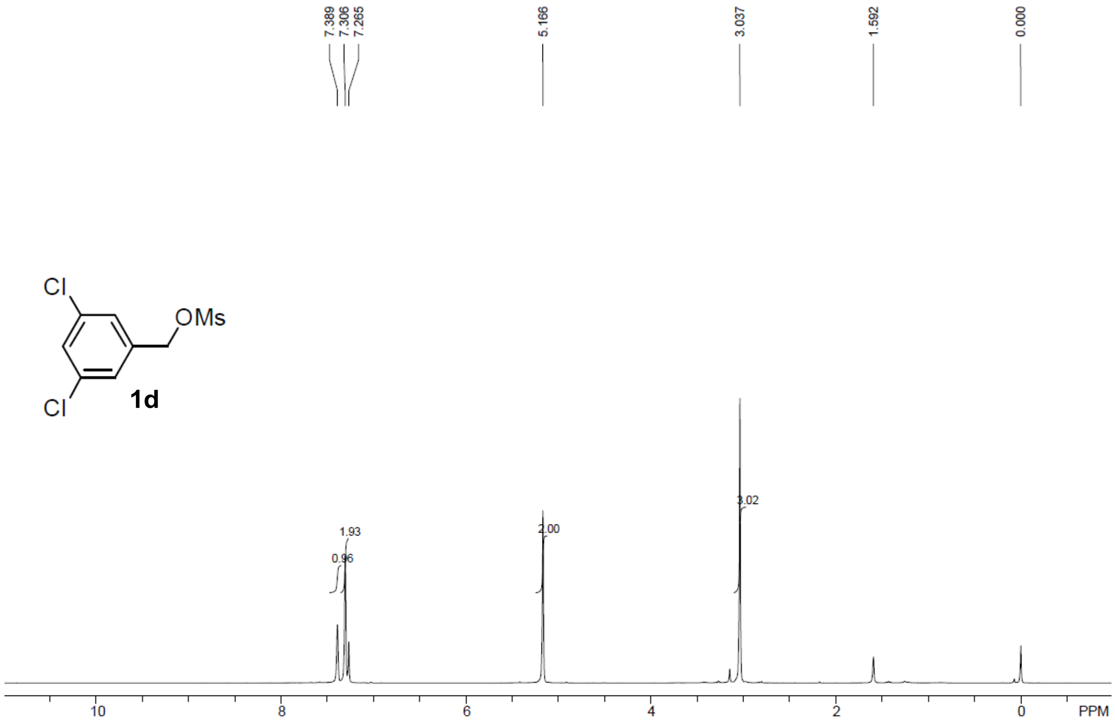
7.389
7.306
7.265
5.166
3.037
1.592
0.000
Cl
OMs
Cl
1d
1.93
0.96
2.00
3.02
10
8
6
4
2
0
PPM

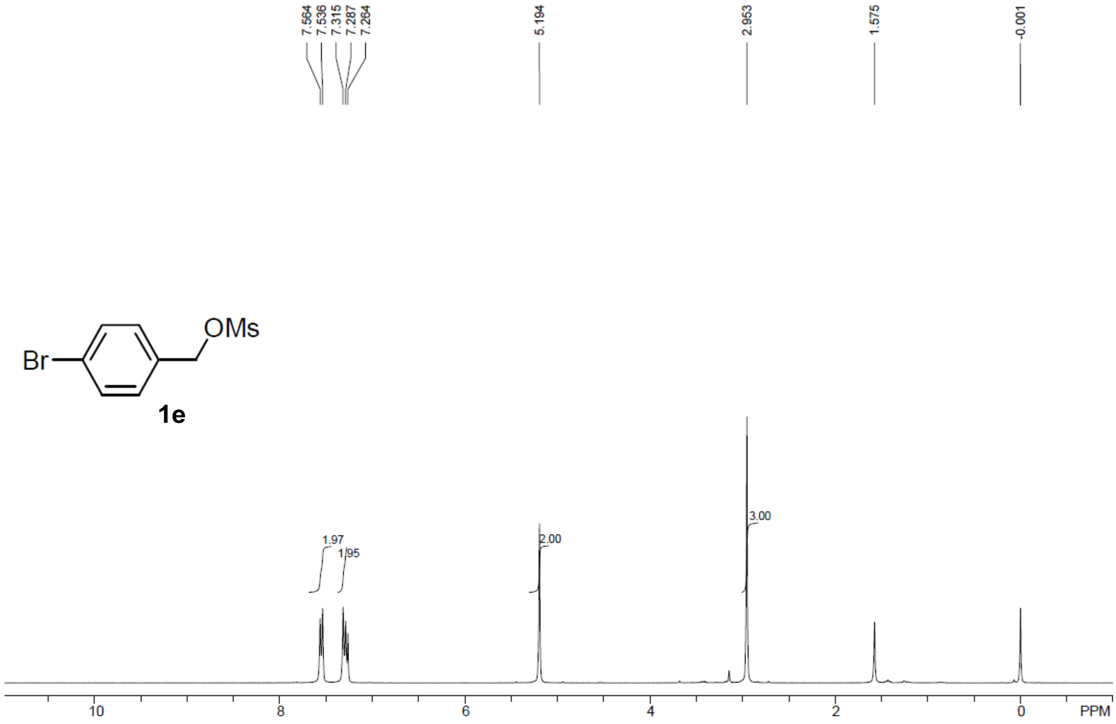
7.564
7.536
7.315
7.287
7.264
5.194
2.953
1.575
-0.001
Br
OMs
1e
1.97
1.95
2.00
3.00
10
8
6
4
2
0
PPM

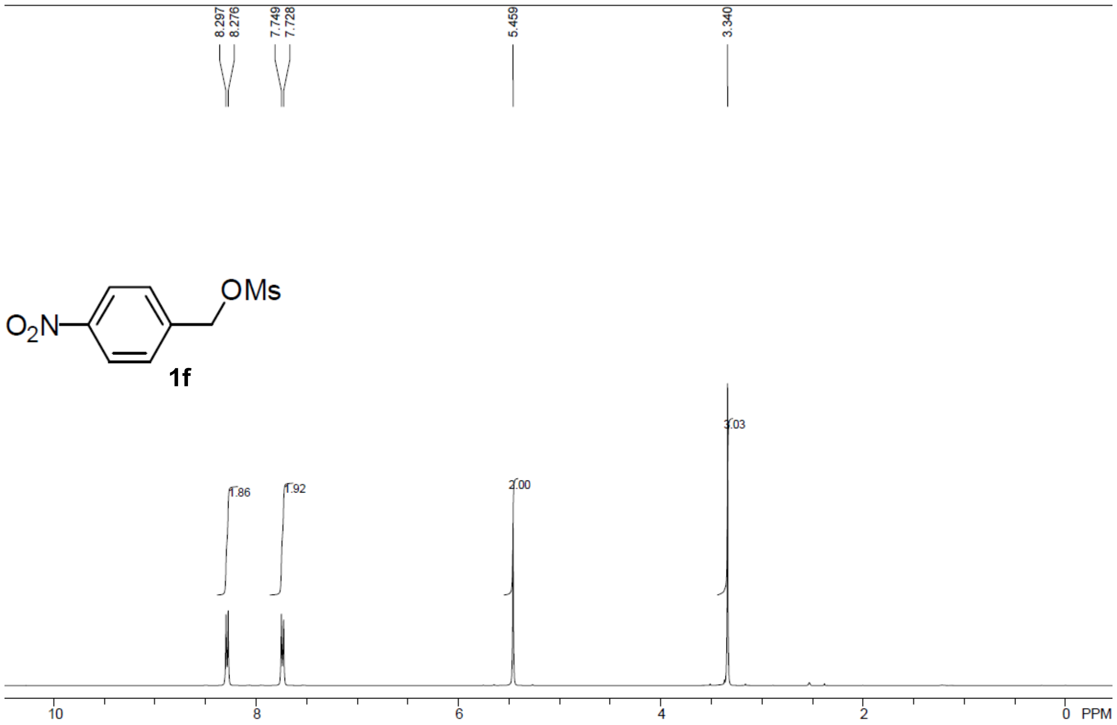
8.297
8.276
7.749
7.728
5.459
3.340
O2N
OMs
1f
1.86
1.92
2.00
3.03
10
8
6
4
2
0 PPM

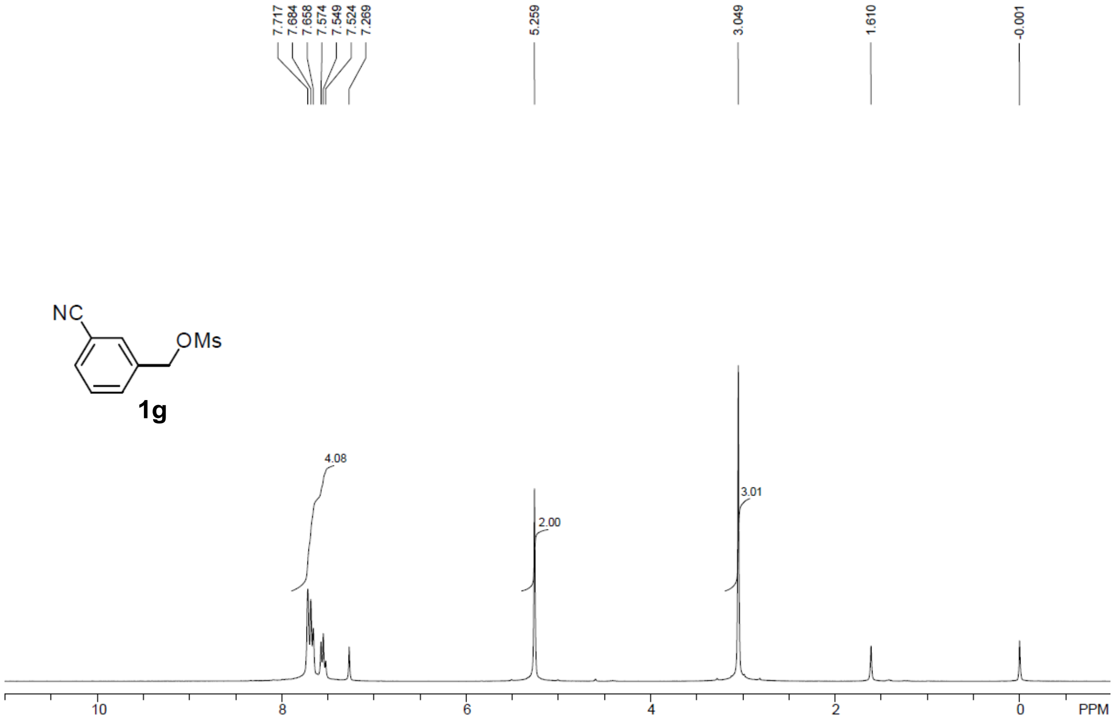
7.717
7.684
7.658
7.574
7.549
7.524
7.269
5.259
3.049
1.610
-0.001
NC
OMs
1g
4.08
2.00
3.01
10
8
6
4
2
0
PPM

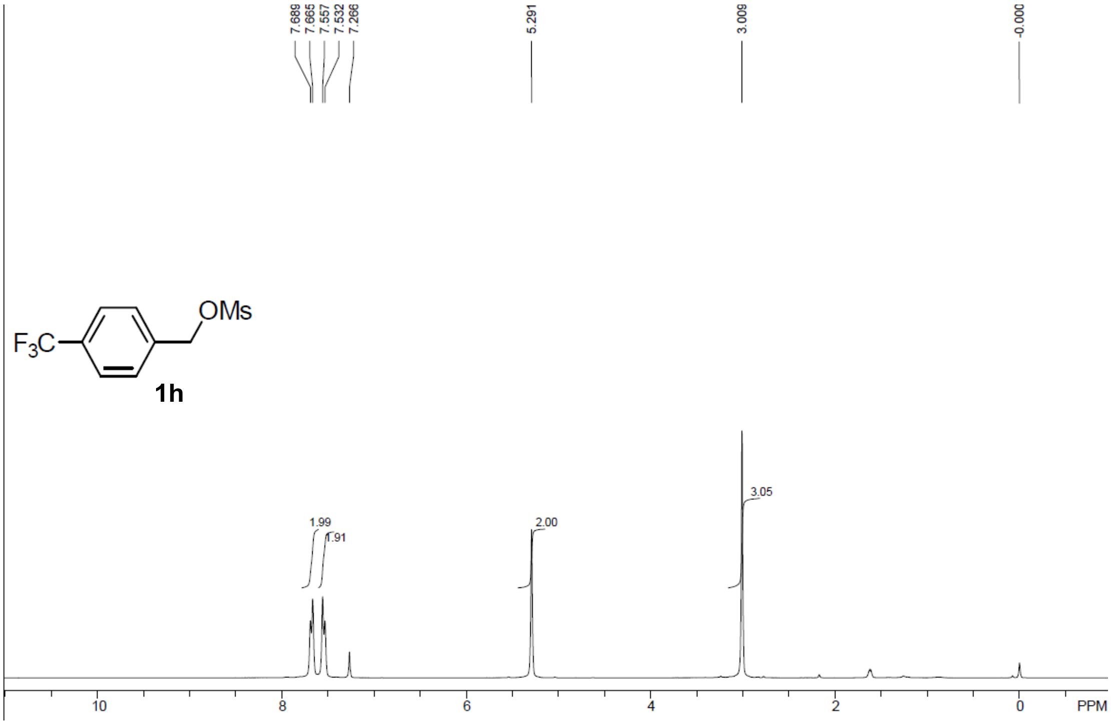
7.689
7.665
7.557
7.532
7.266
5.291
3.009
-0.000
F3C
OMs
1h
1.99
1.91
2.00
3.05
10
8
6
4
2
0
PPM

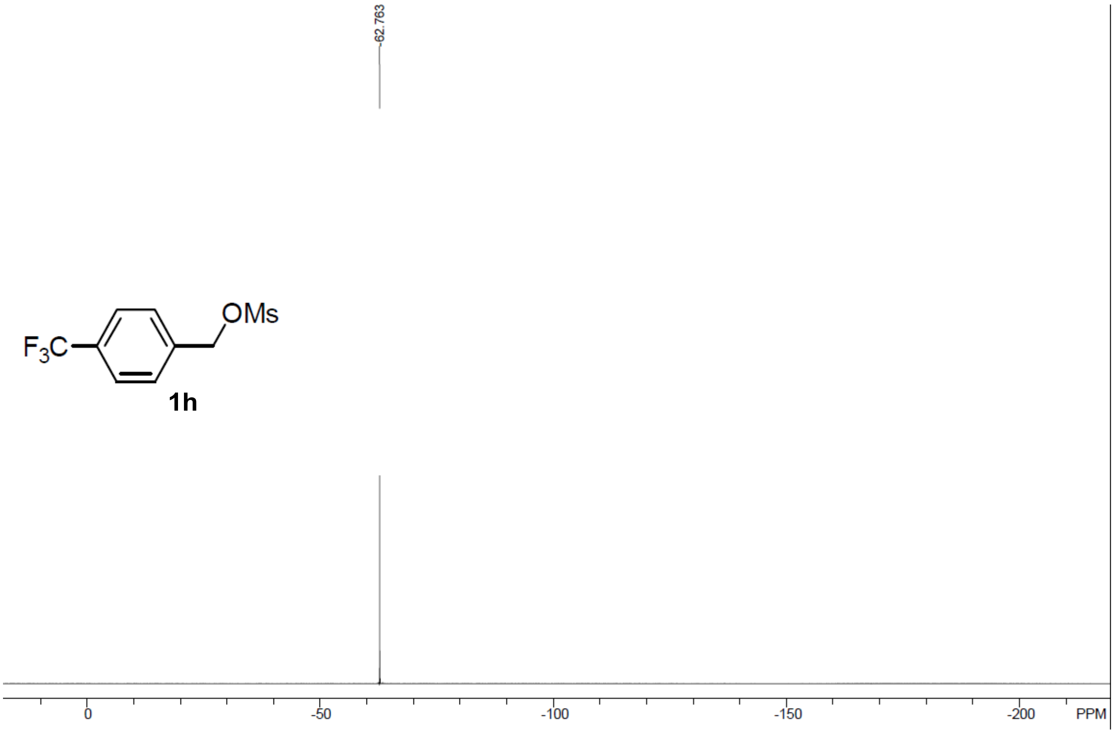
-62.763
F3C
OMs
1h
0
-50
-100
-150
-200
PPM

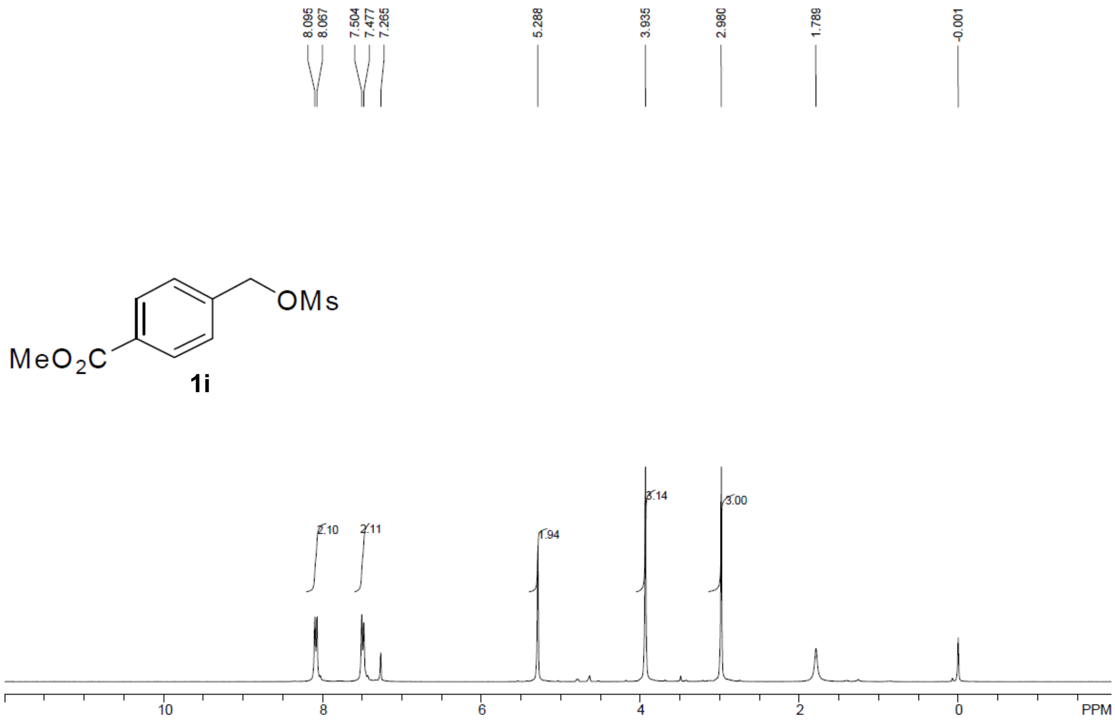
8.095
8.067
7.504
7.477
7.265
5.288
3.935
2.980
1.789
-0.001
MeO2C
OMs
1i
2.10
2.11
1.94
3.14
3.00
PPM

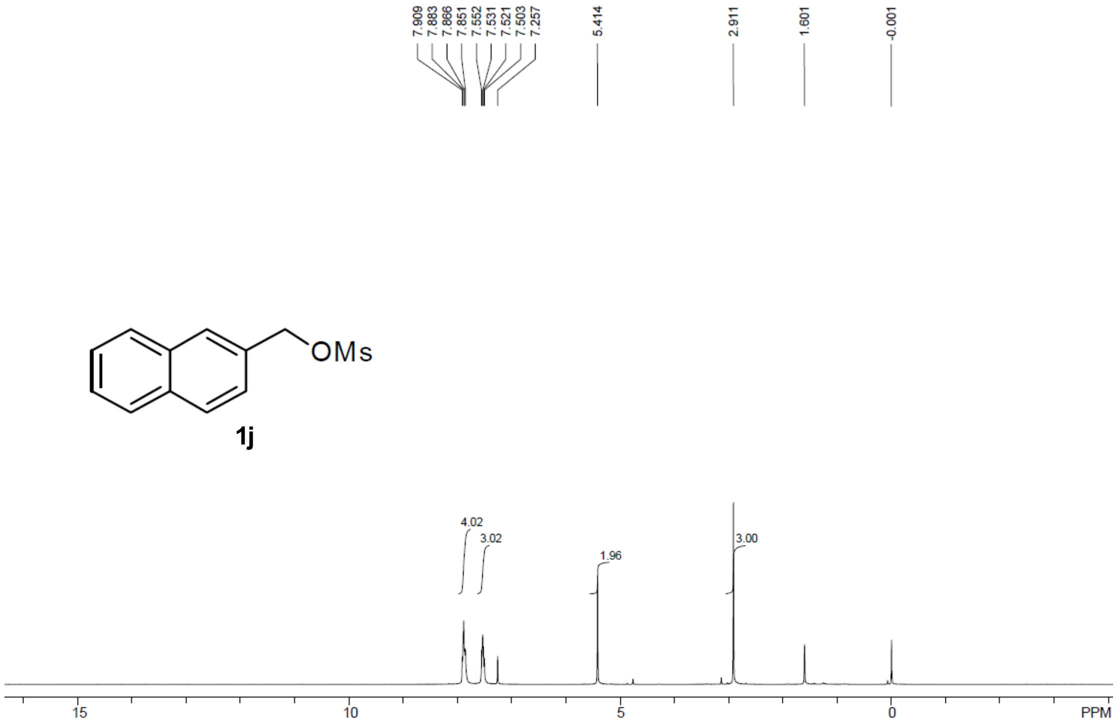
7.909
7.883
7.866
7.851
7.552
7.531
7.521
7.503
7.257
5.414
2.911
1.601
-0.001
OMs
1j
4.02
3.02
1.96
3.00
PPM

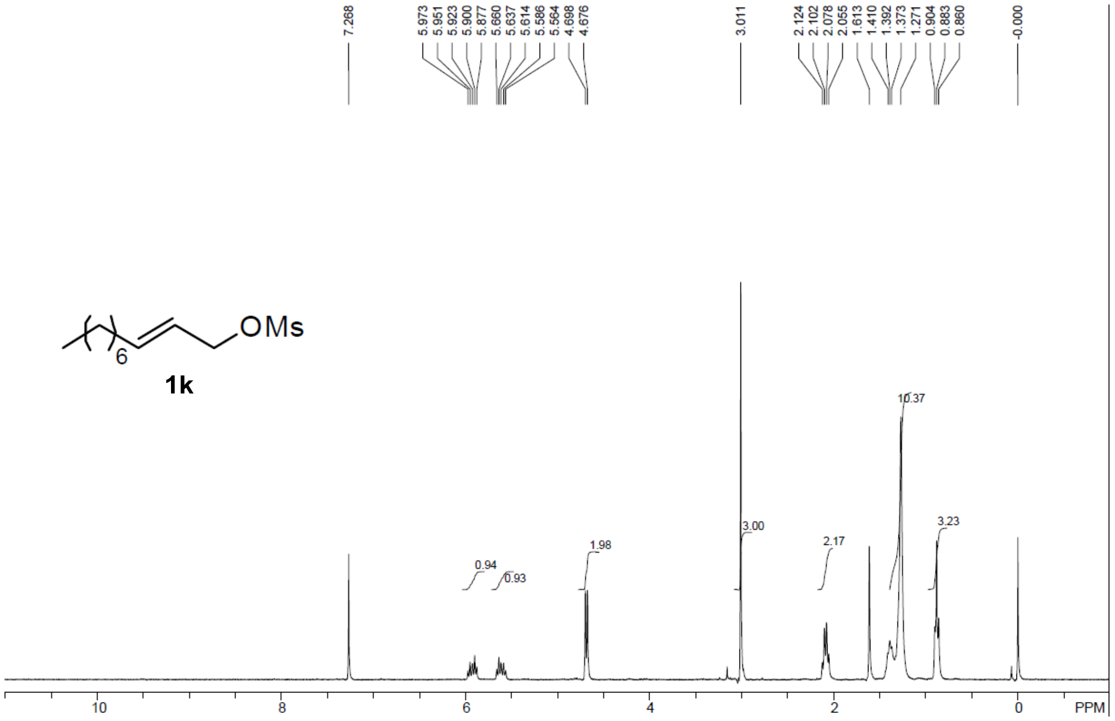
OMs
1k
PPM

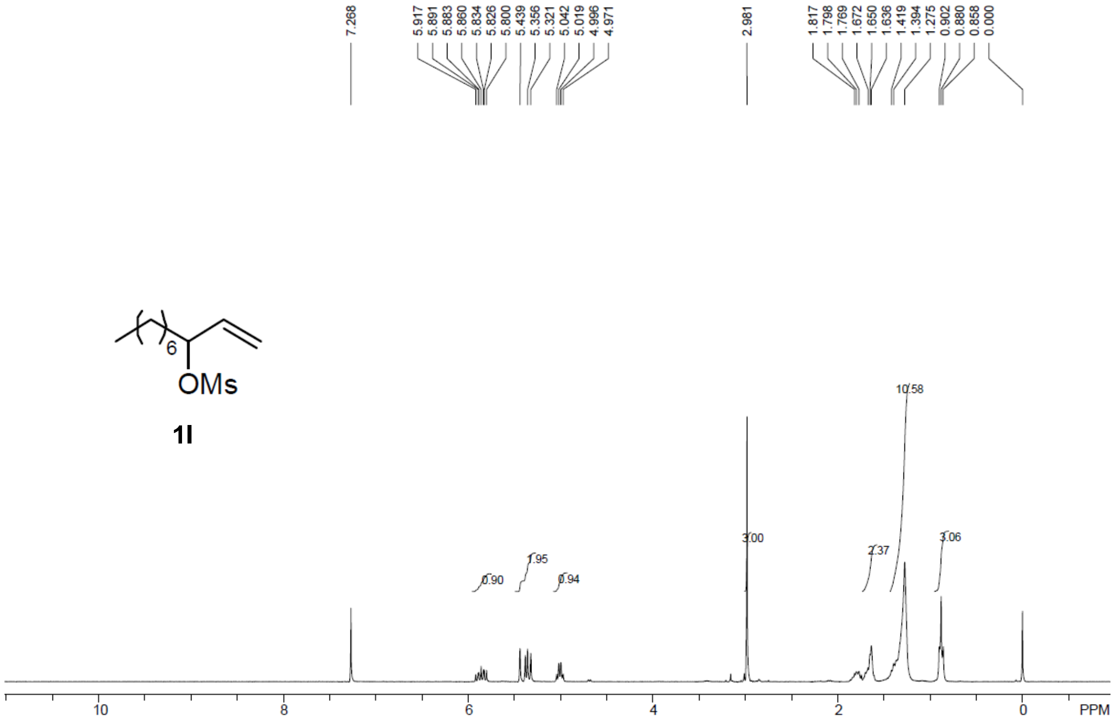
OMs
1l
PPM

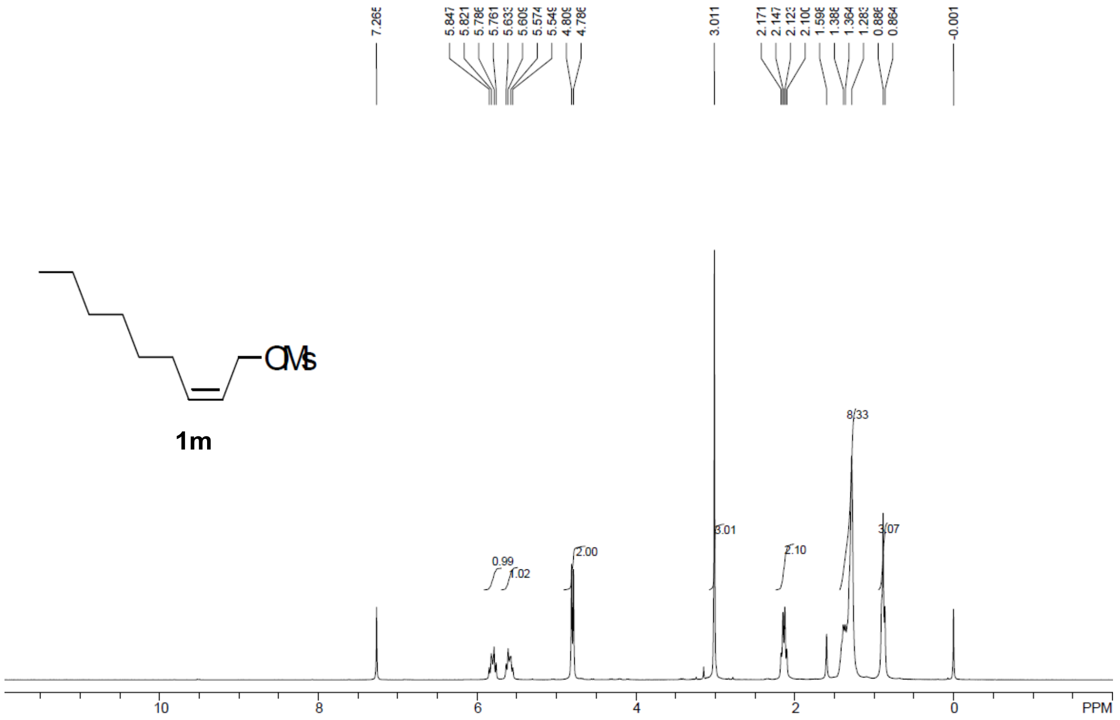
7.265
5.847
5.821
5.786
5.761
5.633
5.609
5.574
5.549
4.809
4.786
3.011
2.171
2.147
2.123
2.100
1.598
1.388
1.364
1.283
0.888
0.864
-0.001
OMs
1m
0.99
1.02
2.00
3.01
2.10
8.33
3.07
10
8
6
4
2
0
PPM

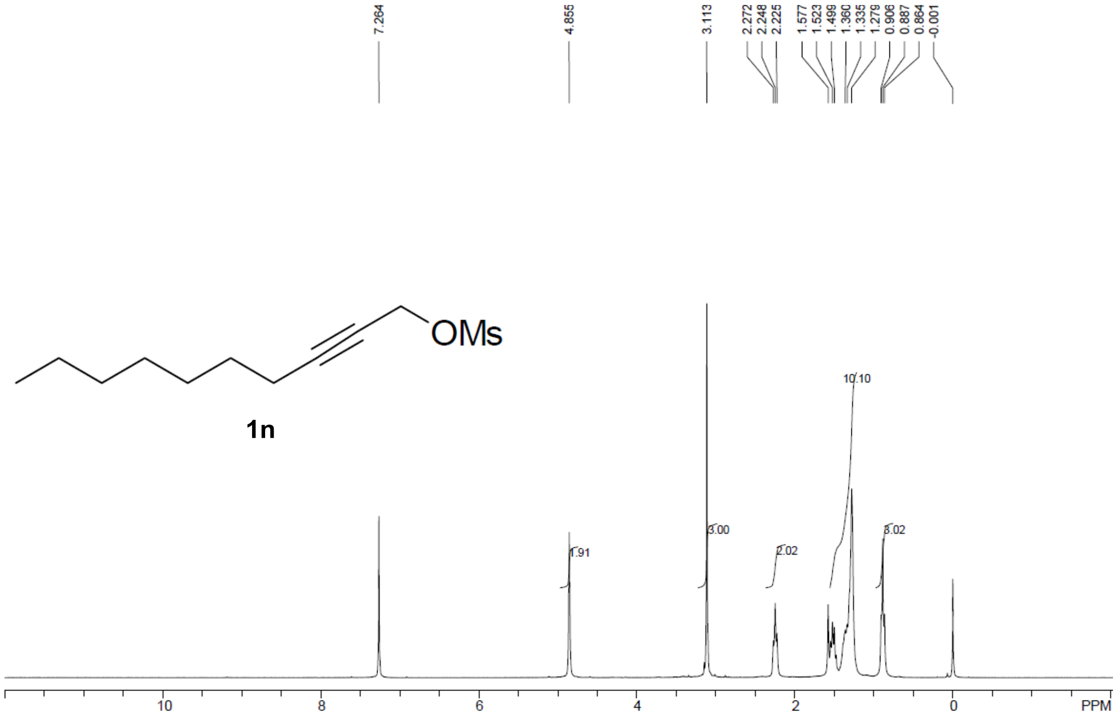
7.264
4.855
3.113
2.272
2.248
2.225
1.577
1.523
1.499
1.360
1.335
1.279
0.906
0.887
0.864
-0.001
OMs
1n
1.91
3.00
2.02
10.10
3.02
10
8
6
4
2
0
PPM

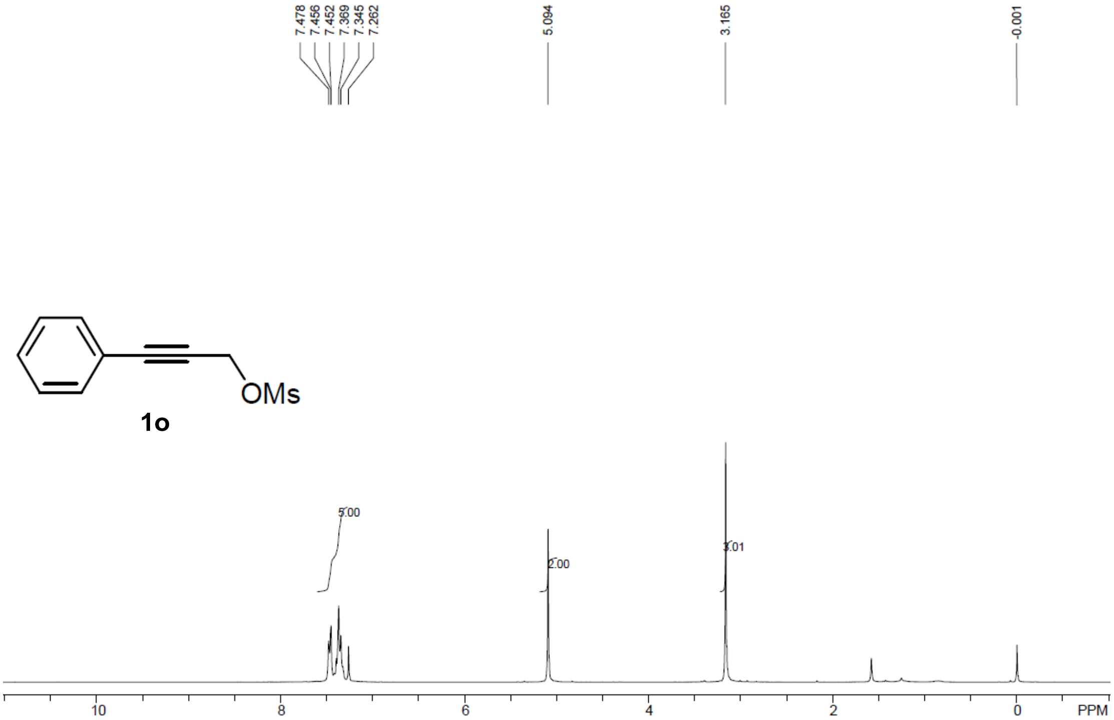
7.478
7.456
7.452
7.369
7.345
7.262
5.094
3.165
-0.001
5.00
2.00
3.01
10
8
6
4
2
0
PPM
OMs

1o

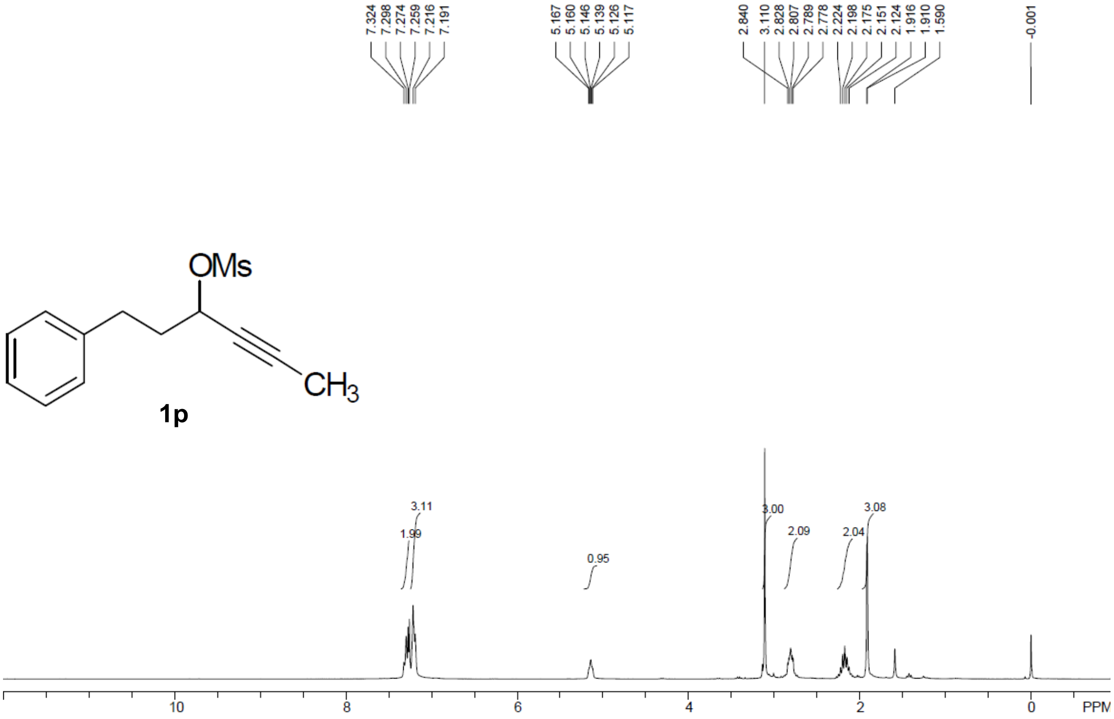
7.324
7.298
7.274
7.259
7.216
7.191
5.167
5.160
5.146
5.139
5.126
5.117
2.840
3.110
2.828
2.807
2.789
2.778
2.224
2.198
2.175
2.151
2.124
1.916
1.910
1.590
-0.001
3.11
1.99
0.95
3.00
2.09
2.04
3.08
10
8
6
4
2
0
PPM
OMs
CH3

1p

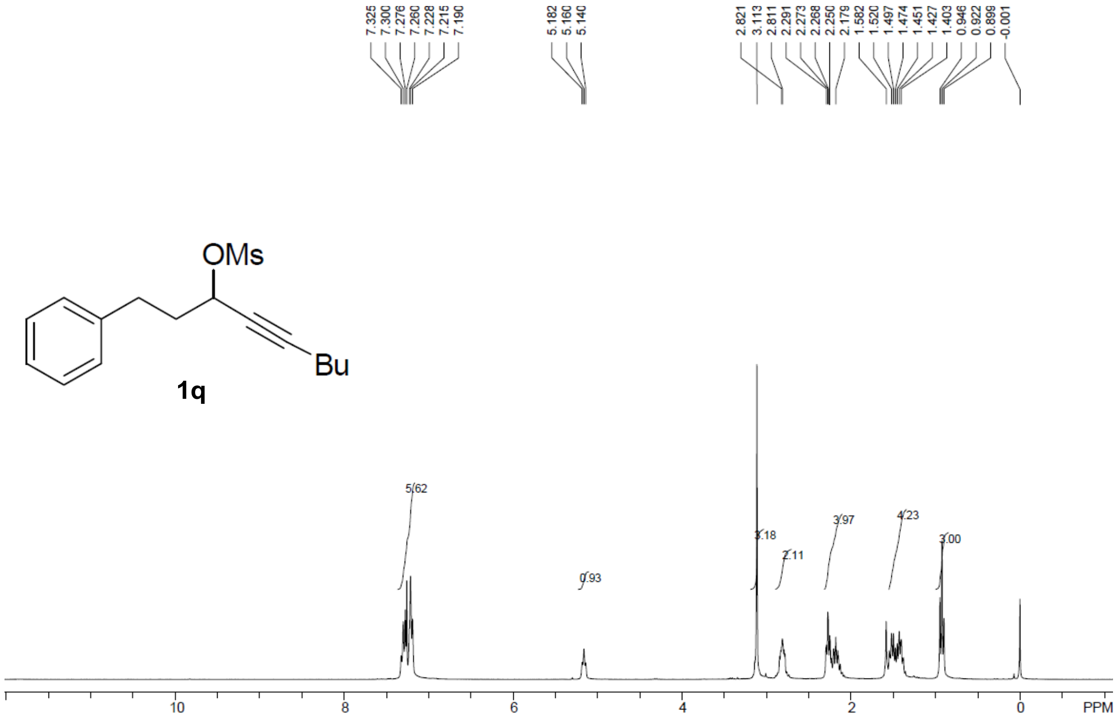
7.325
7.300
7.276
7.260
7.228
7.215
7.190
5.182
5.160
5.140
2.821
3.113
2.811
2.291
2.273
2.268
2.250
2.179
1.582
1.520
1.497
1.474
1.451
1.427
1.403
0.946
0.922
0.899
-0.001
OMs
Bu
5.62
0.93
3.18
2.11
3.97
4.23
3.00
10
8
6
4
2
0
PPM

1q

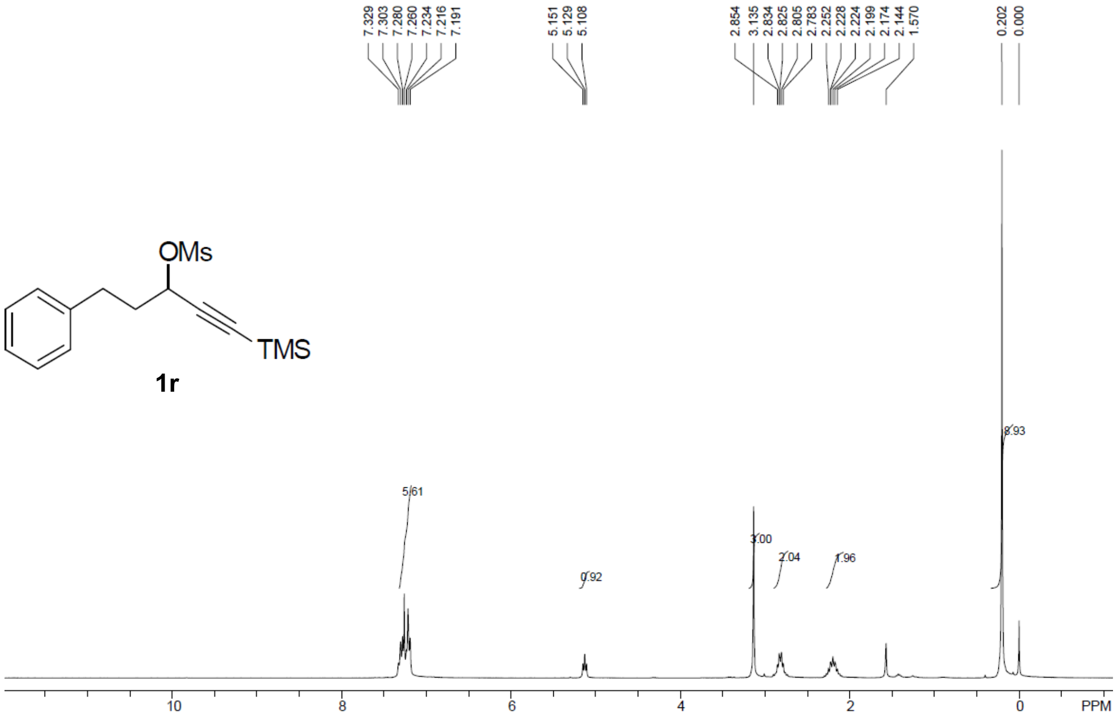
7.329
7.303
7.280
7.260
7.234
7.216
7.191
5.151
5.129
5.108
2.854
3.135
2.834
2.825
2.805
2.783
2.252
2.228
2.224
2.199
2.174
2.144
1.570
0.202
0.000
OMs
TMS
5.61
0.92
3.00
2.04
1.96
8.93
10
8
6
4
2
0
PPM

1r

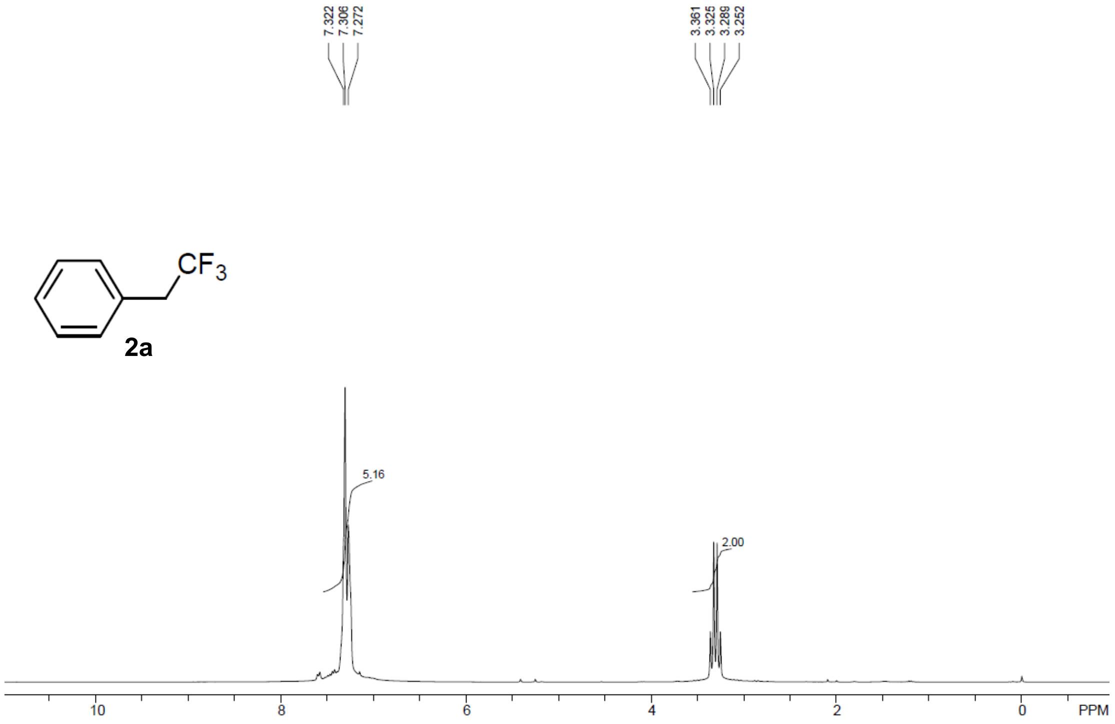
7.322
7.306
7.272
3.361
3.325
3.289
3.252
CF3
2a
5.16
2.00
10
8
6
4
2
0
PPM

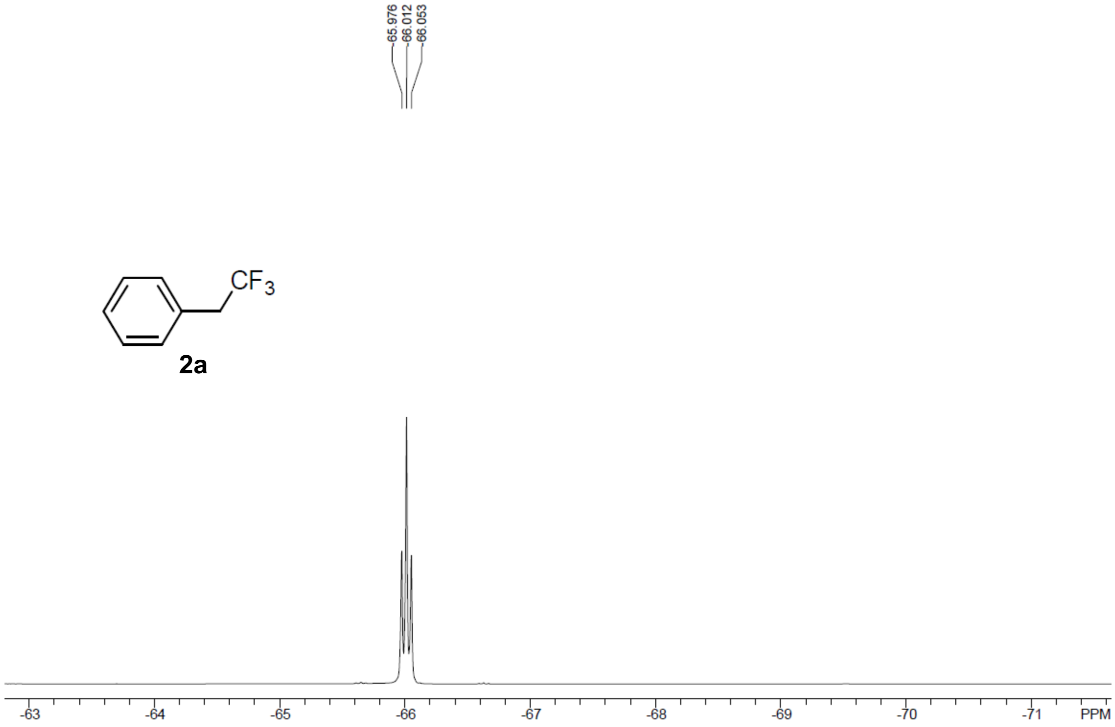
65.976
66.012
66.053
CF3
2a
-63
-64
-65
-66
-67
-68
-69
-70
-71
PPM

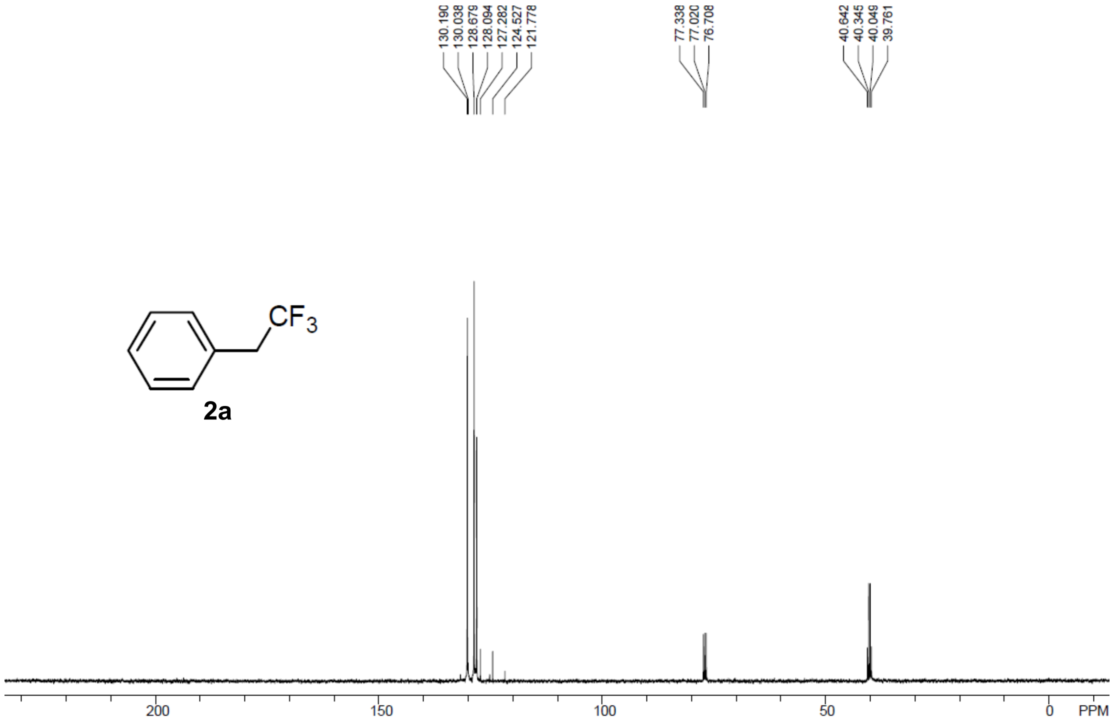

2a

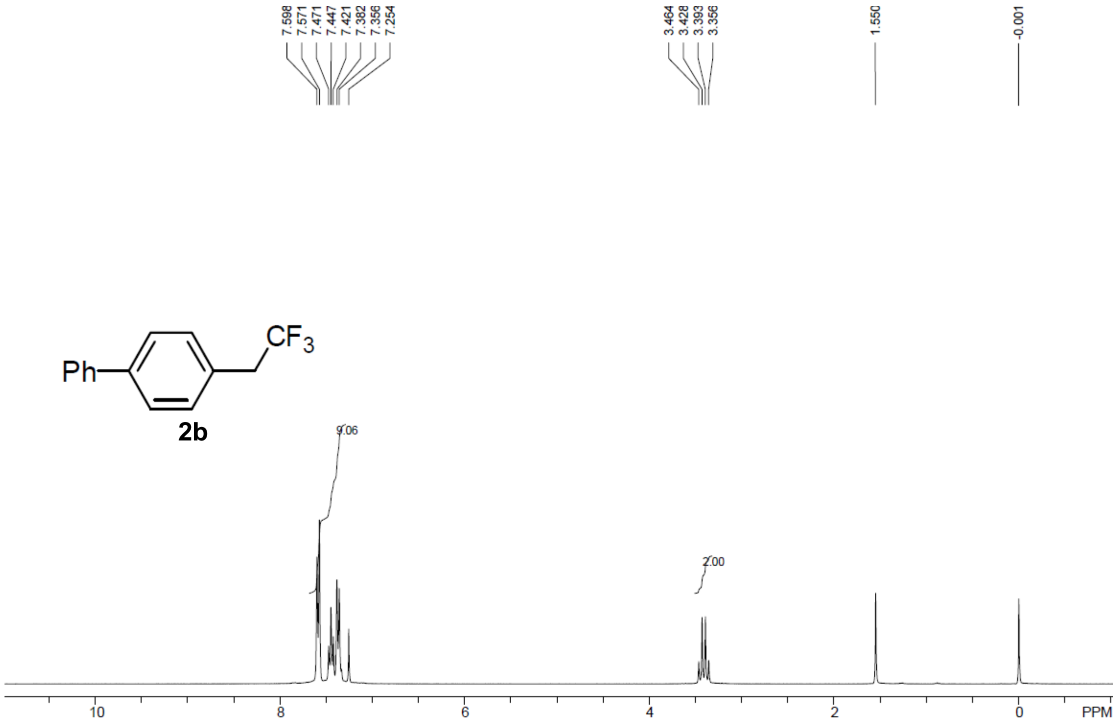

2b

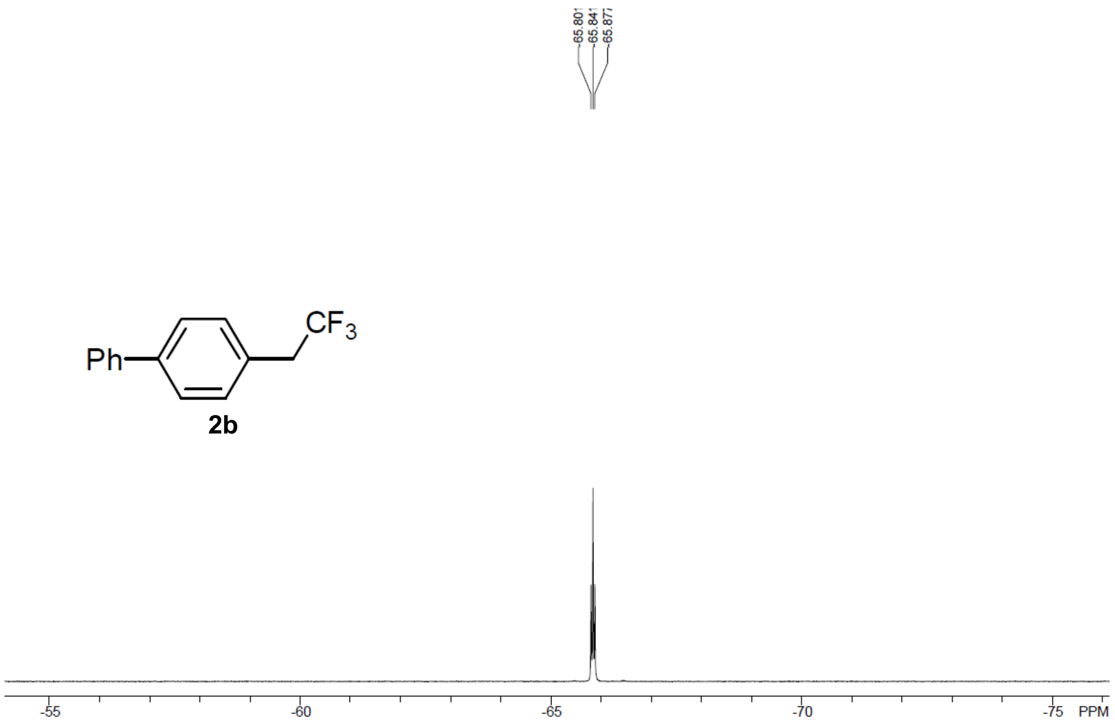

-65.801
-65.841
-65.877
Ph
CF3
2b
-55
-60
-65
-70
-75
PPM

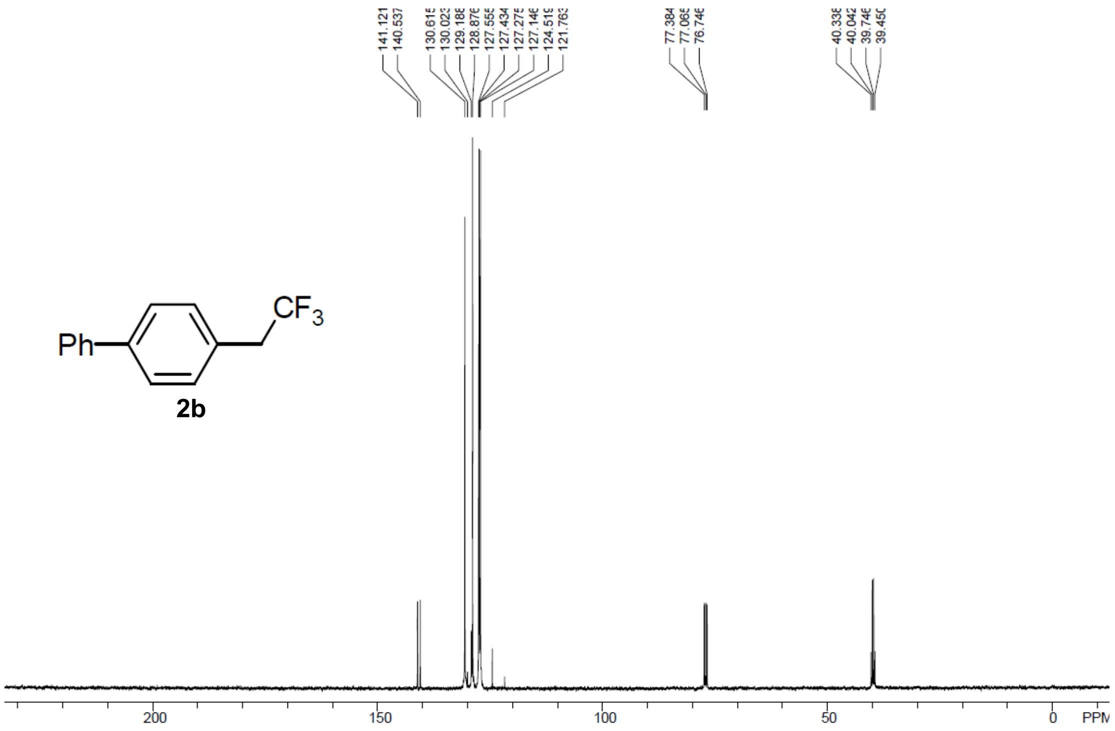

141.121
140.537
121.763
77.384
40.042
Ph
CF3
2b
200
150
100
50
0

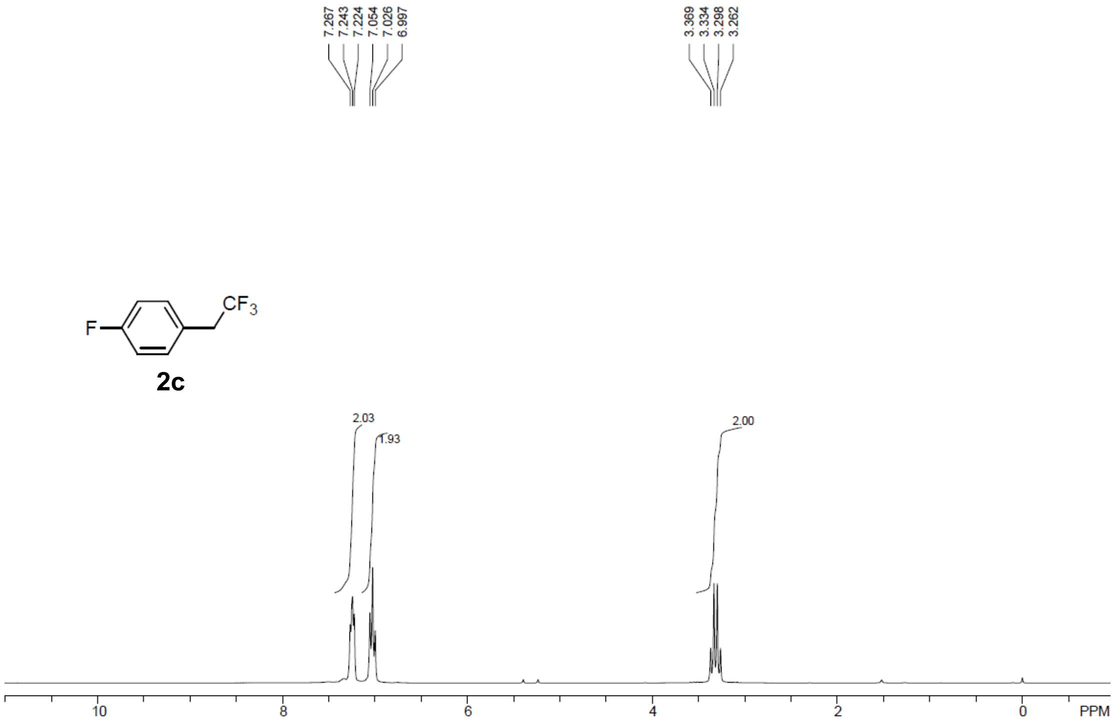
7.267
7.243
7.224
7.054
7.026
6.997
3.369
3.334
3.298
3.262
F
CF3
2c
2.03
1.93
2.00
10
8
6
4
2
0
PPM

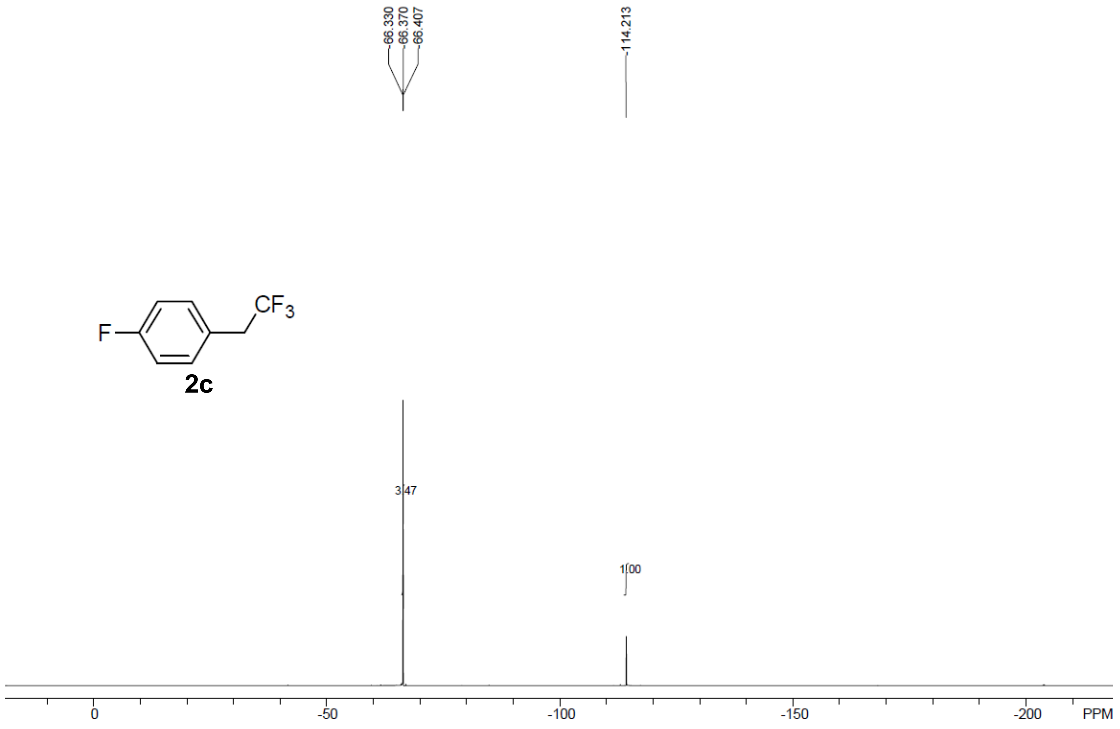
-66.330
-66.370
-66.407
-114.213
F
CF3
2c
3.47
1.00
0
-50
-100
-150
-200
PPM

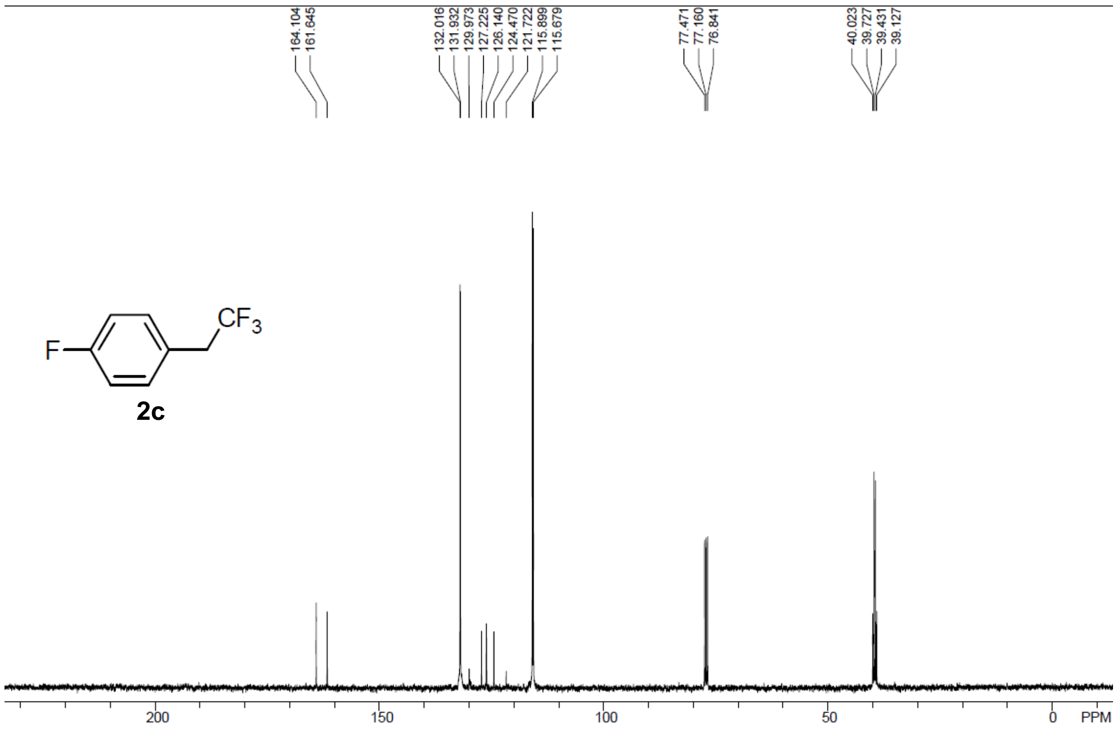

164.104
161.645
132.016
131.932
129.973
127.225
126.140
124.470
121.722
115.899
115.679
77.471
77.160
76.841
40.023
39.727
39.431
39.127
F
CF3
2c
200
150
100
50
0
PPM

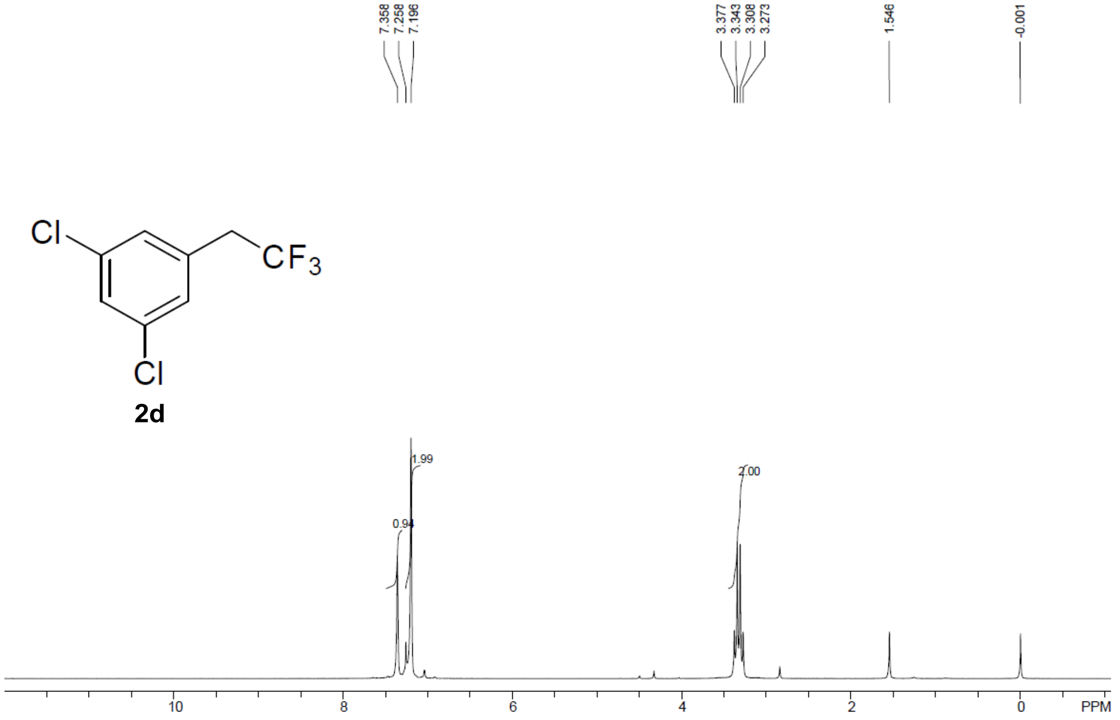

7.358
7.258
7.196
3.377
3.343
3.308
3.273
1.546
-0.001
Cl
CF3
Cl
2d
1.99
0.94
2.00
10
8
6
4
2
0
PPM

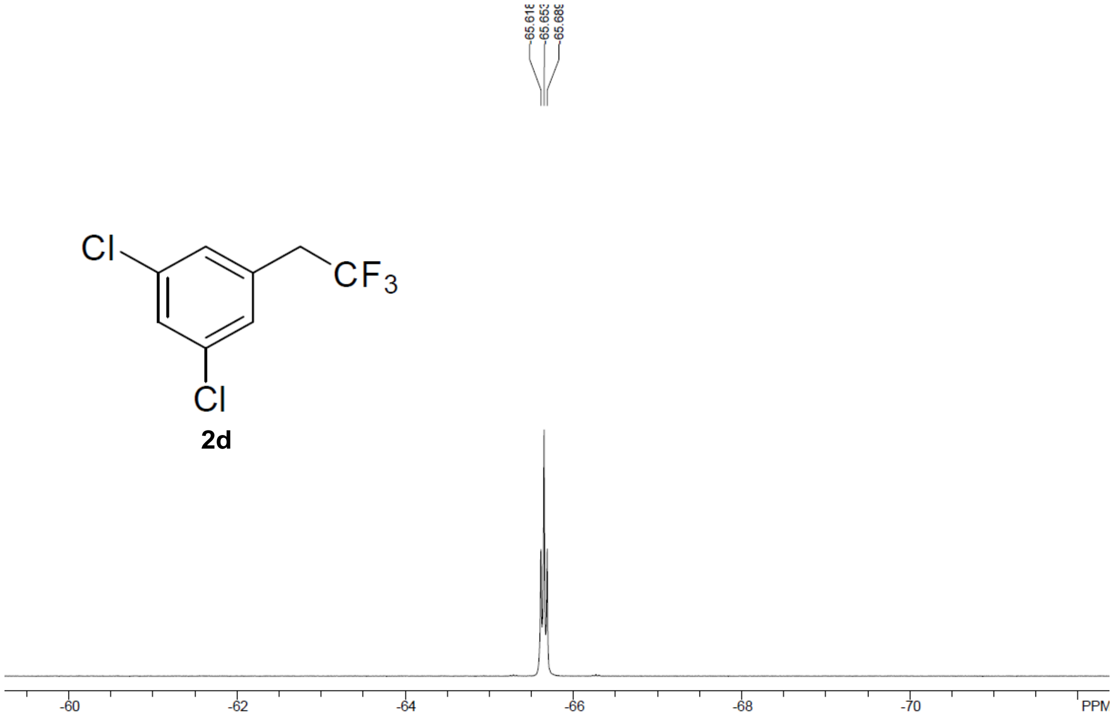
-65.618
-65.653
-65.689
Cl
CF3
Cl
2d
-60
-62
-64
-66
-68
-70
PPM

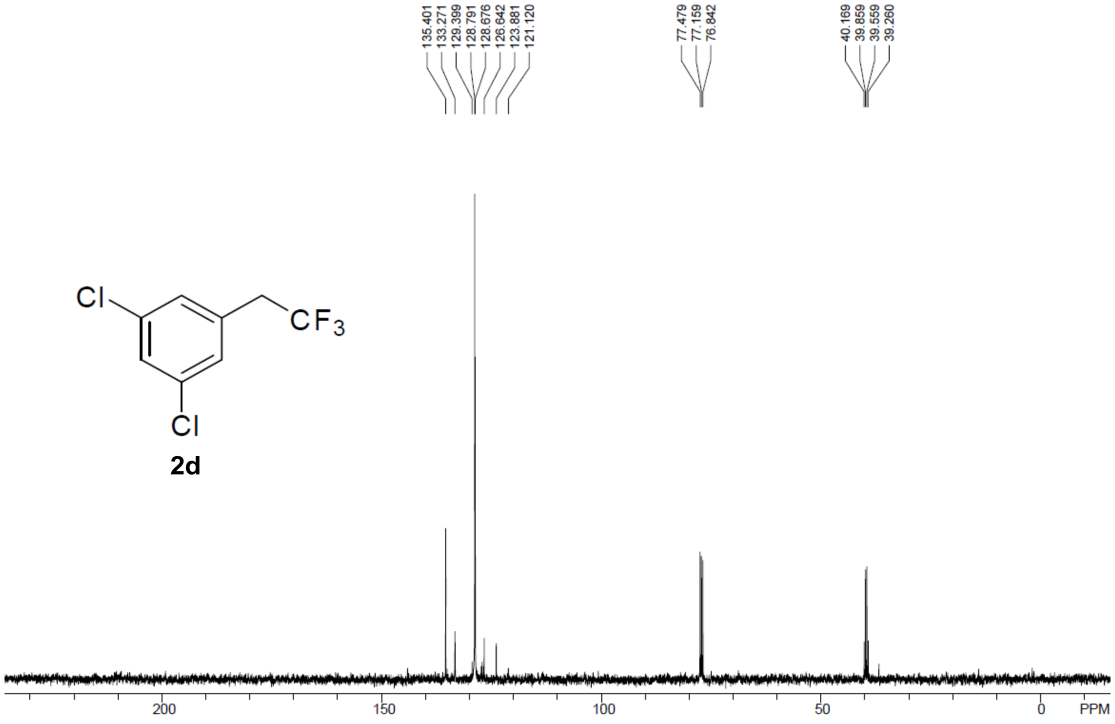
135.401
133.271
129.399
128.791
128.676
126.642
123.881
121.120
77.479
77.159
76.842
40.169
39.859
39.559
39.260
Cl
CF3
Cl
2d
200
150
100
50
0
PPM

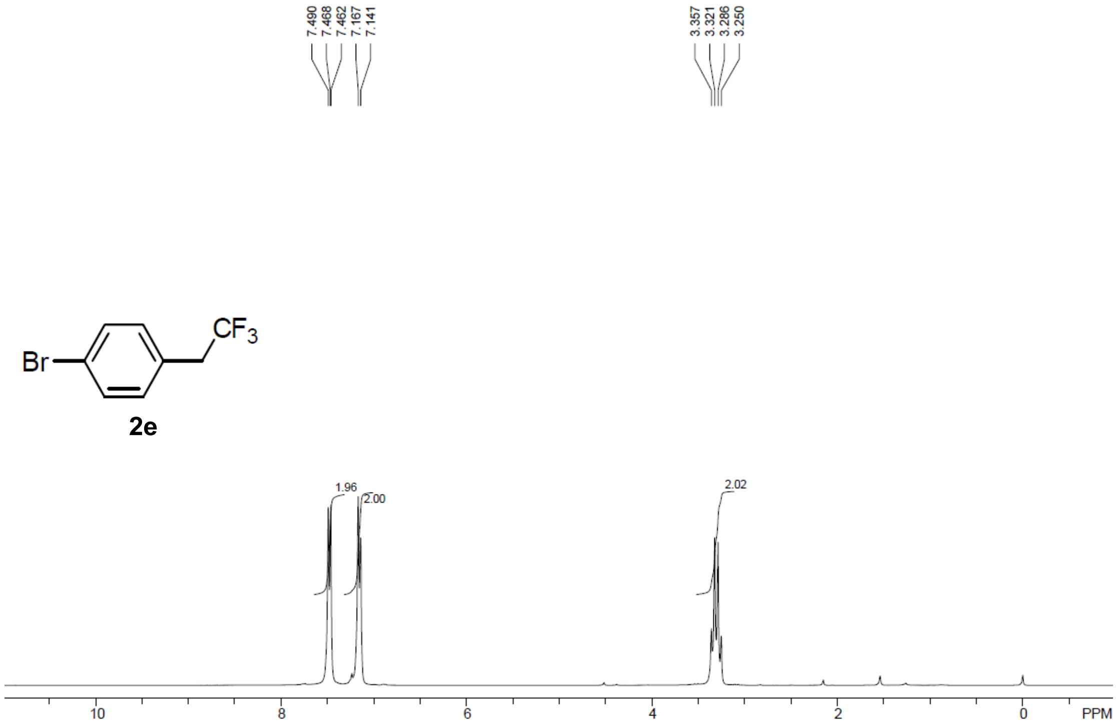
7.490
7.468
7.462
7.167
7.141
3.357
3.321
3.286
3.250
CF3
Br
2e
1.96
2.00
2.02
10
8
6
4
2
0
PPM

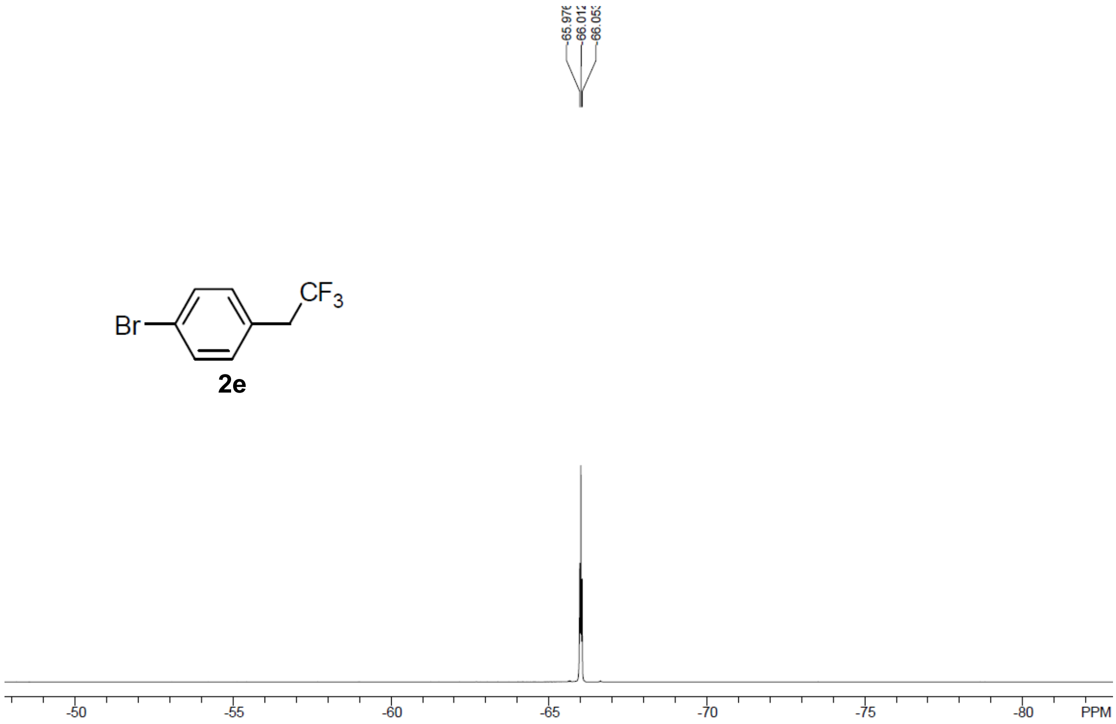
CF3
Br
2e
-50
-55
-60
-65
-70
-75
-80
PPM

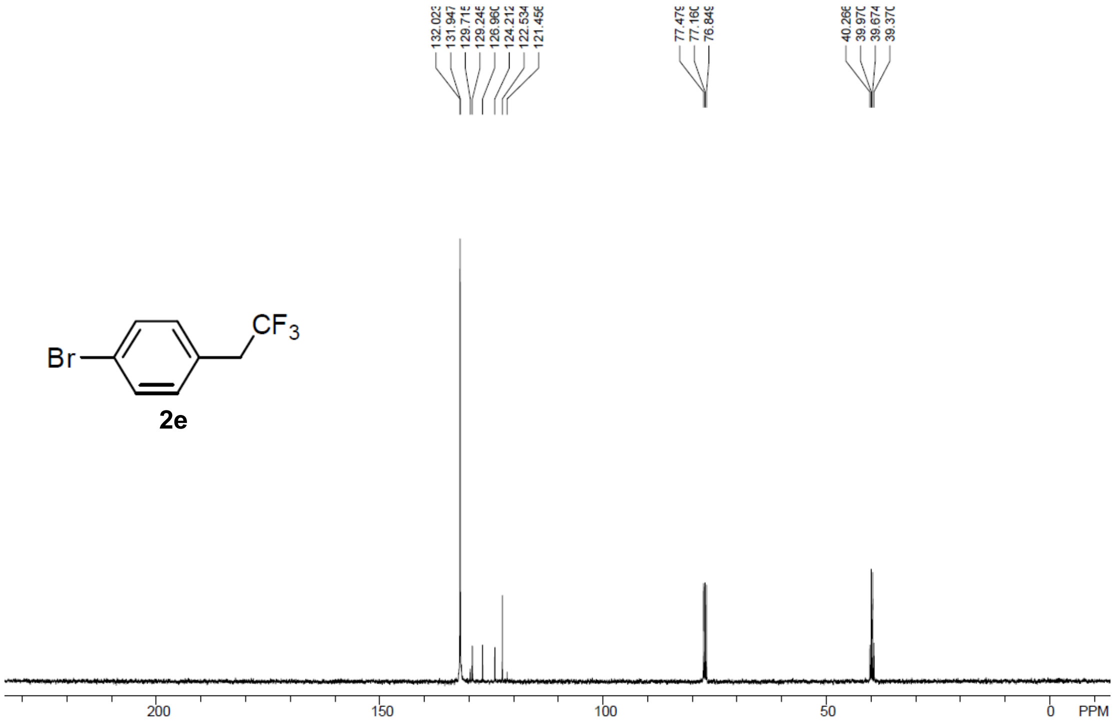

132.023
131.947
129.715
129.245
126.960
124.212
122.534
121.456
77.479
77.160
76.849
40.266
39.970
39.674
39.370
Br
CF3
200
150
100
50
0
PPM

2e

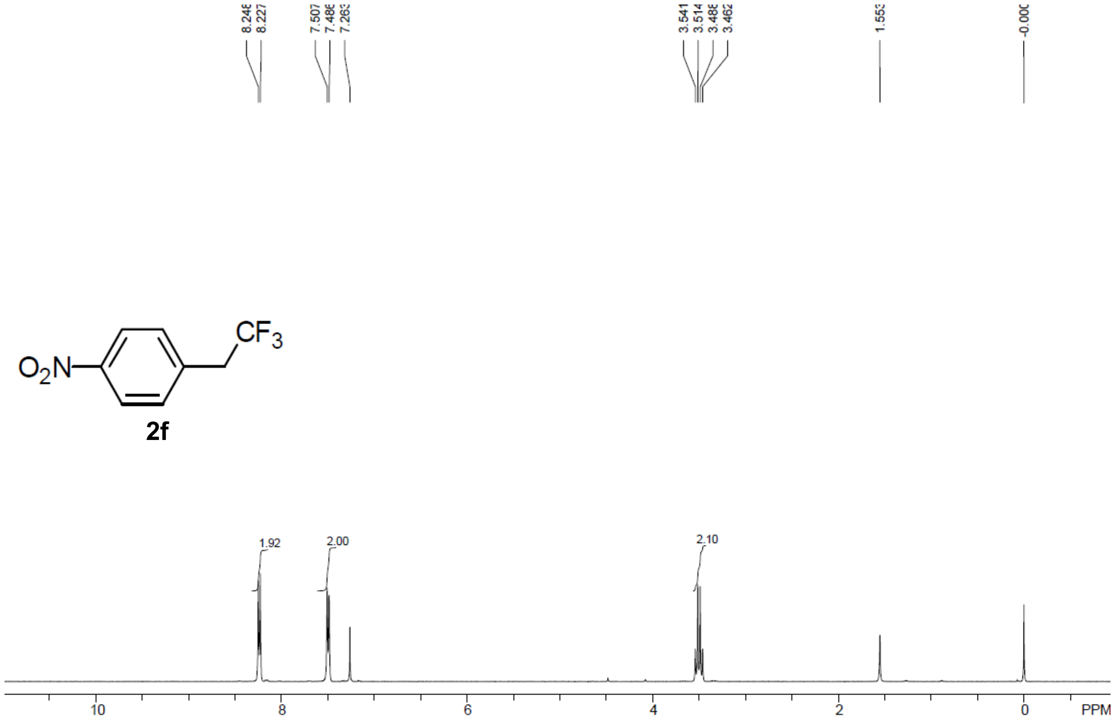

8.248
8.227
7.507
7.486
7.263
3.541
3.514
3.488
3.462
1.553
-0.000
O2N
CF3
1.92
2.00
2.10
10
8
6
4
2
0
PPM

2f

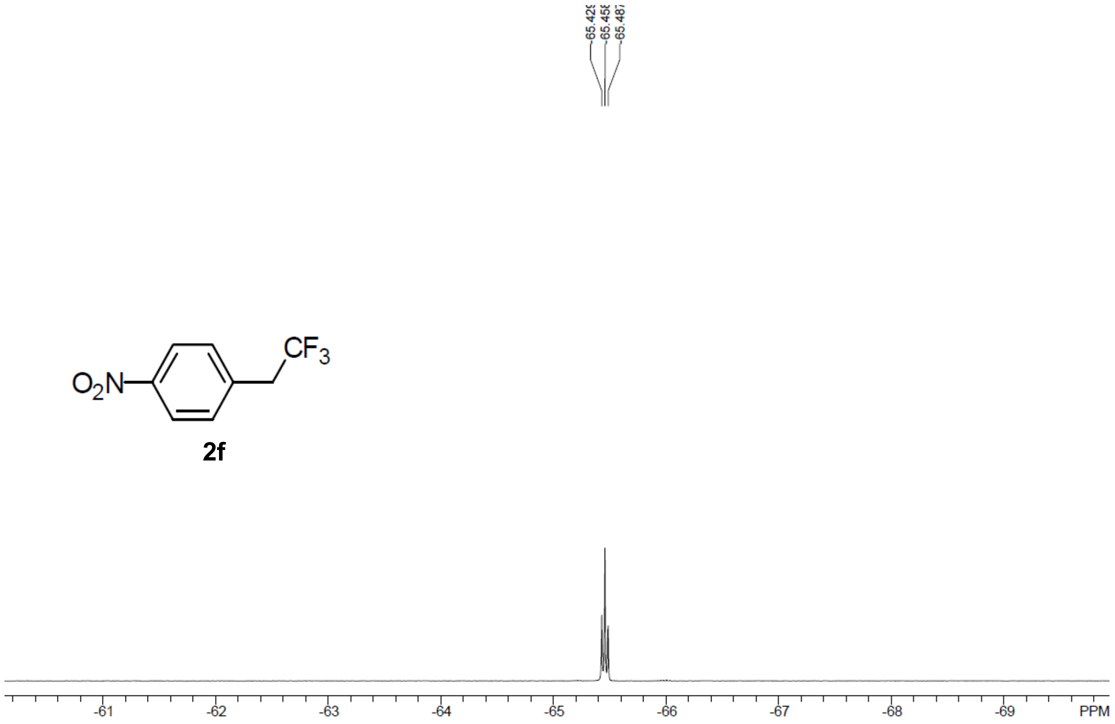
-65.429
-65.458
-65.487
O2N
CF3
2f
-61
-62
-63
-64
-65
-66
-67
-68
-69
PPM

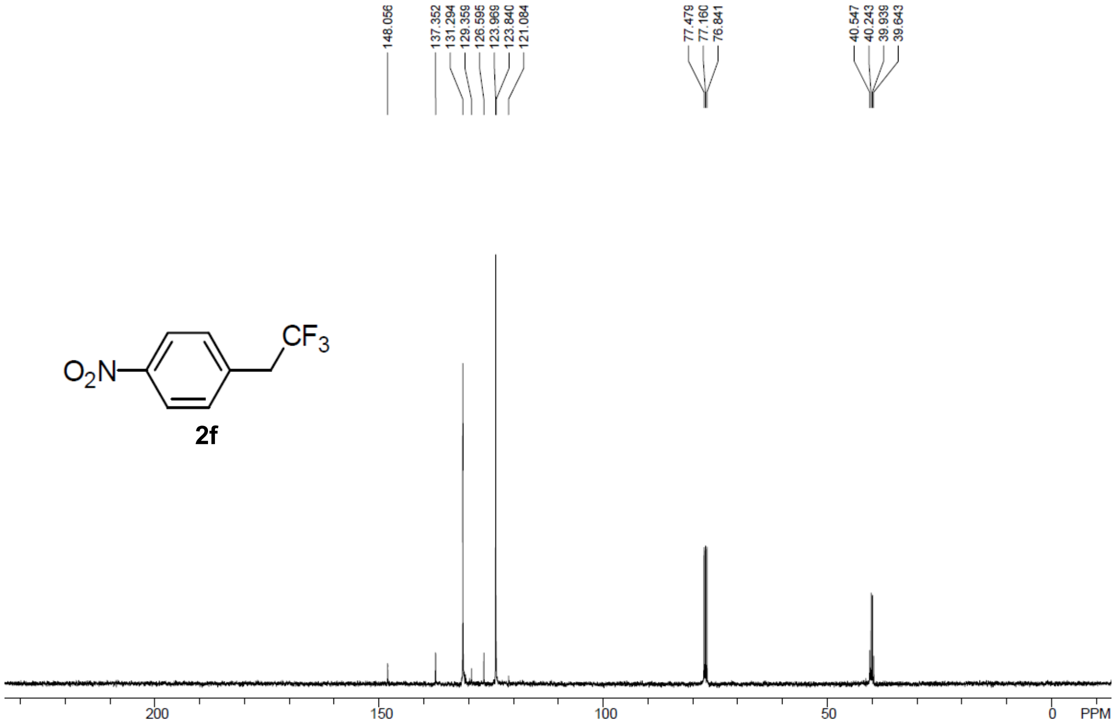
148.056
137.352
131.294
129.359
126.595
123.969
123.840
121.084
77.479
77.160
76.841
40.547
40.243
39.939
39.643
O2N
CF3
2f
200
150
100
50
0
PPM

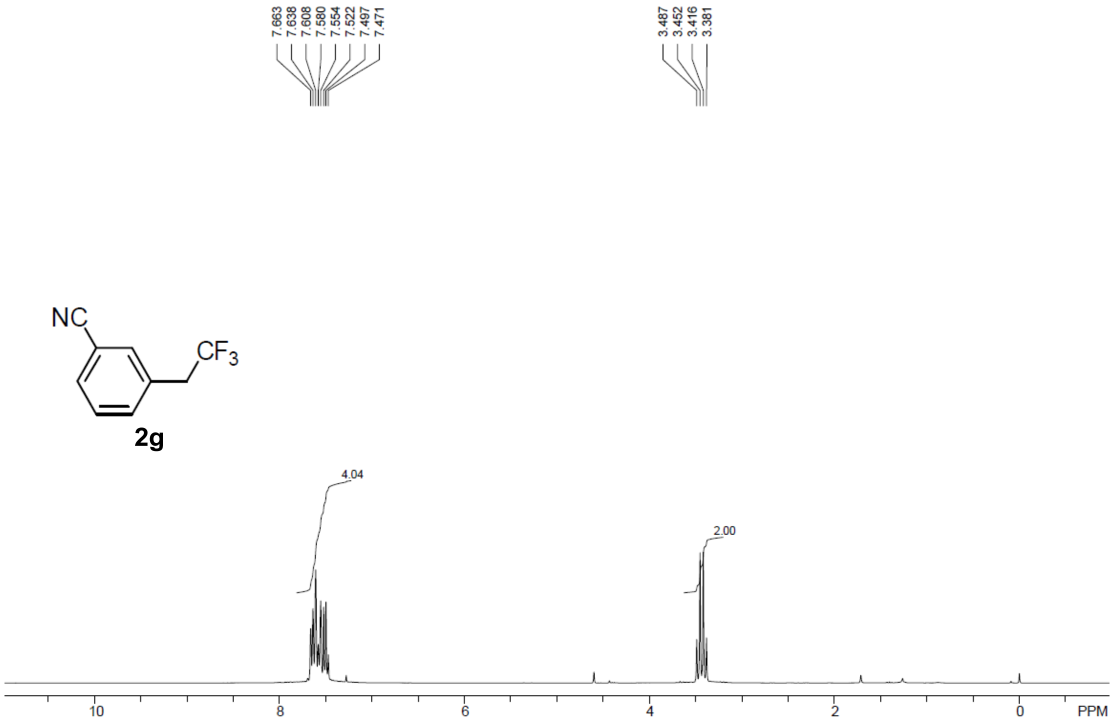
7.663
7.638
7.608
7.580
7.554
7.522
7.497
7.471
3.487
3.452
3.416
3.381
NC
CF3
2g
4.04
2.00
10
8
6
4
2
0
PPM

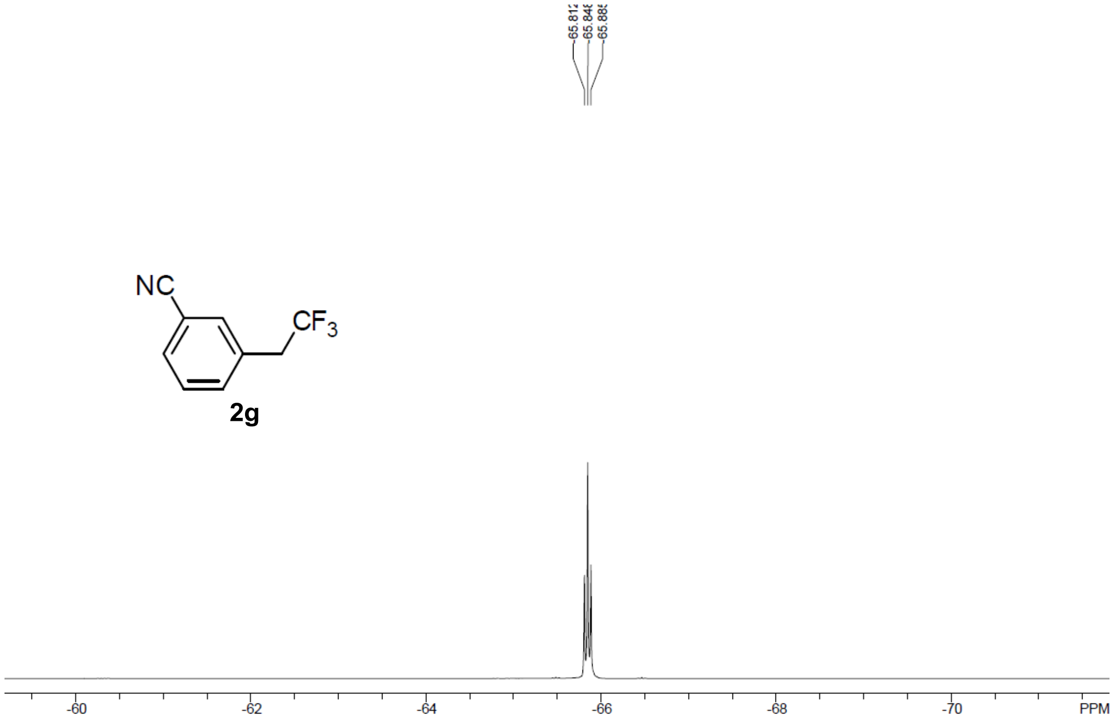
NC
CF3
2g
-60
-62
-64
-66
-68
-70
PPM

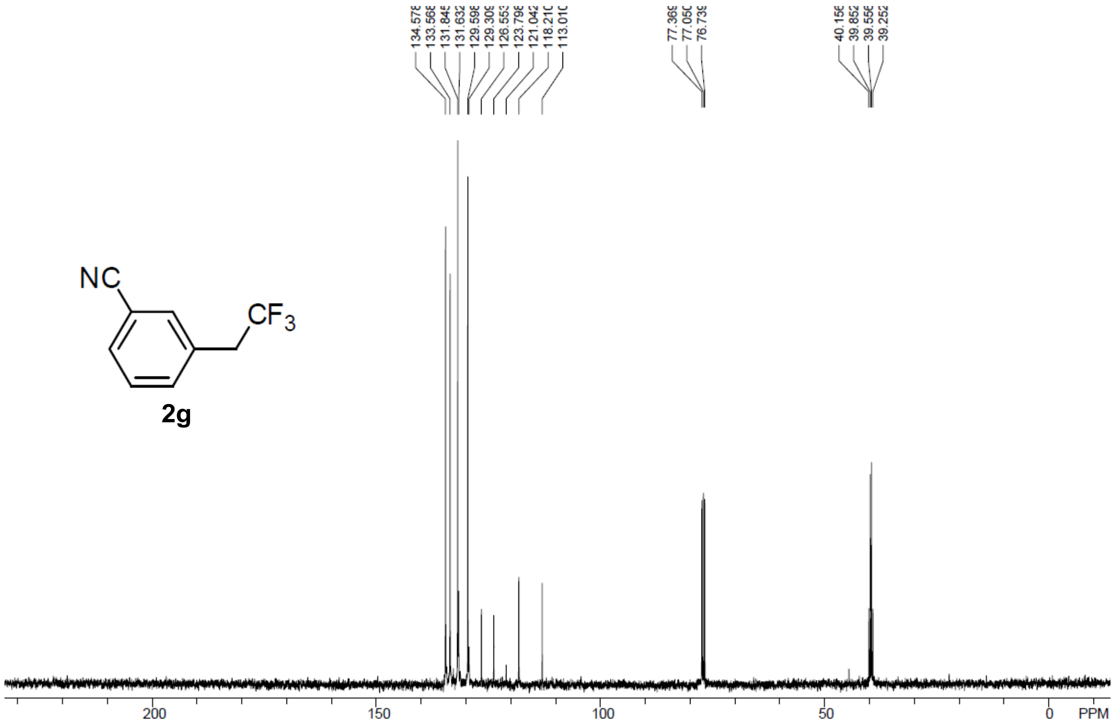
134.578
133.568
131.849
131.632
129.598
129.309
126.553
123.798
121.042
118.210
113.010
77.369
77.050
76.739
40.158
39.852
39.556
39.252
NC
CF3
200
150
100
50
0
PPM

2g

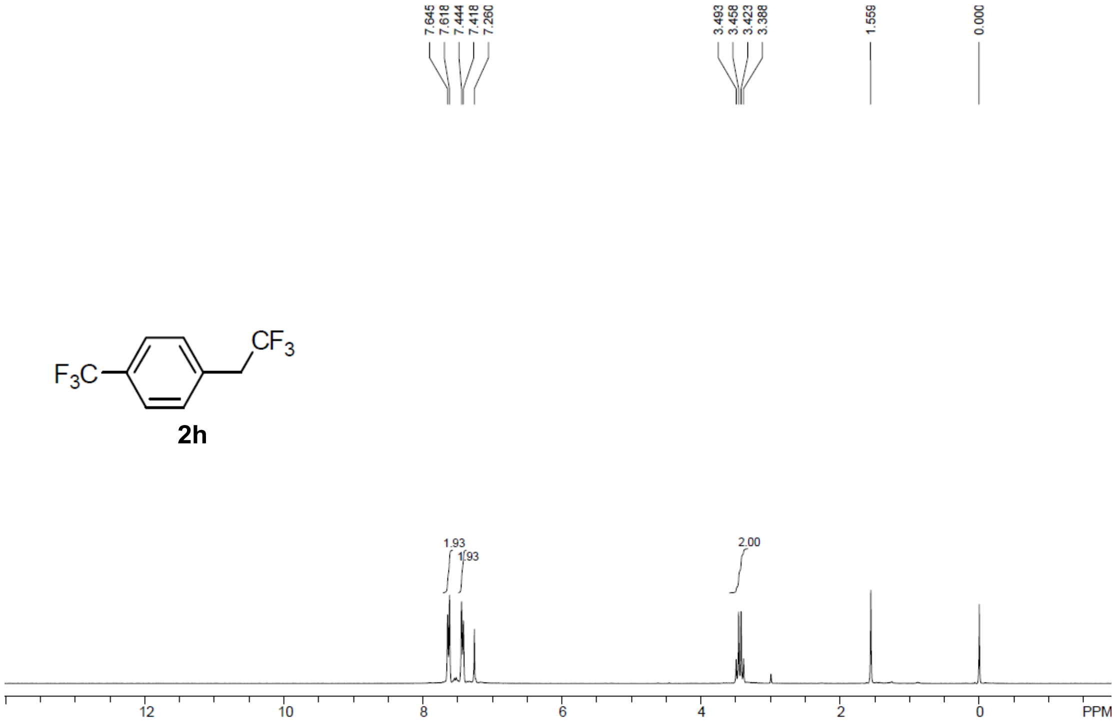
7.645
7.618
7.444
7.418
7.260
3.493
3.458
3.423
3.388
1.559
0.000
F3C
CF3
1.93
1.93
2.00
12
10
8
6
4
2
0
PPM

2h

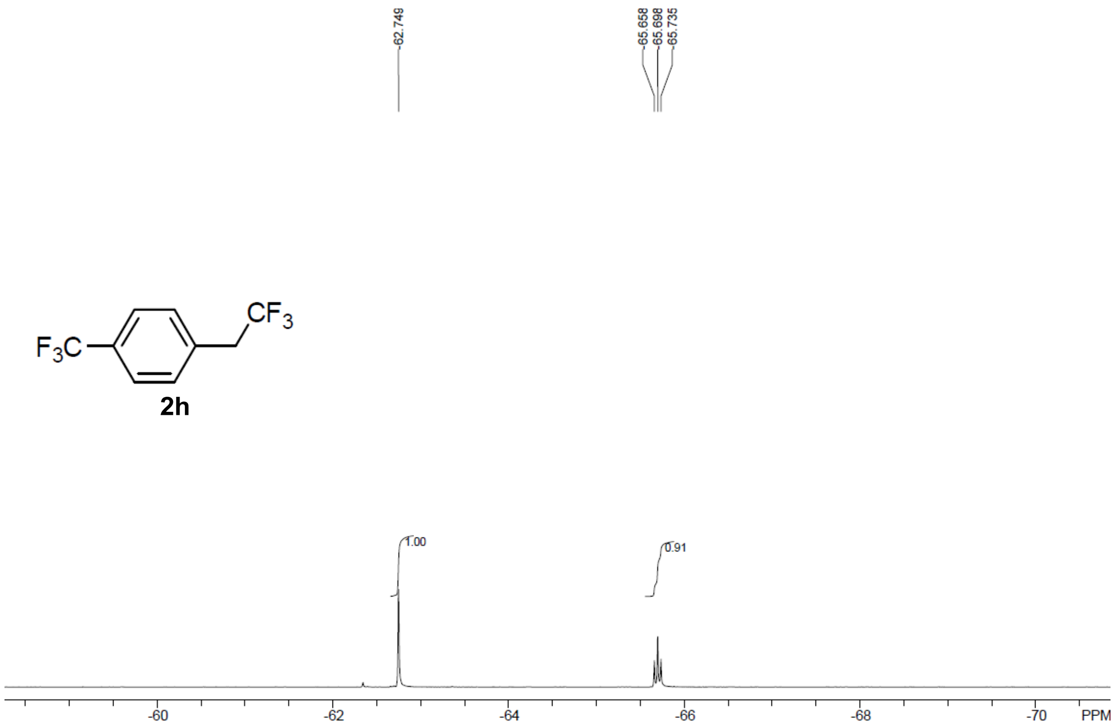

-62.749
-65.658
-65.698
-65.735
F_3C
CF_3
2h
1.00
0.91
-60
-62
-64
-66
-68
-70
PPM

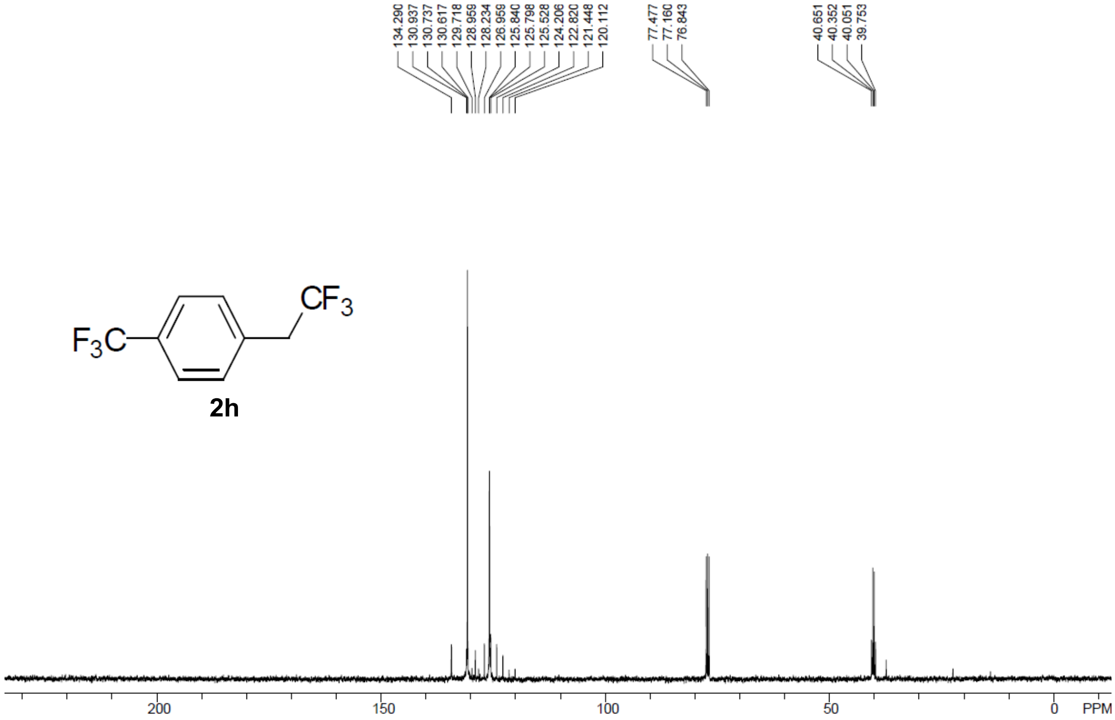

134.290
130.937
130.737
130.617
129.718
128.959
128.234
126.959
125.840
125.798
125.528
124.206
122.820
121.448
120.112
77.477
77.160
76.843
40.651
40.352
40.051
39.753
F_3C
CF_3
2h
200
150
100
50
0
PPM

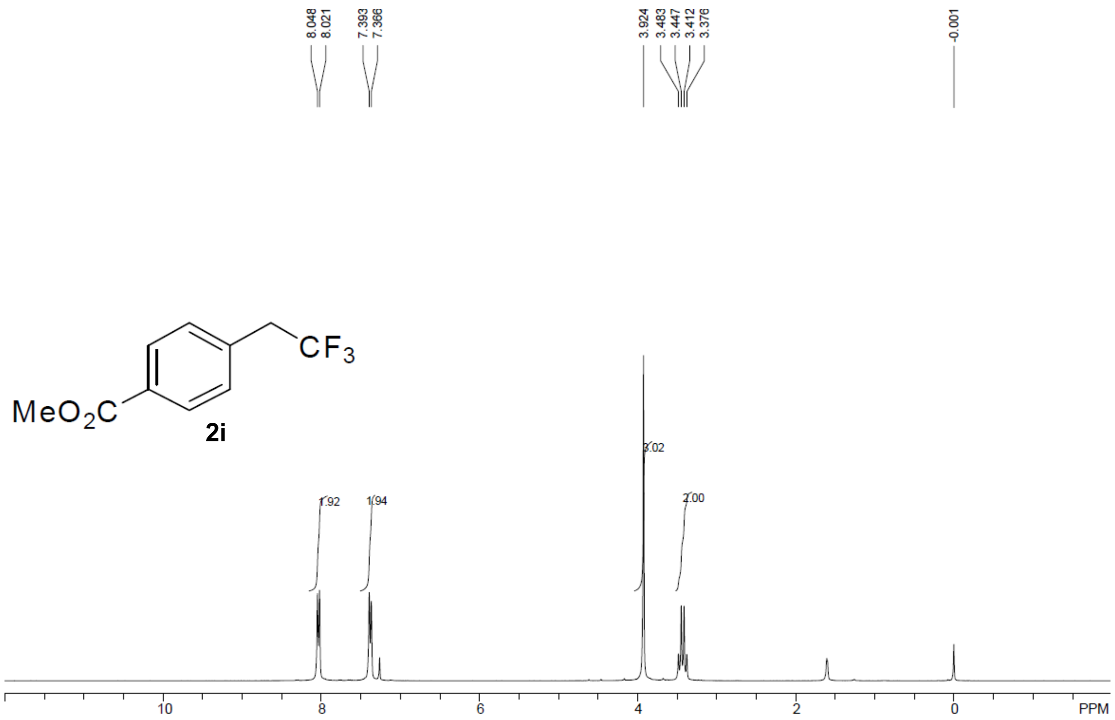
8.048
8.021
7.393
7.366
3.924
3.483
3.447
3.412
3.376
-0.001
CF3
MeO2C
2i
1.92
1.94
3.02
2.00
10
8
6
4
2
0
PPM

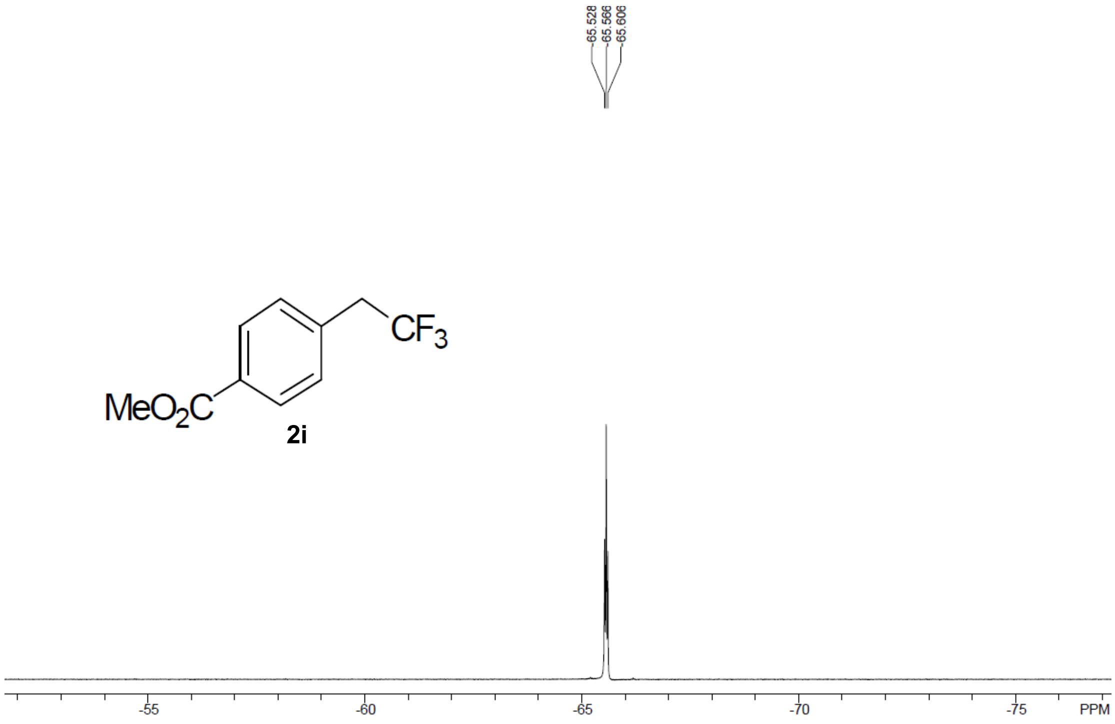
-65.528
-65.566
-65.606
CF3
MeO2C
2i
-55
-60
-65
-70
-75
PPM

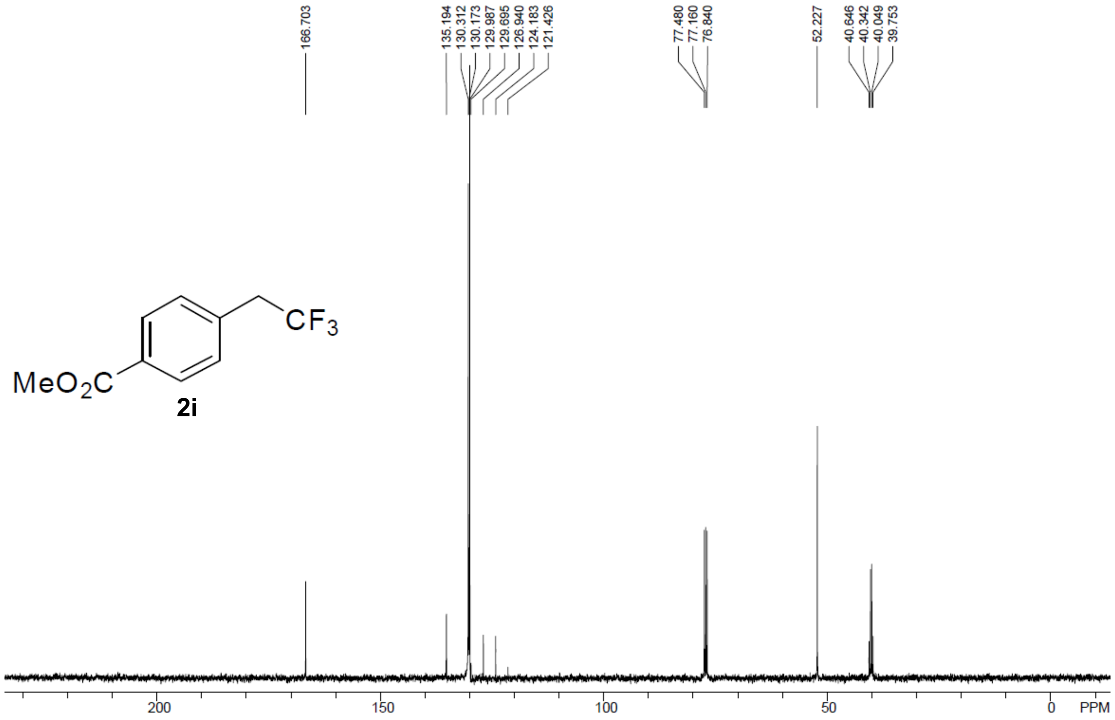
166.703
135.194
130.312
130.173
129.987
129.695
126.940
124.183
121.426
77.480
77.160
76.840
52.227
40.646
40.342
40.049
39.753
MeO2C
CF3
2i
200
150
100
50
0
PPM

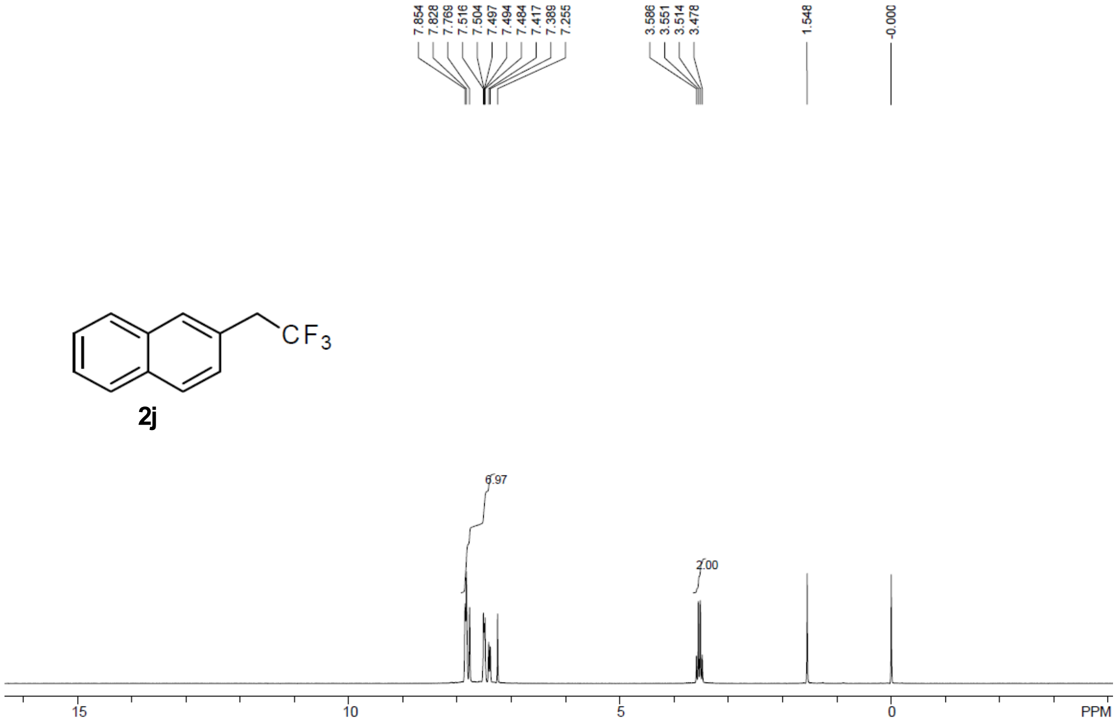
7.854
7.828
7.769
7.516
7.504
7.497
7.494
7.484
7.417
7.389
7.255
3.586
3.551
3.514
3.478
1.548
-0.000
CF3
2j
6.97
2.00
15
10
5
0
PPM

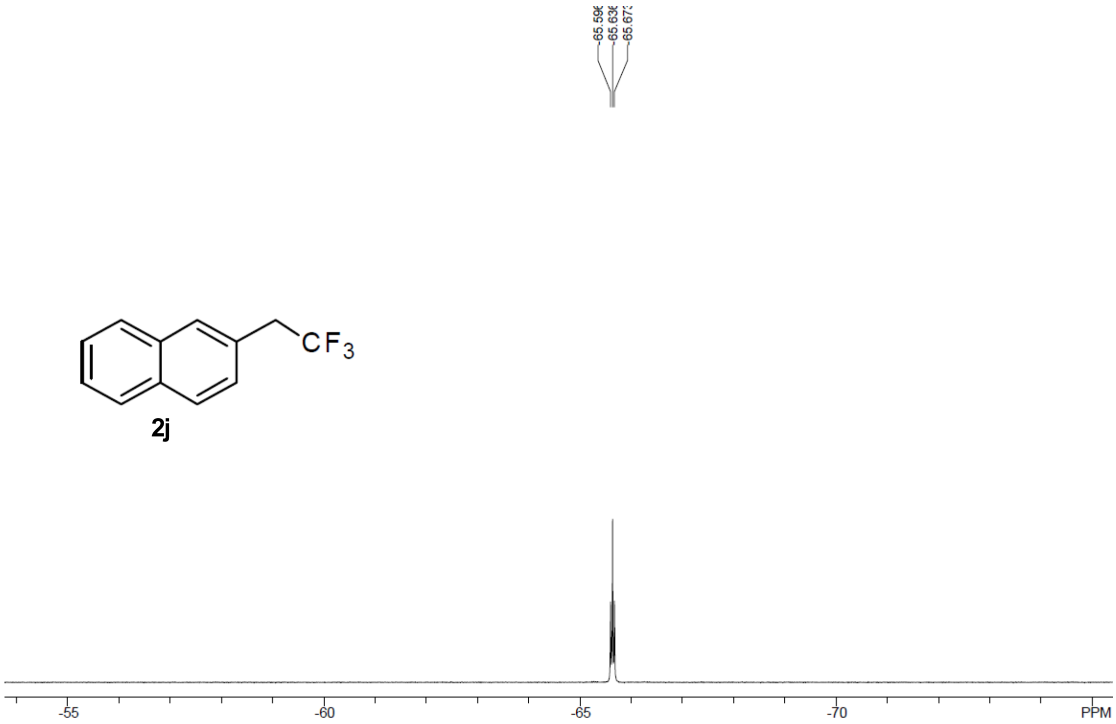
CF3
2j
-55
-60
-65
-70
PPM

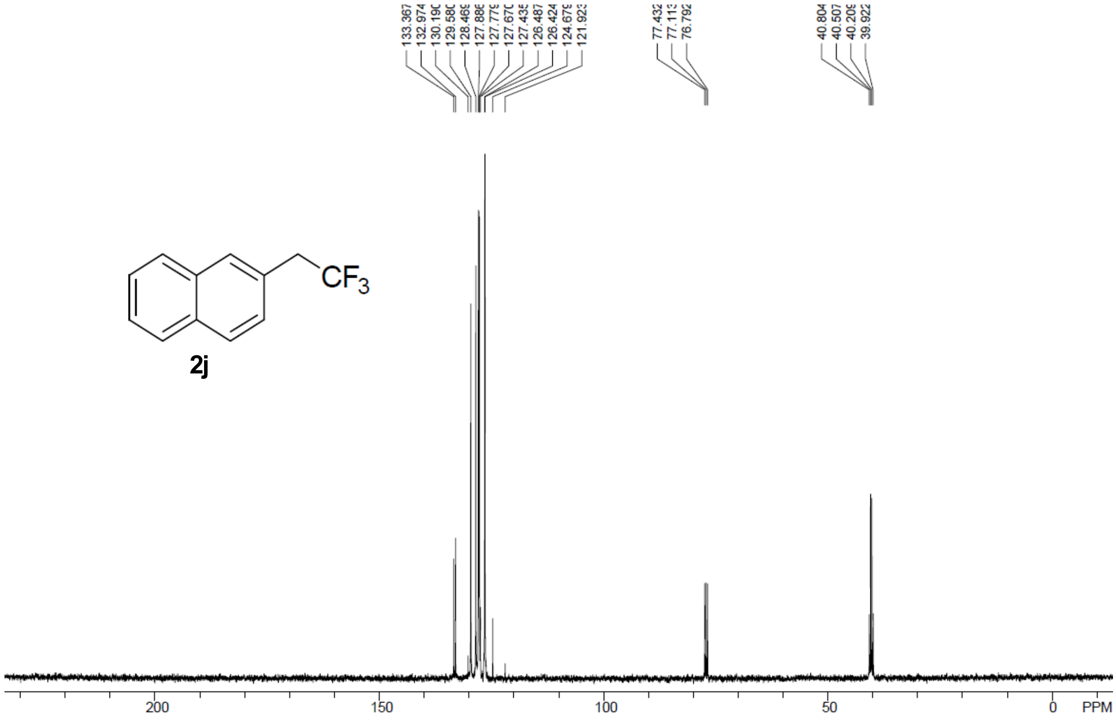
133.367
132.974
127.670
126.487
77.432
76.792
40.804
40.507
39.922
CF3
2j
200
150
100
50
0
PPM

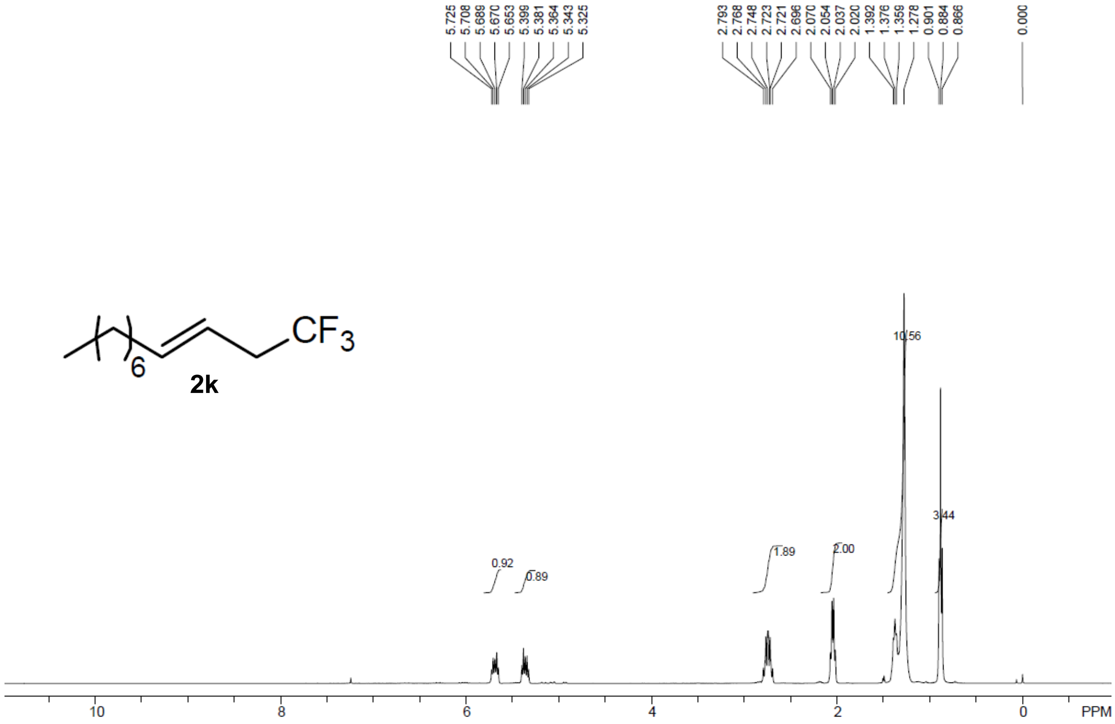
5.725
5.708
5.689
5.670
5.653
5.399
5.381
5.364
5.343
5.325
2.793
2.768
2.748
2.723
2.721
2.696
2.070
2.054
2.037
2.020
1.392
1.376
1.359
1.278
0.901
0.884
0.866
0.000
CF3
6
2k
0.92
0.89
1.89
2.00
10.56
3.44
10
8
6
4
2
0
PPM

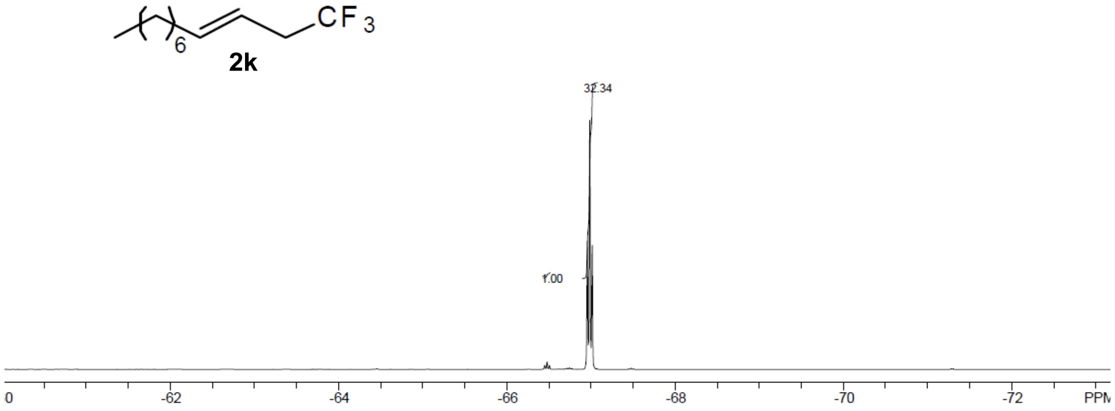
CF 3
6
2k
32.34
1.00
0
-62
-64
-66
-68
-70
-72
PPM

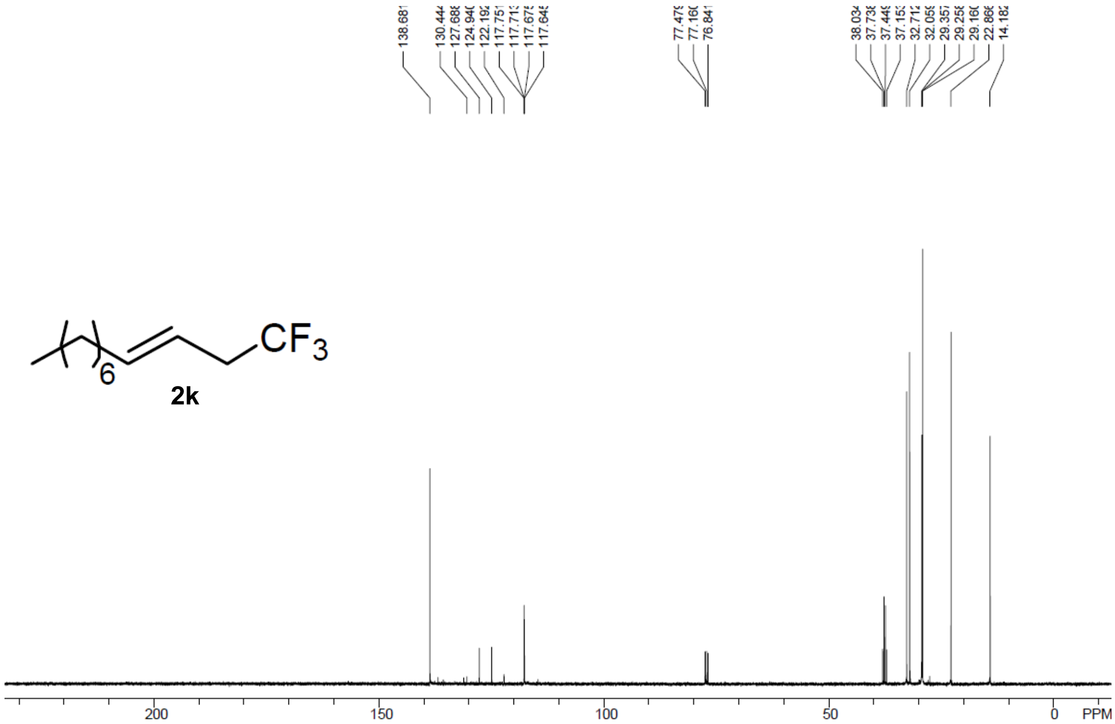
138.681
130.444
127.688
124.940
122.192
117.751
117.713
117.673
117.649
77.479
77.160
76.841
38.034
37.738
37.449
37.153
32.712
32.059
29.357
29.258
29.160
22.866
14.182
CF3
6
2k
200
150
100
50
0
PPM

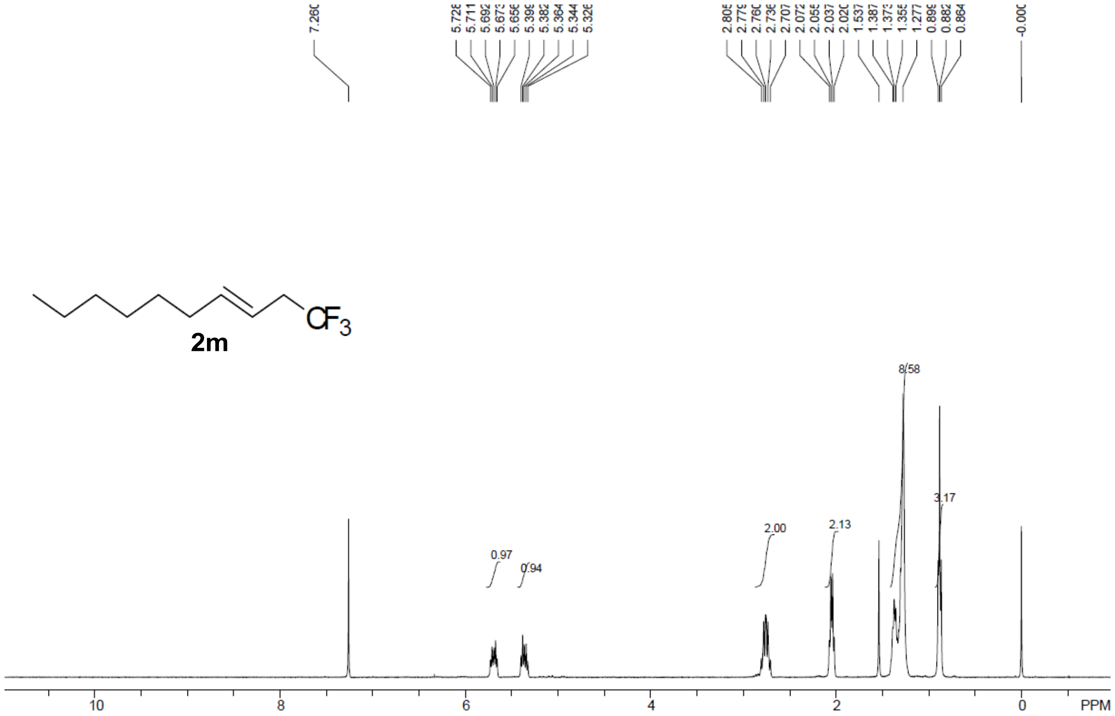
7.260
5.728
5.711
5.692
5.673
5.656
5.399
5.382
5.364
5.344
5.326
2.805
2.779
2.760
2.736
2.707
2.072
2.055
2.037
2.020
1.537
1.387
1.373
1.355
1.277
0.899
0.882
0.864
-0.000
CF3
2m
0.97
0.94
2.00
2.13
8.58
3.17
10
8
6
4
2
0
PPM

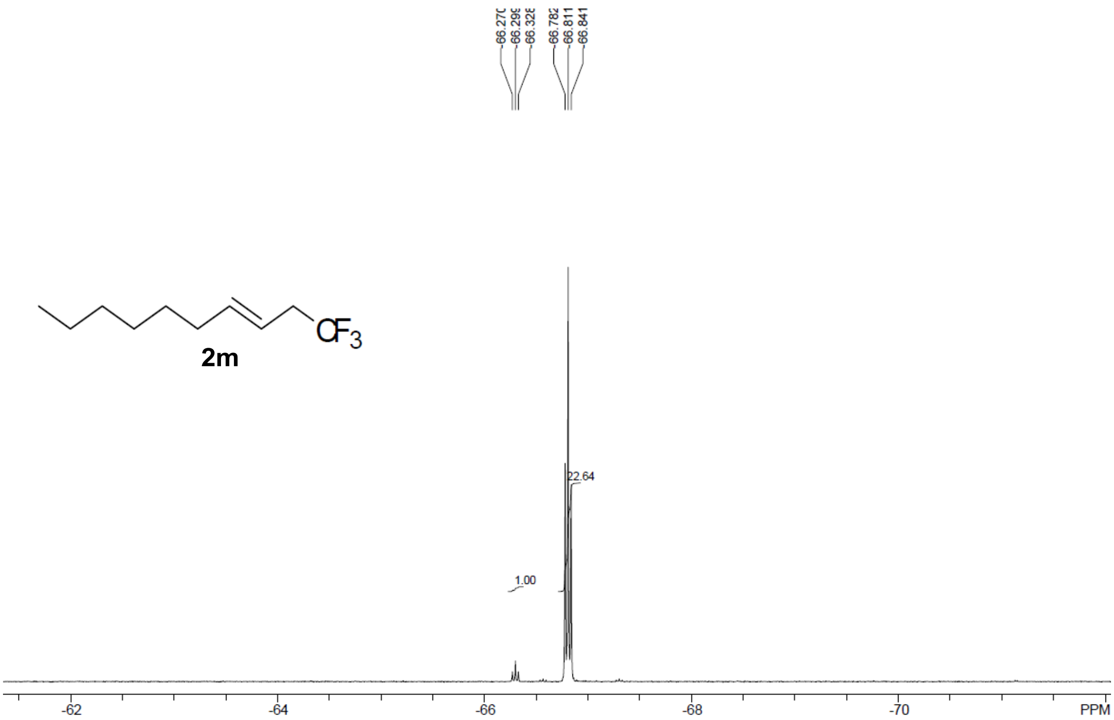
CF3
2m
22.64
1.00
-62
-64
-66
-68
-70
PPM

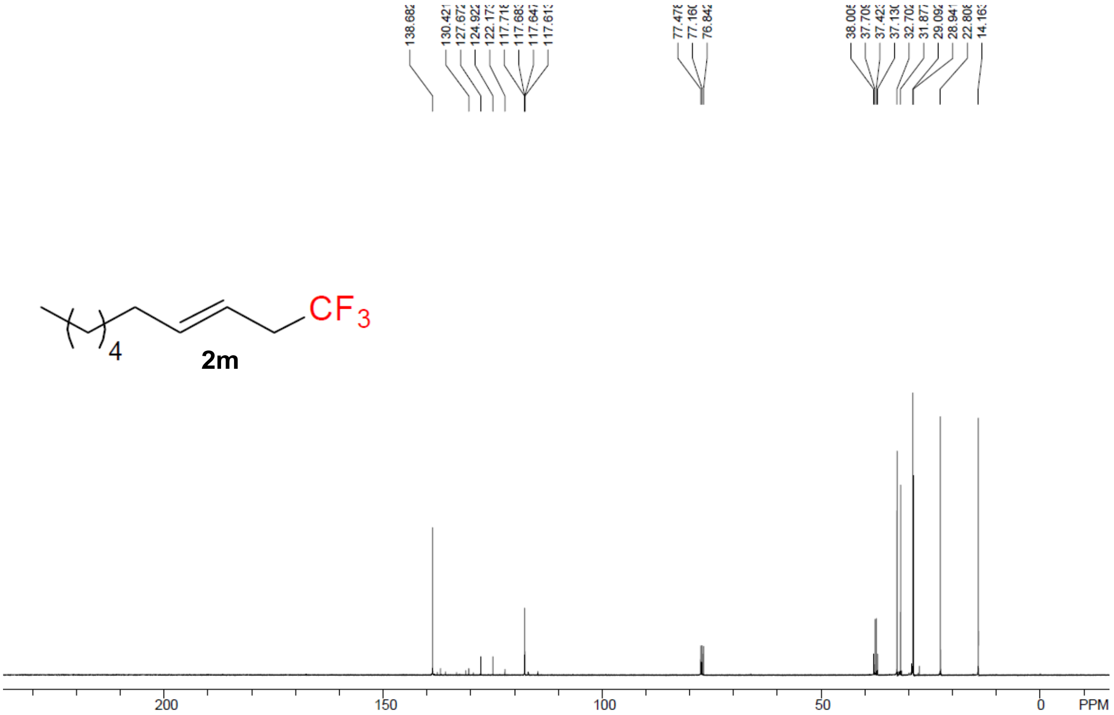
CF3
4
2m
200
150
100
50
0
PPM

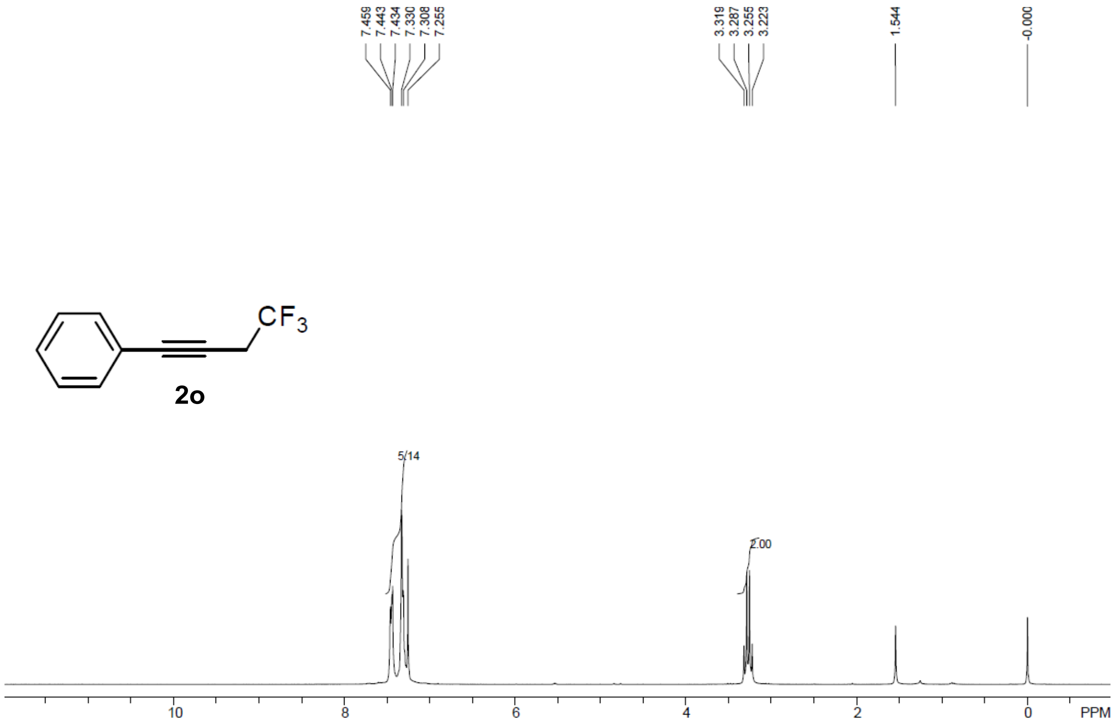
7.459
7.443
7.434
7.330
7.308
7.255
3.319
3.287
3.255
3.223
1.544
-0.000
CF3
2o
5.14
2.00
10
8
6
4
2
0
PPM

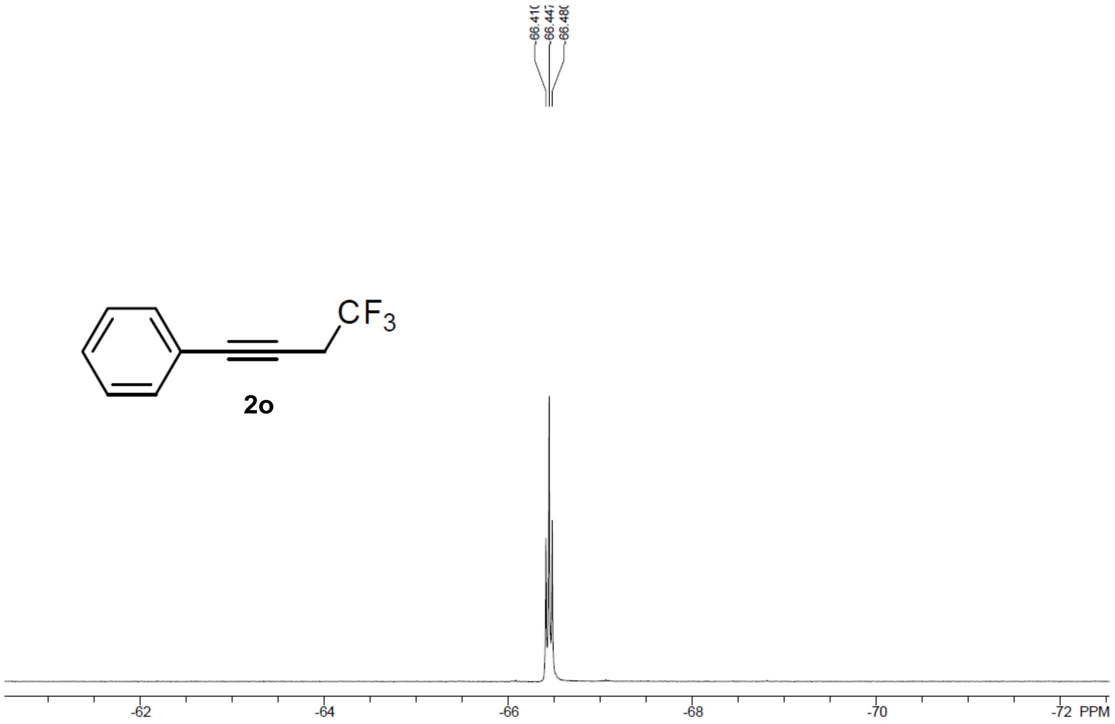
CF3
2o
-62
-64
-66
-68
-70
-72
PPM

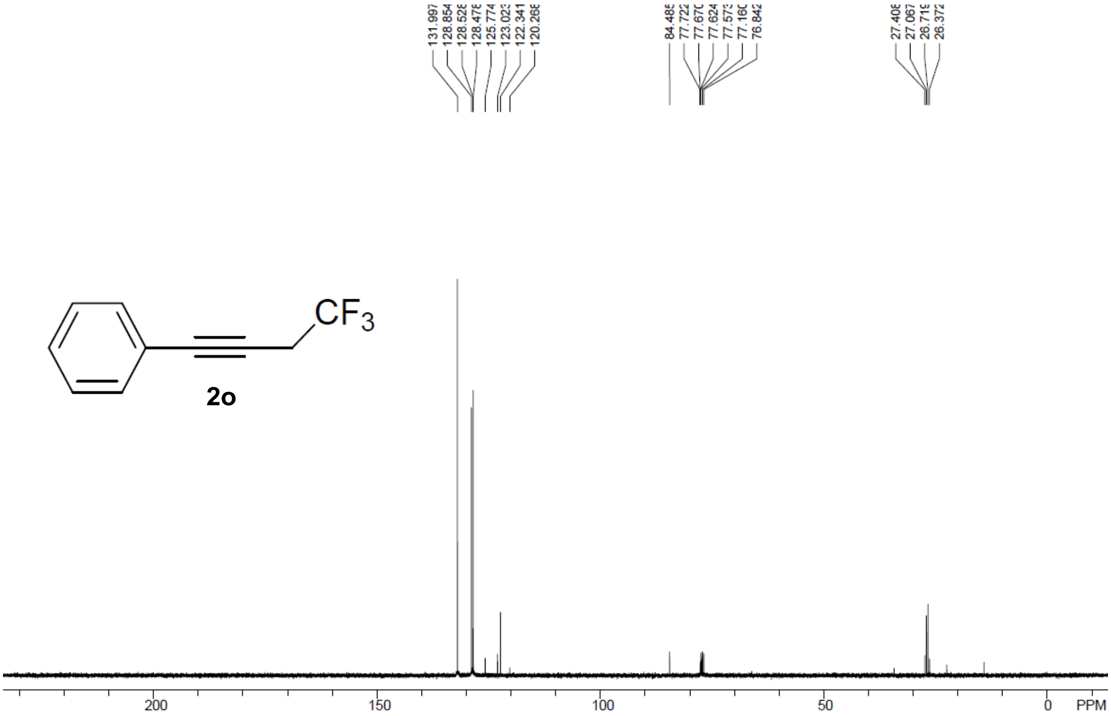
CF3
2o
200
150
100
50
0
PPM

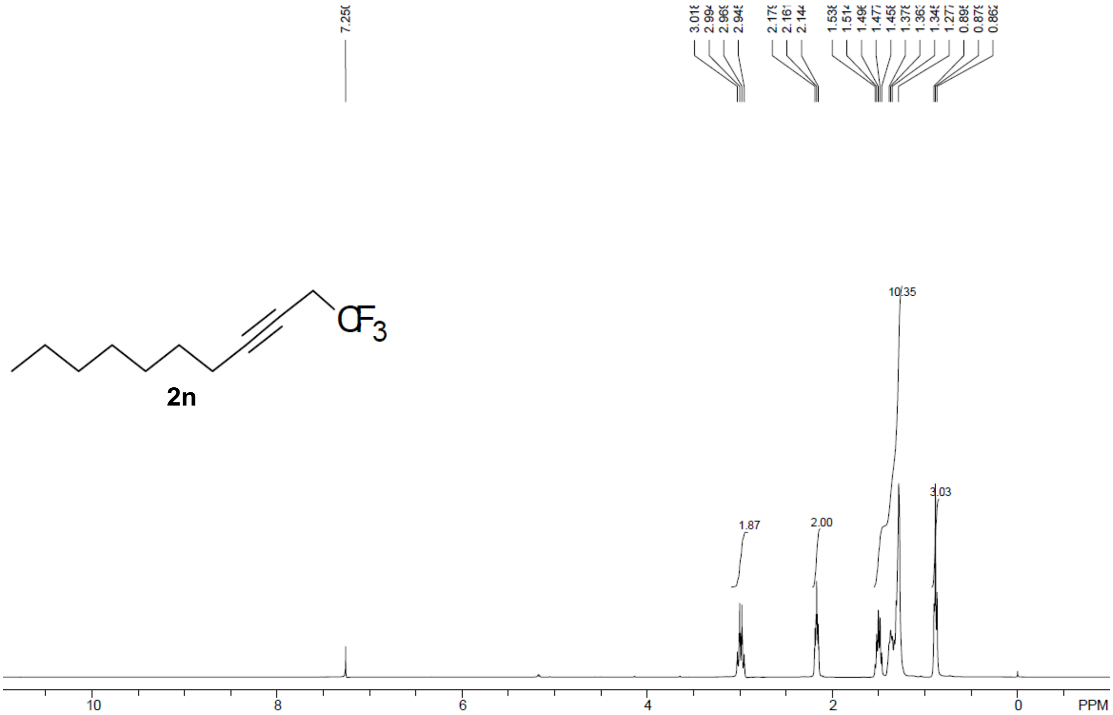
CF3
2n
1.87
2.00
10.35
3.03
10
8
6
4
2
0
PPM

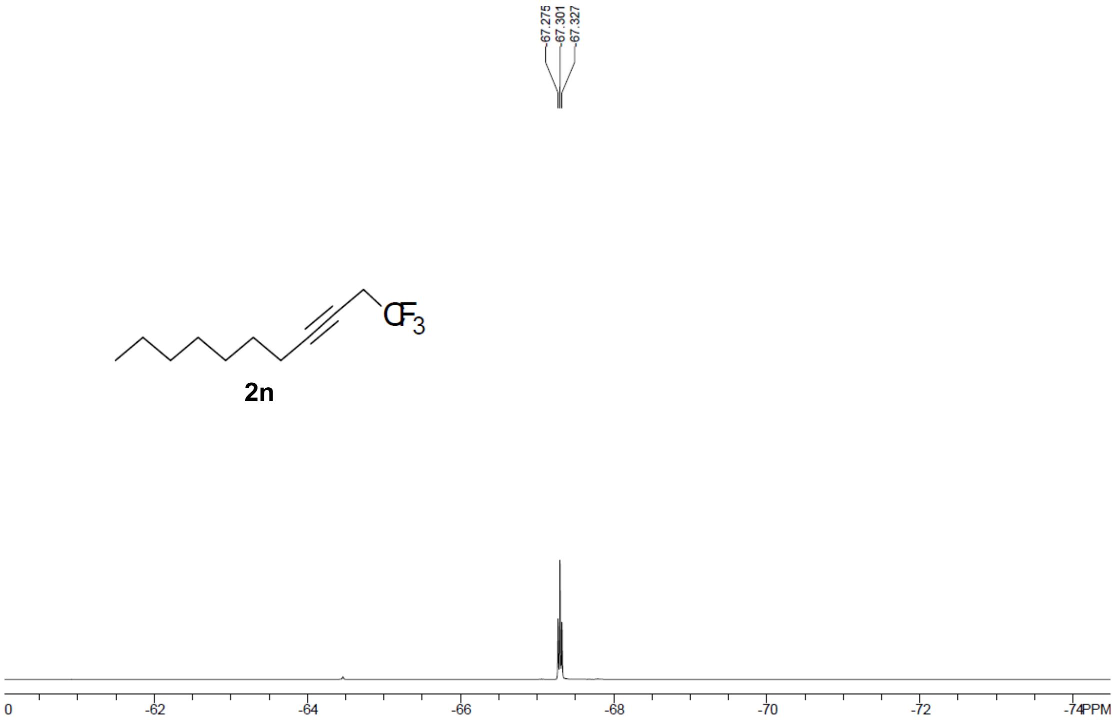
-67.275
-67.301
-67.327
CF3
2n
0
-62
-64
-66
-68
-70
-72
-74
PPM

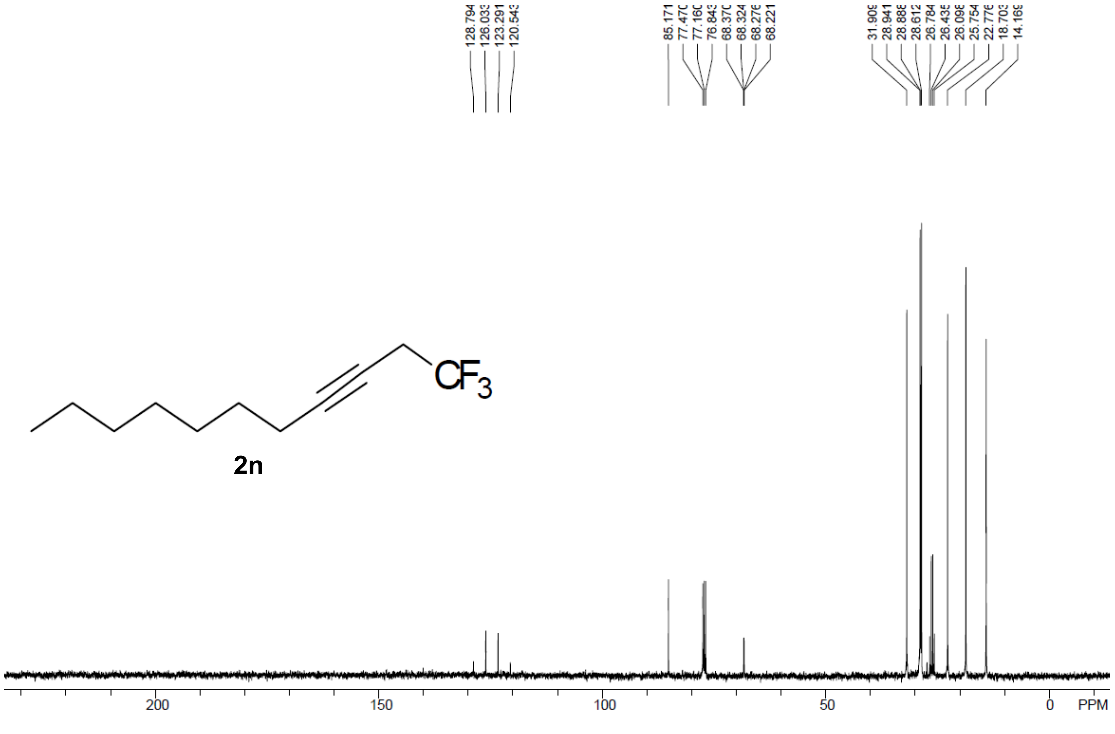
128.794
126.033
123.291
120.543
85.171
77.470
77.160
76.843
68.370
68.324
68.276
68.221
31.909
28.941
28.888
28.612
26.784
26.435
26.098
25.754
22.776
18.703
14.169
CF3
2n
200
150
100
50
0
PPM

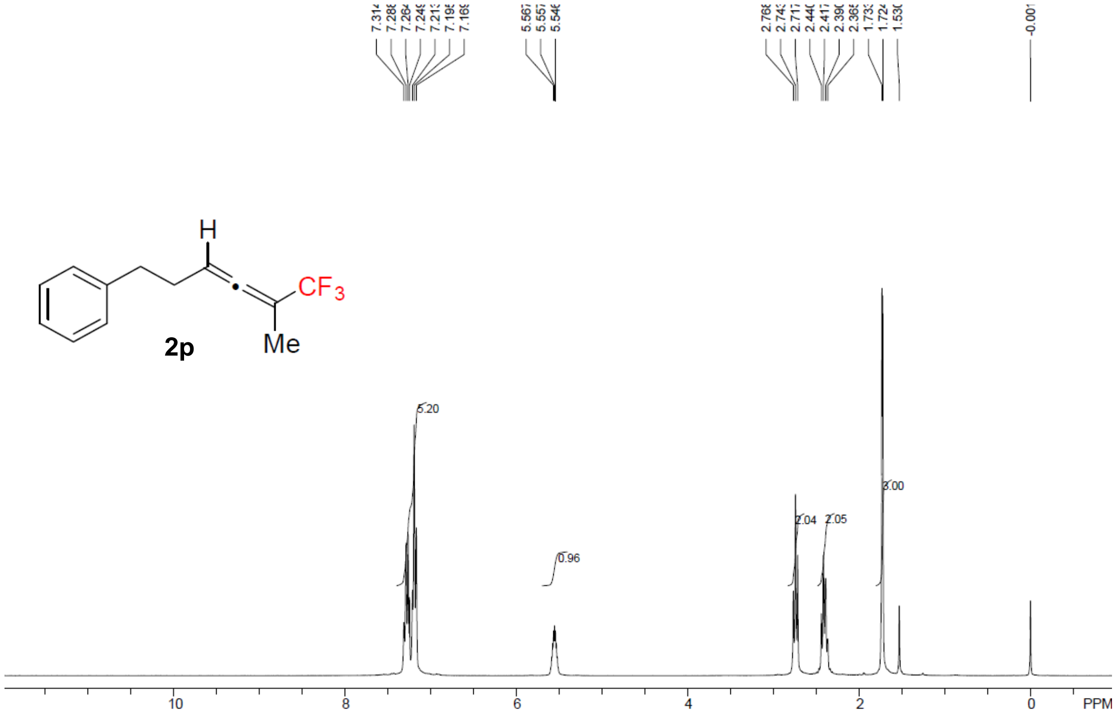
H
CF3
2p
Me
5.20
0.96
2.04
2.05
3.00
-0.001
10
8
6
4
2
0
PPM

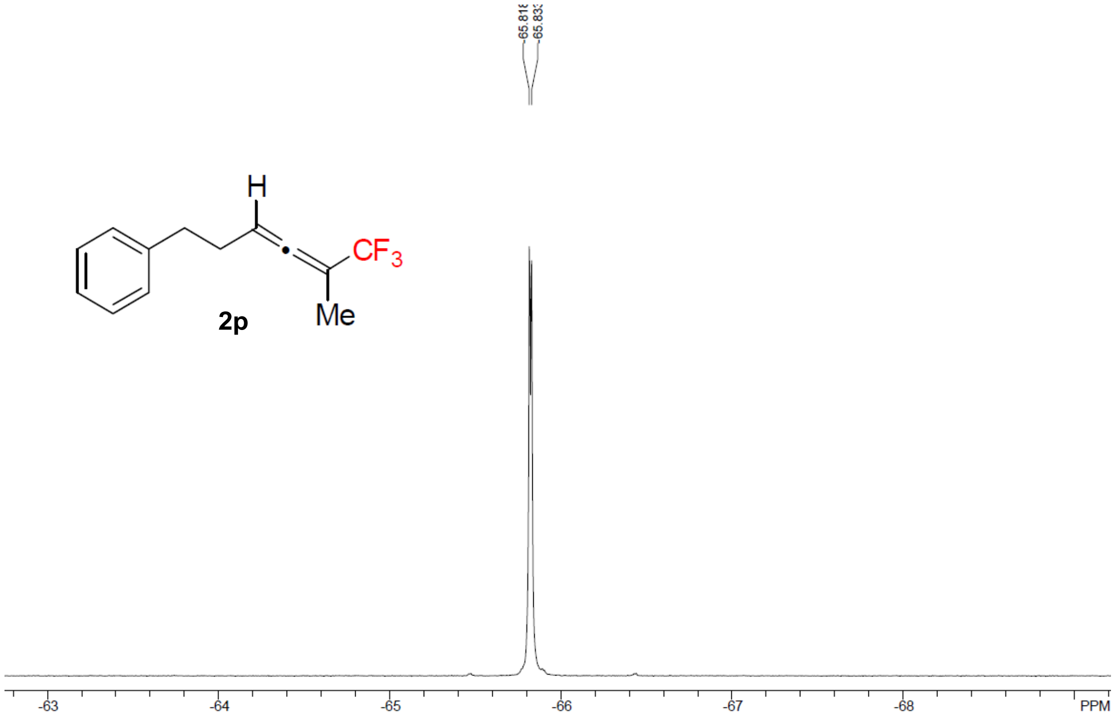
H
CF3
2p
Me
-63
-64
-65
-66
-67
-68
PPM

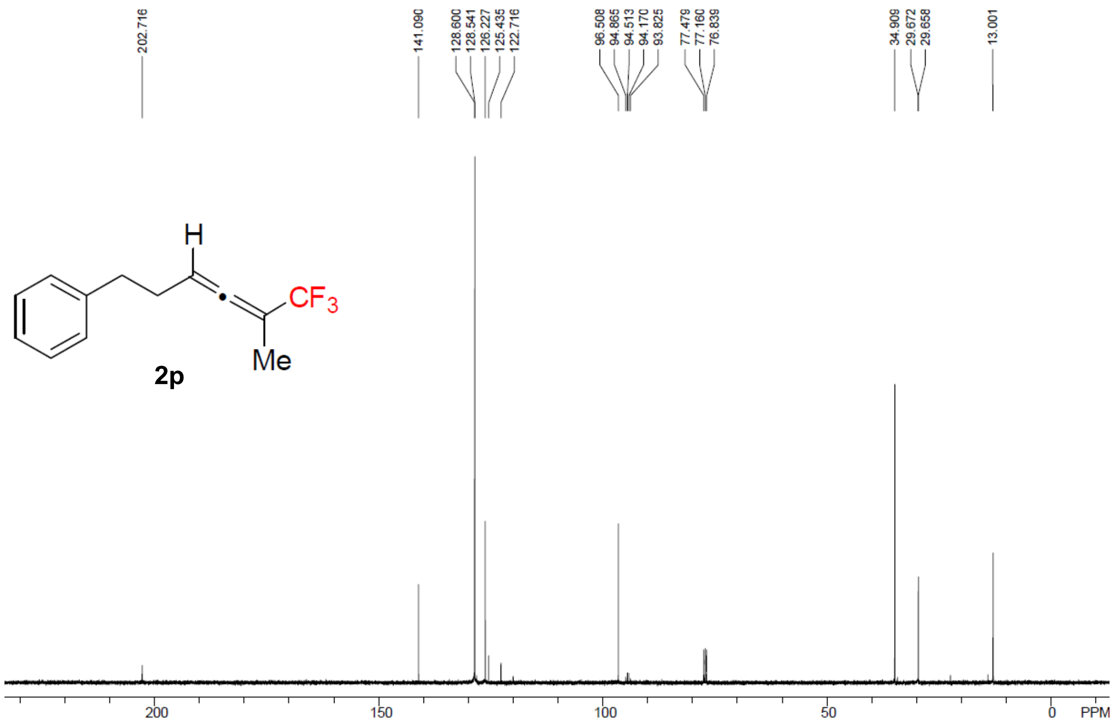

202.716
141.090
128.600
128.541
126.227
125.435
122.716
96.508
94.865
94.513
94.170
93.825
77.479
77.160
76.839
34.909
29.672
29.658
13.001
H
CF3
Me
2p
200
150
100
50
0
PPM

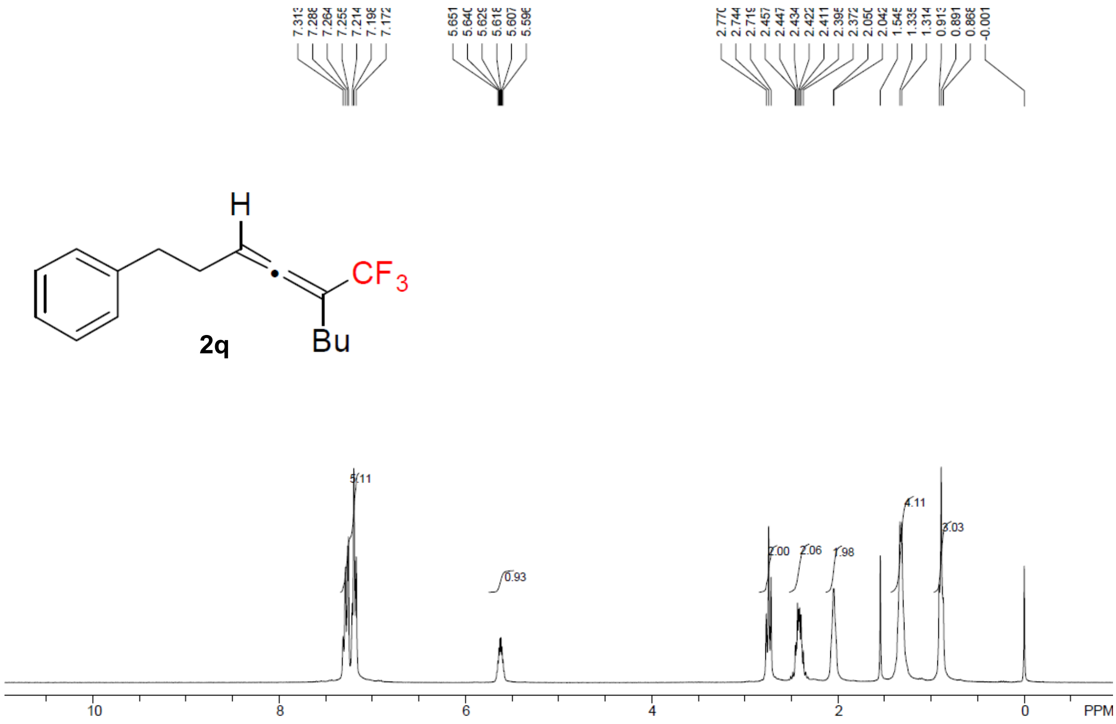

5.651
5.640
2.744
2.457
2.447
2.434
2.411
H
CF3
Bu
2q
5.11
0.93
2.00
2.06
1.98
4.11
3.03
10
8
6
4
2
0
PPM

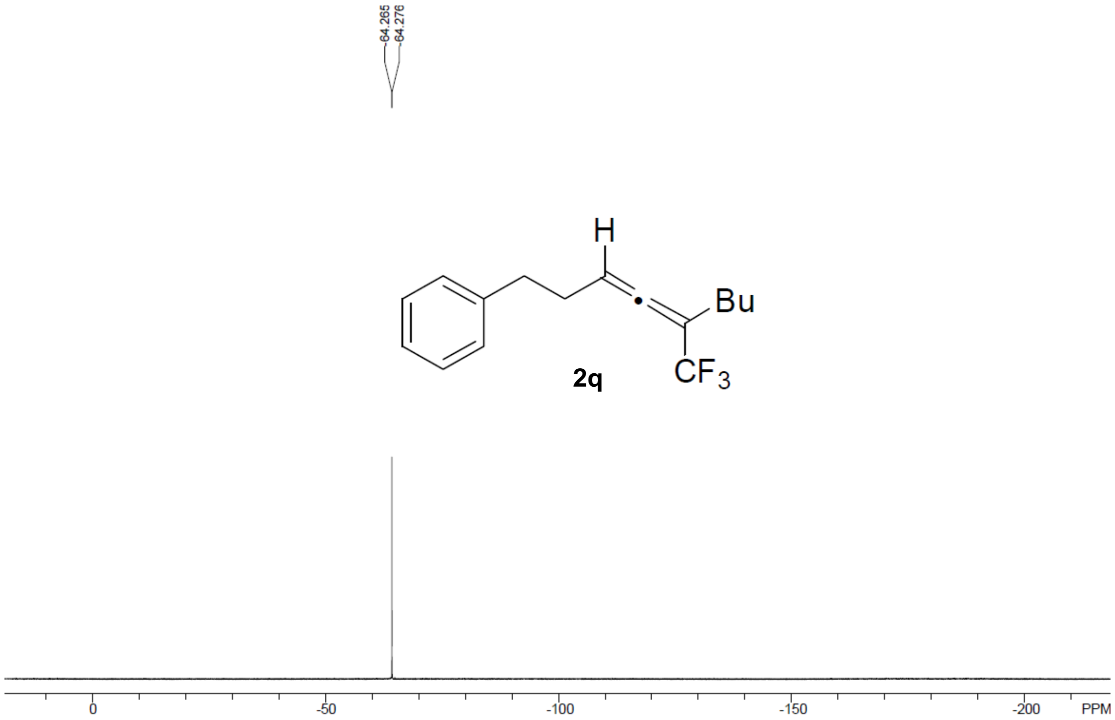
-64.265
-64.276
H
Bu
2q
CF3
0
-50
-100
-150
-200
PPM

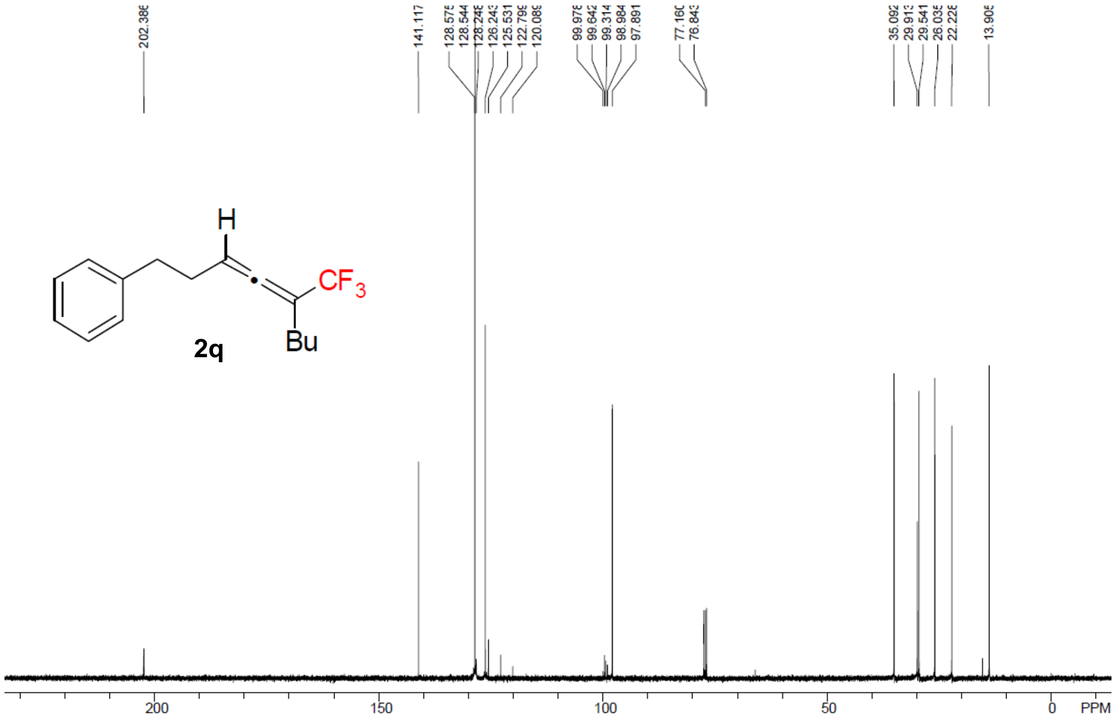
141.117
128.544
125.531
120.089
99.642
99.314
98.984
97.891
35.092
29.541
13.905
H
CF3
2q
Bu
200
150
100
50
0
PPM

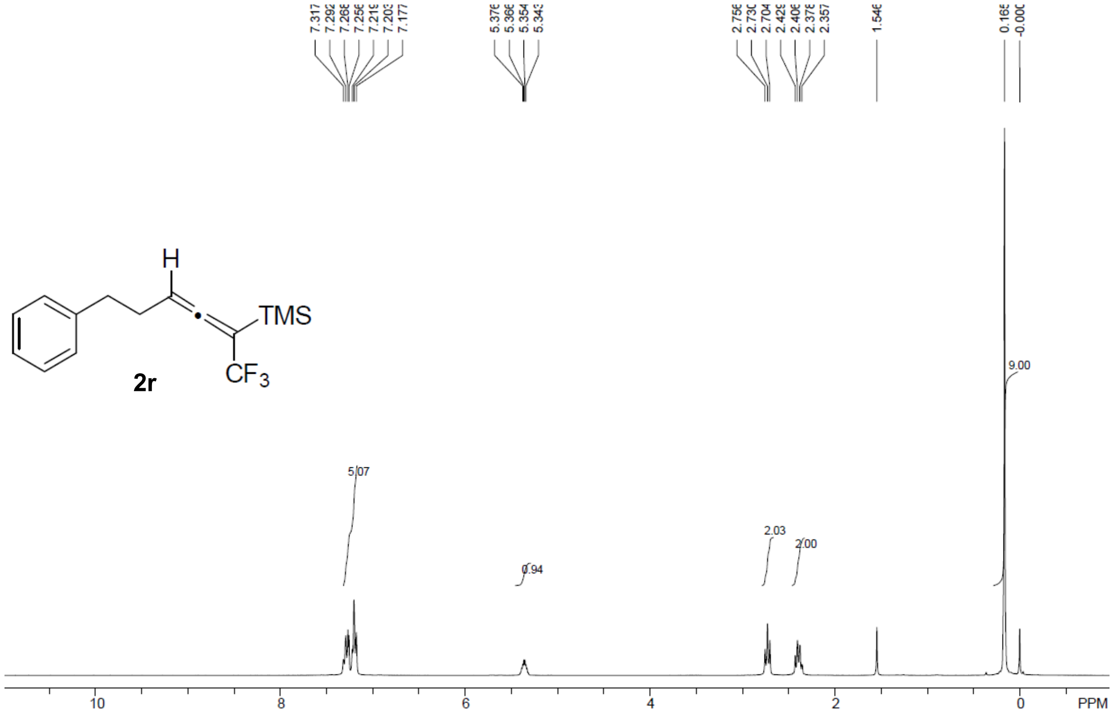

7.317
7.292
7.268
7.256
7.219
7.203
7.177
5.376
5.366
5.354
5.343
2.756
2.730
2.704
2.429
2.406
2.378
2.357
1.546
0.165
-0.000
H
TMS
CF3
2r
5.07
0.94
2.03
2.00
9.00
10
8
6
4
2
0
PPM

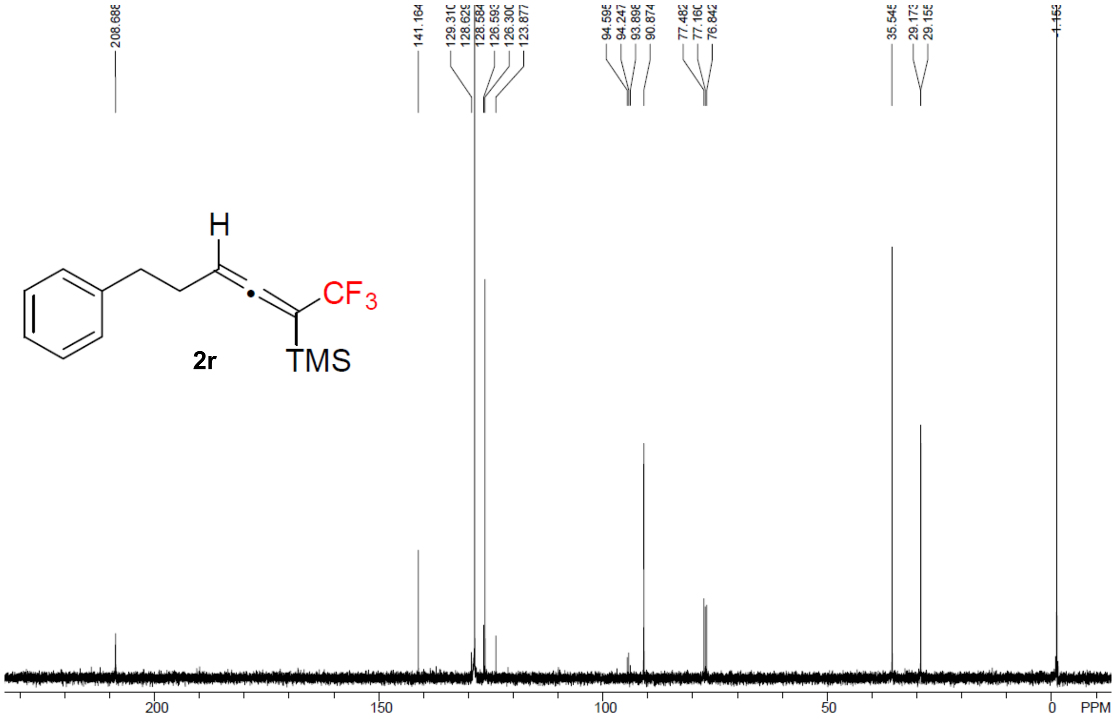

208.688
141.164
129.310
128.629
128.584
126.593
126.300
123.877
94.595
94.247
93.898
90.874
77.482
77.160
76.842
35.545
29.173
29.155
-1.153
H
CF3
2r
TMS
200
150
100
50
0
PPM

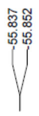

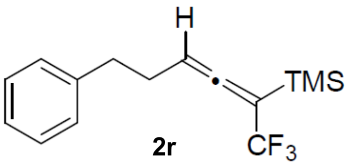

2r

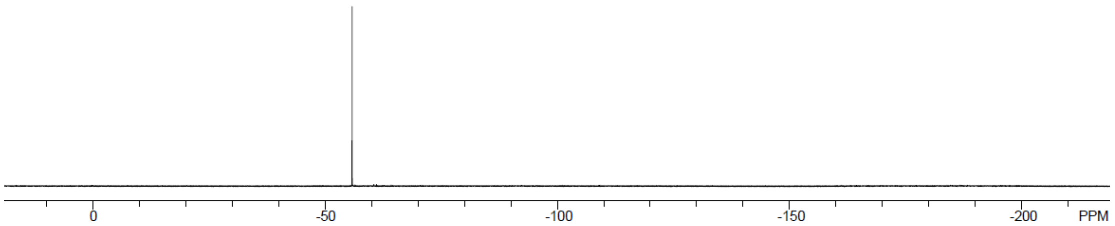

Supplement: File 1 — Experimental details, characterization data of all products and copies of NMR spectra. [file Beilstein_J_Org_Chem-09-2862-s001.pdf]
